# Model fitting data from syllogistic reasoning experiments

**DOI:** 10.1016/j.dib.2016.09.053

**Published:** 2016-10-14

**Authors:** Masasi Hattori

**Affiliations:** College of Comprehensive Psychology, Ritsumeikan University, Ibaraki, Osaka 567–8570, Japan

**Keywords:** Deduction, Probabilistic inference, Syllogistic reasoning, Mental representation, Information gain, Symmetry inference

## Abstract

The data presented in this article are related to the research article entitled “Probabilistic representation in syllogistic reasoning: A theory to integrate mental models and heuristics” (M. Hattori, 2016) [1]. This article presents predicted data by three signature probabilistic models of syllogistic reasoning and model fitting results for each of a total of 12 experiments (*N*=404) in the literature. Models are implemented in R, and their source code is also provided.

**Specifications Table**

**Table:** 

Subject area	Psychology
More specific subject area	Cognitive Psychology, Psychology of Thinking
Type of data	Tables, R code
How data was acquired	Laboratory experiments and model fitting
Data format	Raw and analyzed
Experimental factors	Various types of syllogism
Experimental features	Output of syllogistic reasoning
Data source location	Japan and other countries
Data accessibility	Available with this article

**Value of the data**

• Data provided in a unified form are useful for prospective meta-analyses, given that definitions of syllogistic form are confused in the psychology literature.
• Model fitting results indicate whether and how one model is better and could be suggestive for developing new models.
• Model R code could contribute to a deeper understanding of the theory and its new developments.

## 1. Data

The dataset of this article provides participants’ response proportions as logical conclusions for each syllogism, model predictions for each experiment, and goodness-of-fit statistics of models. Table 1, Table 2 show the comparison of the probabilistic representation theory (PRT) [1] with other models (the transitive-chain theory (TCT) [2] and the probability heuristic model (PHM) [3], respectively). Table 3, Table 4, Table 5, Table 6, Table 7, Table 8, Table 9 show the comparison of three models (i.e., PRT, PHM, and a probabilistic extension of the mental model theory [pMM] [1], [4]) using data of Experiment 1 in [5], Experiment 2 in [5], Experiment 2, first Test in [4], Experiment 2, second Test in [4], Experiment 1 in [2], Experiment 3 in [6], Experiment with adult participants in [7], Experiment in [8], Experiment 1 in [1], and Experiment 2 in [1], respectively. Tables 13 and 14 show the comparison of PRT and PHM using data from syllogisms with generalized quantifiers, Experiment 1 in [3] and Experiment 2 in [3], respectively.

## 2. Experimental design, materials and methods

In this article, syllogistic terminology is according to the orthodox Aristotelian manner. Standard syllogisms are constructed with two premises and one conclusion, each belonging to one of the following four *moods*:

A: All X are Y
I: Some X are Y
E: No X are Y
O: Some X are not Y

The subject (S) and predicate (P) in the conclusion are called end terms, and a term that does not appear in the conclusion is called a middle term (M). The two terms X and Y in the first premise correspond to P and M, or M and P; similarly, X and Y in the second premise correspond to S and M, or M and S. As each premise has two possibilities for correspondence, the relative positions of these terms can be one of the subsequent four possibilities, called *figures*:

**Table:** 

Figure 1	Figure 2	Figure 3	Figure 4
M–P	P–M	M–P	P–M
S–M	S–M	M–S	M–S
S–P	S–P	S–P	S–P

The type of syllogism is indicated by symbols such as AI2, which indicates the first premise as A, the second premise as I, and the corresponding figure 2.

Participants are given all or a considerable part of the entire 64 variations of premise pairs, and they select (or generate) a sentence as a logically valid conclusion. For example, in a typical experiment, participants are sequentially given a pair of premises such as (IO2):

Some practitioners are mediators.
Some sophists are not mediators.

Participants are supposed to choose one from the following conclusion candidates:

A: All sophists are practitioners.
I: Some sophists are practitioners.
E: No sophists are practitioners.
O: Some sophists are not practitioners.
N: No valid conclusion.

The data set provides the proportion for each response (i.e., A, I, E, O, or N). In some experiments [1], [2], [4], [5], choice options were given to participants as instantiated above, while in other experiments [3], [6], [7], [8], participants were asked to generate their own conclusions.

PRT and PHM can predict individual performance on syllogisms with generalized quantifiers, including “most …” and “few ….” For the evaluation of these models, some tasks (shown in Tables 13 and 14) included the following types of assertions in addition to the standard ones:

M: Most sophists are practitioners.
F: Few sophists are practitioners.

## Figures and Tables

**Table 1:** Data from Guyote and Sternberg׳s [2] Experiment 1 and models’ predictions.

Type	Data (*N*=49)					TCT							PRT						
	A	I	E	O	N	A	I	E	O	N	RMSD	*r*	A	I	E	O	N	RMSD	*r*
AA1	.97	.02	.00	.00	.00	.92	.08	.00	.00	.00	.0349	.9978	.8724	.0000	.0000	.0000	.1276	.0724	.9882
AA2	.47	.04	.00	.00	.47	.54	.09	.00	.00	.37	.0590	.9649	.5326	.3230	.0008	.0062	.1373	.1974	.5779
AA3	.52	.33	.00	.02	.11	.54	.32	.00	.00	.14	.0190	.9964	.5316	.3386	.0000	.0009	.1289	.0137	.9985
AA4	.42	.48	.02	.00	.06	.50	.50	.00	.00	.00	.0465	.9916	.2512	.6071	.0003	.0098	.1316	.1003	.8946
AI1	.07	.85	.00	.04	.02	.05	.89	.00	.05	.01	.0210	.9995	.0027	.7929	.0073	.0583	.1389	.0668	.9812
AI2	.00	.69	.00	.04	.26	.05	.70	.00	.05	.20	.0355	.9910	.0011	.7025	.0331	.1244	.1388	.0679	.9665
AI3	.00	.88	.02	.02	.07	.05	.89	.00	.05	.01	.0387	.9937	.0027	.7929	.0073	.0583	.1389	.0529	.9947
AI4	.04	.54	.04	.02	.33	.05	.70	.00	.05	.20	.0950	.9407	.0011	.7025	.0331	.1244	.1388	.1228	.8813
IA1	.02	.80	.00	.00	.16	.05	.70	.00	.05	.20	.0548	.9964	.0051	.7025	.0331	.1104	.1489	.0680	.9875
IA2	.02	.72	.00	.04	.20	.05	.70	.00	.05	.20	.0167	.9992	.0051	.7025	.0331	.1104	.1489	.0428	.9885
IA3	.00	.97	.00	.00	.02	.05	.89	.00	.05	.01	.0480	.9975	.0006	.7929	.0073	.0658	.1335	.0986	.9898
IA4	.02	.88	.00	.00	.09	.05	.89	.00	.05	.01	.0445	.9917	.0006	.7929	.0073	.0658	.1335	.0534	.9949
AE1	.00	.02	.59	.09	.28	.00	.00	.50	.04	.46	.0932	.9151	.0000	.0008	.6521	.2173	.1298	.0927	.9229
AE2	.00	.00	.88	.04	.07	.00	.00	.92	.08	.00	.0402	.9950	.0000	.0000	.8724	.0000	.1276	.0315	.9958
AE3	.02	.07	.47	.09	.33	.00	.00	.50	.04	.46	.0716	.9747	.0000	.0008	.6521	.2173	.1298	.1376	.8271
AE4	.02	.00	.90	.02	.04	.00	.00	.92	.07	.00	.0313	.9965	.0000	.0000	.8724	.0000	.1276	.0430	.9930
EA1	.02	.00	.90	.04	.02	.00	.00	.92	.08	.00	.0237	.9982	.0000	.0000	.8724	.0000	.1276	.0535	.9887
EA2	.00	.04	.85	.07	.02	.00	.00	.92	.08	.00	.0374	.9989	.0000	.0000	.8724	.0000	.1276	.0610	.9840
EA3	.00	.02	.45	.28	.23	.00	.00	.50	.33	.17	.0424	.9824	.0000	.0002	.6521	.2194	.1283	.1051	.9282
EA4	.02	.01	.52	.28	.16	.00	.01	.50	.33	.17	.0261	.9909	.0000	.0002	.6521	.2194	.1283	.0673	.9796
AO1	.00	.07	.00	.62	.30	.00	.02	.05	.60	.33	.0355	.9889	.0326	.1236	.0011	.7038	.1389	.0859	.9428
AO2	.00	.07	.00	.80	.11	.00	.02	.05	.87	.06	.0498	.9926	.0071	.0578	.0027	.7935	.1388	.0147	.9990
AO3	.00	.07	.02	.57	.33	.00	.02	.05	.60	.33	.0293	.9932	.0326	.1236	.0011	.7038	.1389	.1084	.9084
AO4	.00	.04	.00	.64	.30	.00	.02	.05	.63	.29	.0249	.9955	.0071	.0578	.0027	.7935	.1388	.0999	.9515
OA1	.00	.02	.00	.76	.21	.00	.02	.05	.65	.27	.0603	.9894	.0326	.1095	.0052	.7038	.1489	.0566	.9868
OA2	.00	.02	.02	.61	.33	.00	.02	.05	.60	.33	.0141	.9989	.0326	.1095	.0052	.7038	.1489	.1009	.9201
OA3	.00	.07	.00	.83	.09	.00	.02	.05	.87	.06	.0387	.9944	.0071	.0650	.0006	.7938	.1335	.0256	.9982
IE3	.00	.02	.21	.23	.52	.00	.00	.10	.44	.46	.1097	.8506	.0111	.0624	.0971	.5405	.2888	.1814	.5494
IE4	.00	.02	.11	.38	.47	.00	.00	.10	.44	.46	.0290	.9924	.0111	.0624	.0971	.5405	.2888	.1102	.8383
EI1	.02	.04	.07	.69	.16	.00	.00	.10	.65	.24	.0467	.9828	.0111	.0624	.0971	.5405	.2888	.0897	.9513
EI2	.00	.04	.07	.73	.14	.00	.00	.10	.65	.24	.0615	.9774	.0111	.0624	.0971	.5405	.2888	.1090	.9429
EI3	.00	.02	.09	.64	.23	.00	.00	.10	.65	.24	.0118	.9992	.0111	.0624	.0971	.5405	.2888	.0554	.9858
EI4	.02	.02	.14	.47	.35	.00	.00	.10	.65	.24	.0969	.9363	.0111	.0624	.0971	.5405	.2888	.0499	.9672
IO4	.00	.04	.00	.64	.30	.00	.02	.05	.60	.33	.0329	.9929	.0128	.0722	.0015	.6247	.2888	.0177	.9988
EE3	.00	.00	.04	.00	.95	.00	.00	.00	.00	1.00	.0286	.9992	.1262	.0018	.0011	.0018	.8692	.0693	.9869
EO3	.00	.07	.00	.23	.69	.00	.00	.00	.17	.83	.0750	.9893	.0023	.0131	.0003	.1134	.8709	.0996	.9784
EO4	.00	.02	.07	.19	.71	.00	.00	.00	.17	.83	.0634	.9958	.0023	.0131	.0003	.1134	.8709	.0856	.9894
OE3	.00	.02	.02	.06	.90	.00	.00	.00	.00	1.00	.0537	.9985	.0023	.0131	.0003	.1134	.8709	.0288	.9972
Mean											.0458	.9936						.0773	.9788

**Table 2:** Data from Chater and Oaksford׳s [3] Meta-analysis and models’ predictions.

Type	Data (*N*=158)					PHM							PRT						
	A	I	E	O	N	A	I	E	O	N	RMSD	*r*	A	I	E	O	N	RMSD	*r*
AA1	.8987	.0506	.0000	.0000	.0190	.7014	.1076	.0122	.0122	.1666	.1134	.9797	.8240	.0000	.0000	.0000	.1760	.0810	.9761
AA2	.5823	.0823	.0127	.0127	.2848	.7014	.1076	.0122	.0122	.1666	.0759	.9629	.6032	.2123	.0003	.0022	.1820	.0751	.9415
AA3	.5696	.2911	.0000	.0000	.1392	.7014	.1076	.0122	.0122	.1666	.1021	.9227	.5738	.2473	.0000	.0009	.1780	.0262	.9925
AA4	.7532	.1646	.0127	.0063	.0443	.7014	.1076	.0122	.0122	.1666	.0647	.9764	.3790	.4346	.0002	.0055	.1807	.2152	.6535
AI1	.0000	.9241	.0253	.0253	.0253	.0122	.7014	.0122	.1076	.1666	.1238	.9765	.0030	.7047	.0173	.0845	.1905	.1257	.9707
AI2	.0000	.5696	.0253	.1076	.2911	.0122	.7014	.0122	.1076	.1666	.0815	.9584	.0017	.6396	.0413	.1275	.1899	.0562	.9707
AI3	.0127	.8861	.0127	.0253	.0443	.0122	.7014	.0122	.1076	.1666	.1057	.9804	.0030	.7047	.0173	.0845	.1905	.1076	.9752
AI4	.0000	.7089	.0000	.0127	.2658	.0122	.7014	.0122	.1076	.1666	.0620	.9751	.0017	.6396	.0413	.1275	.1899	.0714	.9762
IA1	.0000	.7152	.0000	.0633	.2152	.0122	.7014	.0122	.1076	.1666	.0310	.9942	.0042	.6396	.0413	.1186	.1963	.0466	.9957
IA2	.1329	.4873	.0253	.1203	.3101	.0122	.7014	.0122	.1076	.1666	.1275	.9133	.0042	.6396	.0413	.1186	.1963	.1029	.9191
IA3	.0190	.8481	.0063	.0380	.0570	.0122	.7014	.0122	.1076	.1666	.0877	.9838	.0010	.7047	.0173	.0917	.1853	.0899	.9793
IA4	.0000	.9114	.0127	.0063	.0443	.0122	.7014	.0122	.1076	.1666	.1179	.9802	.0010	.7047	.0173	.0917	.1853	.1183	.9766
AE1	.0000	.0253	.5949	.0570	.3165	.0122	.0122	.7014	.1076	.1666	.0856	.9449	.0000	.0004	.6847	.1369	.1780	.0827	.9458
AE2	.0000	.0000	.8797	.0127	.0570	.0122	.0122	.7014	.1076	.1666	.1031	.9844	.0000	.0000	.8240	.0000	.1760	.0590	.9878
AE3	.0000	.0127	.6139	.1329	.2278	.0122	.0122	.7014	.1076	.1666	.0494	.9885	.0000	.0004	.6847	.1369	.1780	.0391	.9936
AE4	.0000	.0253	.8671	.0190	.0253	.0122	.0122	.7014	.1076	.1666	.1054	.9763	.0000	.0000	.8240	.0000	.1760	.0715	.9792
EA1	.0000	.0127	.8671	.0253	.0443	.0122	.0122	.7014	.1076	.1666	.0993	.9820	.0000	.0000	.8240	.0000	.1760	.0632	.9838
EA2	.0000	.0000	.8861	.0316	.0380	.0122	.0122	.7014	.1076	.1666	.1065	.9826	.0000	.0000	.8240	.0000	.1760	.0691	.9825
EA3	.0000	.0000	.6392	.2152	.1203	.0122	.0122	.7014	.1076	.1666	.0598	.9745	.0000	.0002	.6847	.1382	.1770	.0474	.9837
EA4	.0127	.0253	.6139	.0823	.2405	.0122	.0122	.7014	.1076	.1666	.0528	.9855	.0000	.0002	.6847	.1382	.1770	.0509	.9843
AO1	.0127	.0633	.0127	.5696	.3101	.0122	.1076	.0122	.7014	.1666	.0894	.9432	.0243	.1002	.0025	.6826	.1905	.0757	.9573
AO2	.0000	.0633	.0253	.6709	.2152	.0122	.1076	.0122	.7014	.1666	.0334	.9920	.0098	.0622	.0040	.7341	.1899	.0322	.9975
AO3	.0000	.1013	.0000	.6646	.1899	.0122	.1076	.0122	.7014	.1666	.0212	.9980	.0243	.1002	.0025	.6826	.1905	.0136	.9992
AO4	.0000	.0506	.0253	.7152	.1962	.0122	.1076	.0122	.7014	.1666	.0304	.9941	.0098	.0622	.0040	.7341	.1899	.0147	.9990
OA1	.0000	.0316	.0253	.6772	.2342	.0122	.1076	.0122	.7014	.1666	.0474	.9833	.0243	.0909	.0059	.6826	.1963	.0345	.9913
OA2	.0000	.1139	.0506	.5633	.2278	.0122	.1076	.0122	.7014	.1666	.0700	.9843	.0243	.0909	.0059	.6826	.1963	.0606	.9885
OA3	.0000	.1456	.0316	.6899	.0823	.0122	.1076	.0122	.7014	.1666	.0429	.9868	.0085	.0642	.0016	.7405	.1853	.0644	.9753
OA4	.0127	.0253	.0633	.2722	.3291	.0122	.1076	.0122	.7014	.1666	.2098	.6356	.0085	.0642	.0016	.7405	.1853	.2215	.6693
II1	.0000	.4051	.0253	.0443	.5063	.0122	.3111	.0122	.1076	.5569	.0561	.9655	.0013	.4019	.0166	.0638	.5164	.0107	.9989
II2	.0127	.4241	.0316	.0253	.4937	.0122	.3111	.0122	.1076	.5569	.0691	.9472	.0013	.4019	.0166	.0638	.5164	.0238	.9939
II3	.0000	.2405	.0253	.0127	.7152	.0122	.3111	.0122	.1076	.5569	.0887	.9667	.0013	.4019	.0166	.0638	.5164	.1168	.9120
II4	.0000	.4241	.0000	.0127	.5696	.0122	.3111	.0122	.1076	.5569	.0667	.9698	.0013	.4019	.0166	.0638	.5164	.0353	.9968
IE1	.0127	.0127	.2215	.1646	.5696	.0122	.0122	.3111	.1076	.5569	.0478	.9737	.0073	.0383	.0903	.3476	.5164	.1041	.8668
IE2	.0000	.0000	.3924	.3038	.2722	.0122	.0122	.3111	.1076	.5569	.1590	.6609	.0073	.0383	.0903	.3476	.5164	.1757	.5433
IE3	.0000	.0127	.2975	.3291	.3481	.0122	.0122	.3111	.1076	.5569	.1364	.7584	.0073	.0383	.0903	.3476	.5164	.1202	.7960
IE4	.0000	.0063	.2785	.4430	.2405	.0122	.0122	.3111	.1076	.5569	.2068	.4203	.0073	.0383	.0903	.3476	.5164	.1560	.6519
EI1	.0000	.0506	.1456	.6646	.1392	.0122	.0122	.3111	.1076	.5569	.3205	-.0202	.0073	.0383	.0903	.3476	.5164	.2218	.4979
EI2	.0127	.0127	.2089	.5190	.2152	.0122	.0122	.3111	.1076	.5569	.2435	.2438	.0073	.0383	.0903	.3476	.5164	.1642	.6368
EI3	.0000	.0633	.1519	.4810	.2722	.0122	.0122	.3111	.1076	.5569	.2230	.3238	.0073	.0383	.0903	.3476	.5164	.1280	.7701
EI4	.0000	.0190	.3165	.2658	.3734	.0122	.0122	.3111	.1076	.5569	.1086	.8637	.0073	.0383	.0903	.3476	.5164	.1254	.7756
IO1	.0253	.0380	.0127	.3038	.6076	.0122	.1076	.0122	.3111	.5569	.0391	.9896	.0090	.0470	.0019	.4257	.5164	.0688	.9551
IO2	.0127	.0506	.0380	.3671	.4873	.0122	.1076	.0122	.3111	.5569	.0488	.9739	.0090	.0470	.0019	.4257	.5164	.0335	.9963
IO3	.0000	.0949	.0127	.2911	.5886	.0122	.1076	.0122	.3111	.5569	.0185	.9980	.0090	.0470	.0019	.4257	.5164	.0719	.9480
IO4	.0000	.0506	.0127	.4430	.4684	.0122	.1076	.0122	.3111	.5569	.0757	.9364	.0090	.0470	.0019	.4257	.5164	.0237	.9954
OI1	.0380	.0633	.0000	.3544	.5380	.0122	.1076	.0122	.3111	.5569	.0317	.9887	.0090	.0470	.0019	.4257	.5164	.0365	.9876
OI2	.0000	.0823	.0253	.3544	.5000	.0122	.1076	.0122	.3111	.5569	.0349	.9873	.0090	.0470	.0019	.4257	.5164	.0380	.9913
OI3	.0127	.0886	.0127	.3101	.5633	.0122	.1076	.0122	.3111	.5569	.0090	.9993	.0090	.0470	.0019	.4257	.5164	.0590	.9647
OI4	.0253	.0759	.0190	.2911	.5506	.0122	.1076	.0122	.3111	.5569	.0182	.9970	.0090	.0470	.0019	.4257	.5164	.0643	.9594
EE1	.0000	.0127	.3418	.0127	.6076	.0122	.0122	.1878	.1076	.6802	.0873	.9375	.1102	.0614	.0077	.0993	.7214	.1712	.7745
EE2	.0253	.0253	.1392	.0316	.7722	.0122	.0122	.1878	.1076	.6802	.0582	.9882	.1102	.0614	.0077	.0993	.7214	.0812	.9616
EE3	.0000	.0000	.1772	.0253	.7785	.0122	.0122	.1878	.1076	.6802	.0580	.9940	.1102	.0614	.0077	.0993	.7214	.1033	.9401
EE4	.0000	.0253	.3101	.0063	.6329	.0122	.0122	.1878	.1076	.6802	.0745	.9552	.1102	.0614	.0077	.0993	.7214	.1558	.8160
EO1	.0127	.0759	.0759	.2342	.5949	.0122	.1076	.0122	.1878	.6802	.0538	.9860	.0051	.0267	.0011	.2424	.7246	.0707	.9947
EO2	.0000	.1329	.0696	.1139	.6519	.0122	.1076	.0122	.1878	.6802	.0455	.9846	.0051	.0267	.0011	.2424	.7246	.0869	.9567
EO3	.0000	.0000	.0886	.2848	.5823	.0122	.1076	.0122	.1878	.6802	.0855	.9412	.0051	.0267	.0011	.2424	.7246	.0780	.9764
EO4	.0000	.0506	.0823	.1203	.7025	.0122	.1076	.0122	.1878	.6802	.0517	.9806	.0051	.0267	.0011	.2424	.7246	.0672	.9715
OE1	.0127	.0000	.1392	.0506	.7722	.0122	.1076	.0122	.1878	.6802	.1049	.9378	.0051	.0267	.0011	.2424	.7246	.1085	.9290
OE2	.0000	.0759	.1076	.1646	.6266	.0122	.1076	.0122	.1878	.6802	.0523	.9820	.0051	.0267	.0011	.2424	.7246	.0768	.9770
OE3	.0000	.0506	.1203	.1772	.6076	.0122	.1076	.0122	.1878	.6802	.0640	.9724	.0051	.0267	.0011	.2424	.7246	.0809	.9767
OE4	.0000	.1899	.0949	.1392	.5633	.0122	.1076	.0122	.1878	.6802	.0772	.9703	.0051	.0267	.0011	.2424	.7246	.1201	.9314
OO1	.0127	.0759	.0127	.2215	.6646	.0122	.1076	.0122	.1804	.6876	.0254	.9952	.0052	.0272	.0011	.2461	.7204	.0355	.9974
OO2	.0000	.1646	.0506	.1013	.6835	.0122	.1076	.0122	.1804	.6876	.0472	.9823	.0052	.0272	.0011	.2461	.7204	.0935	.9418
OO3	.0127	.0633	.0000	.1519	.7658	.0122	.1076	.0122	.1804	.6876	.0425	.9968	.0052	.0272	.0011	.2461	.7204	.0496	.9856
OO4	.0127	.0443	.0063	.2468	.6456	.0122	.1076	.0122	.1804	.6876	.0452	.9845	.0052	.0272	.0011	.2461	.7204	.0346	.9993
Mean											.0824	.9728						.0829	.9733

**Table 3:** Data from Dickstein׳s [5] Experiment 1 and models’ predictions.

Type	Data (*N*=22)					PRT							PHM							pMM						
	A	I	E	O	N	A	I	E	O	N	RMSD	*r*	A	I	E	O	N	RMSD	*r*	A	I	E	O	N	RMSD	*r*
AA1	.9545	.0000	.0000	.0000	.0455	.8967	.0000	.0000	.0000	.1033	.0366	.9977	.8059	.0502	.0060	.0060	.1318	.0802	.9942	.9305	.0174	.0174	.0174	.0174	.0213	.9989
AA2	.7727	.0000	.0000	.0000	.2273	.6681	.2215	.0002	.0017	.1084	.1218	.9182	.8059	.0502	.0060	.0060	.1318	.0506	.9863	.3921	.0100	.0100	.0100	.5780	.2316	.6517
AA3	.5909	.2727	.0000	.0000	.1364	.6498	.2445	.0000	.0007	.1050	.0324	.9947	.8059	.0502	.0060	.0060	.1318	.1384	.9132	.9305	.0174	.0174	.0174	.0174	.1976	.8882
AA4	.5909	.2273	.0000	.0455	.1364	.4452	.4430	.0002	.0042	.1075	.1186	.8361	.8059	.0502	.0060	.0060	.1318	.1258	.9486	.9305	.0174	.0174	.0174	.0174	.1869	.9287
AI1	.0000	1.0000	.0000	.0000	.0000	.0028	.7794	.0154	.0850	.1175	.1183	.9893	.0060	.8059	.0060	.0502	.1318	.1074	.9887	.0174	.9305	.0174	.0174	.0174	.0348	1.0000
AI2	.0000	.8182	.0000	.0455	.1364	.0017	.7193	.0351	.1268	.1171	.0600	.9928	.0060	.8059	.0060	.0502	.1318	.0073	1.0000	.0100	.3921	.0100	.0100	.5780	.2750	.5324
AI3	.0000	.9091	.0000	.0000	.0909	.0028	.7794	.0154	.0850	.1175	.0707	.9954	.0060	.8059	.0060	.0502	.1318	.0546	.9977	.0174	.9305	.0174	.0174	.0174	.0368	.9951
AI4	.0000	.6818	.0000	.0909	.2273	.0017	.7193	.0351	.1268	.1171	.0567	.9767	.0060	.8059	.0060	.0502	.1318	.0724	.9834	.0100	.3921	.0100	.0100	.5780	.2068	.6525
IA1	.0000	.7273	.0000	.0000	.2727	.0040	.7193	.0351	.1186	.1231	.0869	.9523	.0060	.8059	.0060	.0502	.1318	.0757	.9700	.0100	.3921	.0100	.0100	.5780	.2029	.7122
IA2	.0455	.6364	.0000	.0455	.2727	.0040	.7193	.0351	.1186	.1231	.0867	.9454	.0060	.8059	.0060	.0502	.1318	.1002	.9632	.0100	.3921	.0100	.0100	.5780	.1764	.7280
IA3	.0455	.9091	.0455	.0000	.0000	.0011	.7794	.0154	.0913	.1129	.0903	.9795	.0060	.8059	.0060	.0502	.1318	.0820	.9800	.0174	.9305	.0174	.0174	.0174	.0230	.9984
IA4	.0000	.9091	.0000	.0455	.0455	.0011	.7794	.0154	.0913	.1129	.0689	.9959	.0060	.8059	.0060	.0502	.1318	.0603	.9942	.0174	.9305	.0174	.0174	.0174	.0230	.9984
AE1	.0000	.0000	.5000	.0000	.5000	.0000	.0003	.7578	.1369	.1050	.2197	.6646	.0060	.0060	.8059	.0502	.1318	.2153	.7165	.0032	.0032	.2978	.4012	.2947	.2209	.4756
AE2	.0000	.0000	.9545	.0000	.0455	.0000	.0000	.8967	.0000	.1033	.0366	.9977	.0060	.0060	.8059	.0502	.1318	.0802	.9942	.0174	.0174	.9305	.0174	.0174	.0213	.9989
AE3	.0000	.0000	.6364	.1818	.1818	.0000	.0003	.7578	.1369	.1050	.0674	.9857	.0060	.0060	.8059	.0502	.1318	.0986	.9698	.0032	.0032	.2978	.4012	.2947	.1874	.6032
AE4	.0000	.0000	.8636	.0455	.0909	.0000	.0000	.8967	.0000	.1033	.0257	.9984	.0060	.0060	.8059	.0502	.1318	.0319	.9986	.0174	.0174	.9305	.0174	.0174	.0475	.9949
EA1	.0000	.0000	1.0000	.0000	.0000	.0000	.0000	.8967	.0000	.1033	.0653	.9935	.0060	.0060	.8059	.0502	.1318	.1074	.9887	.0174	.0174	.9305	.0174	.0174	.0348	1.0000
EA2	.0000	.0000	.9545	.0455	.0000	.0000	.0000	.8967	.0000	.1033	.0567	.9906	.0060	.0060	.8059	.0502	.1318	.0890	.9877	.0174	.0174	.9305	.0174	.0174	.0213	.9989
EA3	.0000	.0000	.8182	.1818	.0000	.0000	.0002	.7578	.1379	.1041	.0573	.9877	.0060	.0060	.8059	.0502	.1318	.0836	.9646	.0032	.0032	.2978	.4012	.2947	.2849	.4451
EA4	.0000	.0000	.5455	.2727	.1818	.0000	.0002	.7578	.1379	.1041	.1178	.9377	.0060	.0060	.8059	.0502	.1318	.1549	.8938	.0032	.0032	.2978	.4012	.2947	.1346	.7499
AO1	.0000	.0000	.0000	.8636	.1364	.0249	.1083	.0021	.7472	.1175	.0725	.9903	.0060	.0502	.0060	.8059	.1318	.0345	.9985	.0100	.0100	.0100	.3921	.5780	.2890	.5391
AO2	.0000	.0000	.0000	.9091	.0909	.0107	.0699	.0034	.7988	.1171	.0598	.9970	.0060	.0502	.0060	.8059	.1318	.0546	.9977	.0100	.0100	.0100	.3921	.5780	.3178	.4887
AO3	.0000	.0455	.0000	.6364	.3182	.0249	.1083	.0021	.7472	.1175	.1069	.9231	.0060	.0502	.0060	.8059	.1318	.1127	.9387	.0100	.0100	.0100	.3921	.5780	.1604	.7847
AO4	.0000	.0000	.0000	.6818	.3182	.0107	.0699	.0034	.7988	.1171	.1088	.9338	.0060	.0502	.0060	.8059	.1318	.1027	.9442	.0100	.0100	.0100	.3921	.5780	.1742	.7735
OA1	.0000	.0000	.0000	.7727	.2273	.0249	.1000	.0049	.7472	.1231	.0666	.9764	.0060	.0502	.0060	.8059	.1318	.0506	.9863	.0100	.0100	.0100	.3921	.5780	.2316	.6517
OA2	.0000	.0000	.0909	.6818	.2273	.0249	.1000	.0049	.7472	.1231	.0814	.9566	.0060	.0502	.0060	.8059	.1318	.0828	.9731	.0100	.0100	.0100	.3921	.5780	.2068	.6525
OA3	.0000	.0000	.0000	.9545	.0455	.0099	.0731	.0014	.8027	.1129	.0813	.9948	.0060	.0502	.0060	.8059	.1318	.0802	.9942	.0100	.0100	.0100	.3921	.5780	.3465	.4423
OA4	.0000	.0000	.0000	.6364	.3636	.0099	.0731	.0014	.8027	.1129	.1386	.8910	.0060	.0502	.0060	.8059	.1318	.1304	.9068	.0100	.0100	.0100	.3921	.5780	.1456	.8331
II1	.0000	.5000	.0000	.0455	.4545	.0013	.4393	.0139	.0620	.4835	.0316	.9914	.0060	.3976	.0060	.0502	.5401	.0598	.9651	.0100	.3921	.0100	.0100	.5780	.0753	.9495
II2	.0000	.6364	.0455	.0000	.3182	.0013	.4393	.0139	.0620	.4835	.1192	.8774	.0060	.3976	.0060	.0502	.5401	.1486	.8072	.0100	.3921	.0100	.0100	.5780	.1604	.7847
II3	.0000	.3636	.0000	.0000	.6364	.0013	.4393	.0139	.0620	.4835	.0814	.9587	.0060	.3976	.0060	.0502	.5401	.0510	.9882	.0100	.3921	.0100	.0100	.5780	.0300	.9959
II4	.0000	.4091	.0000	.0000	.5909	.0013	.4393	.0139	.0620	.4835	.0574	.9820	.0060	.3976	.0060	.0502	.5401	.0326	.9970	.0100	.3921	.0100	.0100	.5780	.0123	.9999
IE1	.0000	.0000	.3182	.0909	.5909	.0079	.0427	.0855	.3804	.4835	.1740	.6694	.0060	.0060	.3976	.0502	.5401	.0461	.9793	.0032	.0032	.2978	.4012	.2947	.1921	.5607
IE2	.0000	.0000	.2273	.4545	.3182	.0079	.0427	.0855	.3804	.4835	.1047	.8448	.0060	.0060	.3976	.0502	.5401	.2199	.4239	.0032	.0032	.2978	.4012	.2947	.0410	.9746
IE3	.0000	.0000	.3636	.4091	.2273	.0079	.0427	.0855	.3804	.4835	.1707	.5733	.0060	.0060	.3976	.0502	.5401	.2135	.4498	.0032	.0032	.2978	.4012	.2947	.0423	.9701
IE4	.0000	.0455	.2273	.3636	.3636	.0079	.0427	.0855	.3804	.4835	.0835	.9099	.0060	.0060	.3976	.0502	.5401	.1789	.6097	.0032	.0032	.2978	.4012	.2947	.0509	.9517
EI1	.0000	.0000	.1364	.6818	.1818	.0079	.0427	.0855	.3804	.4835	.1931	.6520	.0060	.0060	.3976	.0502	.5401	.3451	-.0472	.0032	.0032	.2978	.4012	.2947	.1533	.8069
EI2	.0000	.0000	.1364	.7273	.1364	.0079	.0427	.0855	.3804	.4835	.2215	.5884	.0060	.0060	.3976	.0502	.5401	.3714	-.1171	.0032	.0032	.2978	.4012	.2947	.1775	.7720
EI3	.0000	.0000	.1818	.5455	.2727	.0079	.0427	.0855	.3804	.4835	.1287	.7897	.0060	.0060	.3976	.0502	.5401	.2696	.2066	.0032	.0032	.2978	.4012	.2947	.0834	.9168
EI4	.0000	.0000	.2273	.4091	.3636	.0079	.0427	.0855	.3804	.4835	.0863	.8955	.0060	.0060	.3976	.0502	.5401	.1944	.5493	.0032	.0032	.2978	.4012	.2947	.0443	.9672
IO1	.0000	.0455	.0000	.4091	.5455	.0094	.0510	.0016	.4544	.4835	.0347	.9893	.0060	.0502	.0060	.3976	.5401	.0071	.9999	.0100	.0100	.0100	.3921	.5780	.0237	.9957
IO2	.0000	.0000	.0000	.5909	.4091	.0094	.0510	.0016	.4544	.4835	.0733	.9603	.0060	.0502	.0060	.3976	.5401	.1069	.9054	.0100	.0100	.0100	.3921	.5780	.1169	.8879
IO3	.0000	.0000	.0000	.3182	.6818	.0094	.0510	.0016	.4544	.4835	.1101	.9196	.0060	.0502	.0060	.3976	.5401	.0761	.9697	.0100	.0100	.0100	.3921	.5780	.0575	.9817
IO4	.0000	.0000	.0000	.4091	.5909	.0094	.0510	.0016	.4544	.4835	.0570	.9794	.0060	.0502	.0060	.3976	.5401	.0326	.9970	.0100	.0100	.0100	.3921	.5780	.0123	.9999
OI1	.0000	.0455	.0000	.4091	.5455	.0094	.0510	.0016	.4544	.4835	.0347	.9893	.0060	.0502	.0060	.3976	.5401	.0071	.9999	.0100	.0100	.0100	.3921	.5780	.0237	.9957
OI2	.0000	.0000	.0000	.3636	.6364	.0094	.0510	.0016	.4544	.4835	.0828	.9536	.0060	.0502	.0060	.3976	.5401	.0510	.9882	.0100	.0100	.0100	.3921	.5780	.0300	.9959
OI3	.0000	.0000	.0000	.5000	.5000	.0094	.0510	.0016	.4544	.4835	.0318	.9962	.0060	.0502	.0060	.3976	.5401	.0542	.9771	.0100	.0100	.0100	.3921	.5780	.0601	.9695
OI4	.0000	.0000	.0455	.3182	.6364	.0094	.0510	.0016	.4544	.4835	.0965	.9221	.0060	.0502	.0060	.3976	.5401	.0628	.9697	.0100	.0100	.0100	.3921	.5780	.0454	.9833
EE1	.0000	.0000	.2727	.0000	.7273	.1041	.0343	.0034	.0466	.8116	.1370	.8957	.0060	.0060	.1780	.0502	.7598	.0503	.9845	.0100	.0100	.3921	.0100	.5780	.0858	.9602
EE2	.0000	.0000	.2727	.0455	.6818	.1041	.0343	.0034	.0466	.8116	.1424	.8871	.0060	.0060	.1780	.0502	.7598	.0551	.9842	.0100	.0100	.3921	.0100	.5780	.0728	.9614
EE3	.0000	.0000	.2273	.0000	.7727	.1041	.0343	.0034	.0466	.8116	.1147	.9289	.0060	.0060	.1780	.0502	.7598	.0322	.9949	.0100	.0100	.3921	.0100	.5780	.1143	.9338
EE4	.0000	.0000	.1818	.0455	.7727	.1041	.0343	.0034	.0466	.8116	.0953	.9508	.0060	.0060	.1780	.0502	.7598	.0074	1.0000	.0100	.0100	.3921	.0100	.5780	.1293	.9022
EO1	.0000	.0455	.1818	.2273	.5455	.0034	.0184	.0006	.1637	.8139	.1481	.9391	.0060	.0502	.0060	.1780	.7598	.1260	.9377	.0100	.0100	.3921	.0100	.5780	.1370	.8214
EO2	.0000	.1364	.0909	.0455	.7273	.0034	.0184	.0006	.1637	.8139	.0934	.9603	.0060	.0502	.0060	.1780	.7598	.0816	.9591	.0100	.0100	.3921	.0100	.5780	.1614	.8030
EO3	.0000	.0000	.0909	.0455	.8636	.0034	.0184	.0006	.1637	.8139	.0707	.9781	.0060	.0502	.0060	.1780	.7598	.0873	.9715	.0100	.0100	.3921	.0100	.5780	.1864	.8376
EO4	.0000	.0000	.1818	.0455	.7727	.0034	.0184	.0006	.1637	.8139	.0989	.9488	.0060	.0502	.0060	.1780	.7598	.1012	.9396	.0100	.0100	.3921	.0100	.5780	.1293	.9022
OE1	.0000	.0000	.1818	.0000	.8182	.0034	.0184	.0006	.1637	.8139	.1096	.9396	.0060	.0502	.0060	.1780	.7598	.1171	.9296	.0100	.0100	.3921	.0100	.5780	.1430	.9046
OE2	.0000	.1364	.1364	.0000	.7273	.0034	.0184	.0006	.1637	.8139	.1155	.9318	.0060	.0502	.0060	.1780	.7598	.1069	.9281	.0100	.0100	.3921	.0100	.5780	.1441	.8473
OE3	.0000	.0909	.1818	.0455	.6818	.0034	.0184	.0006	.1637	.8139	.1179	.9374	.0060	.0502	.0060	.1780	.7598	.1061	.9315	.0100	.0100	.3921	.0100	.5780	.1121	.8951
OE4	.0000	.0455	.0909	.1818	.6818	.0034	.0184	.0006	.1637	.8139	.0731	.9925	.0060	.0502	.0060	.1780	.7598	.0517	.9917	.0100	.0100	.3921	.0100	.5780	.1627	.7785
OO1	.0000	.0000	.0000	.2727	.7273	.0038	.0204	.0006	.1821	.7930	.0509	.9873	.0060	.0502	.0060	.1516	.7861	.0644	.9766	.0100	.0100	.0100	.3921	.5780	.0858	.9602
OO2	.0000	.0909	.0000	.1364	.7727	.0038	.0204	.0006	.1821	.7930	.0387	.9925	.0060	.0502	.0060	.1516	.7861	.0207	.9978	.0100	.0100	.0100	.3921	.5780	.1484	.8613
OO3	.0000	.0909	.0000	.1364	.7727	.0038	.0204	.0006	.1821	.7930	.0387	.9925	.0060	.0502	.0060	.1516	.7861	.0207	.9978	.0100	.0100	.0100	.3921	.5780	.1484	.8613
OO4	.0000	.0455	.0455	.0909	.8182	.0038	.0204	.0006	.1821	.7930	.0482	.9879	.0060	.0502	.0060	.1516	.7861	.0356	.9940	.0100	.0100	.0100	.3921	.5780	.1738	.8306
Mean											.0902	.9696						.0946	.9801						.1251	.9791

**Table 4:** Data from Dickstein׳s [5] Experiment 2 and models’ predictions.

Type	Data (*N*=19)					PRT							PHM							pMM						
	A	I	E	O	N	A	I	E	O	N	RMSD	*r*	A	I	E	O	N	RMSD	*r*	A	I	E	O	N	RMSD	*r*
AA1	.9474	.0263	.0000	.0000	.0263	.8828	.0000	.0000	.0000	.1172	.0512	.9932	.7152	.1101	.0231	.0231	.1285	.1204	.9908	.8996	.0251	.0251	.0251	.0251	.0266	.9995
AA2	.6842	.0526	.0263	.0263	.2105	.6970	.1776	.0004	.0016	.1233	.0702	.9623	.7152	.1101	.0231	.0231	.1285	.0469	.9839	.3521	.0511	.0511	.0511	.4947	.1961	.6364
AA3	.6579	.2895	.0000	.0000	.0526	.6444	.2340	.0001	.0012	.1204	.0396	.9886	.7152	.1101	.0231	.0231	.1285	.0919	.9365	.8996	.0251	.0251	.0251	.0251	.1615	.9059
AA4	.7105	.2105	.0263	.0000	.0526	.4767	.3938	.0004	.0054	.1236	.1371	.8642	.7152	.1101	.0231	.0231	.1285	.0573	.9765	.8996	.0251	.0251	.0251	.0251	.1196	.9612
AI1	.0000	.8947	.0526	.0526	.0000	.0040	.7066	.0352	.1172	.1370	.1083	.9799	.0231	.7152	.0231	.1101	.1285	.1034	.9828	.0251	.8996	.0251	.0251	.0251	.0237	.9977
AI2	.0000	.6316	.0526	.2105	.1053	.0026	.6420	.0657	.1540	.1358	.0297	.9914	.0231	.7152	.0231	.1101	.1285	.0617	.9779	.0511	.3521	.0511	.0511	.4947	.2271	.4126
AI3	.0263	.8947	.0263	.0526	.0000	.0040	.7066	.0352	.1172	.1370	.1086	.9792	.0231	.7152	.0231	.1101	.1285	.1021	.9839	.0251	.8996	.0251	.0251	.0251	.0168	.9989
AI4	.0000	.7632	.0000	.0000	.2368	.0026	.6420	.0657	.1540	.1358	.1029	.9565	.0231	.7152	.0231	.1101	.1285	.0738	.9727	.0511	.3521	.0511	.0511	.4947	.2206	.6681
IA1	.0000	.6316	.0000	.1316	.2368	.0050	.6420	.0657	.1456	.1418	.0523	.9746	.0231	.7152	.0231	.1101	.1285	.0636	.9731	.0511	.3521	.0511	.0511	.4947	.1768	.6672
IA2	.0263	.4474	.0526	.2368	.2368	.0050	.6420	.0657	.1456	.1418	.1057	.9194	.0231	.7152	.0231	.1101	.1285	.1417	.8970	.0511	.3521	.0511	.0511	.4947	.1488	.6352
IA3	.0263	.8421	.0000	.0789	.0526	.0016	.7066	.0352	.1258	.1308	.0755	.9903	.0231	.7152	.0231	.1101	.1285	.0684	.9943	.0251	.8996	.0251	.0251	.0251	.0390	.9967
IA4	.0000	.8947	.0263	.0000	.0789	.0016	.7066	.0352	.1258	.1308	.1039	.9853	.0231	.7152	.0231	.1101	.1285	.0973	.9899	.0251	.8996	.0251	.0251	.0251	.0289	.9966
AE1	.0000	.0526	.5263	.0526	.3684	.0001	.0004	.7628	.1170	.1198	.1579	.8417	.0231	.0231	.7152	.1101	.1285	.1400	.8456	.0149	.0149	.3664	.2813	.3226	.1277	.7935
AE2	.0000	.0000	.9474	.0263	.0263	.0000	.0000	.8828	.0000	.1172	.0512	.9932	.0231	.0231	.7152	.1101	.1285	.1204	.9908	.0251	.0251	.8996	.0251	.0251	.0266	.9995
AE3	.0000	.0263	.7105	.0789	.1842	.0001	.0004	.7628	.1170	.1198	.0424	.9917	.0231	.0231	.7152	.1101	.1285	.0305	.9933	.0149	.0149	.3664	.2813	.3226	.1891	.7053
AE4	.0000	.0526	.8947	.0263	.0263	.0000	.0000	.8828	.0000	.1172	.0487	.9901	.0231	.0231	.7152	.1101	.1285	.1011	.9850	.0251	.0251	.8996	.0251	.0251	.0168	.9989
EA1	.0000	.0263	.9211	.0526	.0000	.0000	.0000	.8828	.0000	.1172	.0611	.9861	.0231	.0231	.7152	.1101	.1285	.1120	.9863	.0251	.0251	.8996	.0251	.0251	.0223	.9985
EA2	.0000	.0000	.9211	.0526	.0263	.0000	.0000	.8828	.0000	.1172	.0500	.9911	.0231	.0231	.7152	.1101	.1285	.1070	.9922	.0251	.0251	.8996	.0251	.0251	.0223	.9985
EA3	.0000	.0000	.7368	.2368	.0263	.0000	.0002	.7628	.1183	.1186	.0682	.9714	.0231	.0231	.7152	.1101	.1285	.0749	.9652	.0149	.0149	.3664	.2813	.3226	.2133	.6685
EA4	.0263	.0526	.5263	.0526	.3421	.0000	.0002	.7628	.1183	.1186	.1507	.8666	.0231	.0231	.7152	.1101	.1285	.1308	.8722	.0149	.0149	.3664	.2813	.3226	.1263	.7757
AO1	.0263	.0263	.0263	.5526	.3684	.0251	.0991	.0049	.7338	.1370	.1357	.8674	.0231	.1101	.0231	.7152	.1285	.1349	.8564	.0511	.0511	.0511	.3521	.4947	.1077	.8729
AO2	.0000	.0789	.0526	.7368	.1316	.0129	.0681	.0070	.7762	.1358	.0281	.9976	.0231	.1101	.0231	.7152	.1285	.0239	.9968	.0511	.0511	.0511	.3521	.4947	.2380	.5141
AO3	.0000	.1579	.0000	.6579	.1842	.0251	.0991	.0049	.7338	.1370	.0492	.9882	.0231	.1101	.0231	.7152	.1285	.0441	.9876	.0511	.0511	.0511	.3521	.4947	.2032	.5766
AO4	.0000	.0526	.0526	.7368	.1579	.0129	.0681	.0070	.7762	.1358	.0301	.9965	.0231	.1101	.0231	.7152	.1285	.0348	.9926	.0511	.0511	.0511	.3521	.4947	.2298	.5567
OA1	.0000	.0526	.0526	.6053	.2895	.0251	.0901	.0092	.7338	.1418	.0919	.9475	.0231	.1101	.0231	.7152	.1285	.0924	.9381	.0511	.0511	.0511	.3521	.4947	.1475	.7611
OA2	.0000	.1316	.0789	.6316	.1579	.0251	.0901	.0092	.7338	.1418	.0599	.9899	.0231	.1101	.0231	.7152	.1285	.0489	.9924	.0511	.0511	.0511	.3521	.4947	.2007	.5324
OA3	.0000	.1842	.0263	.7895	.0000	.0103	.0680	.0034	.7875	.1308	.0791	.9654	.0231	.1101	.0231	.7152	.1285	.0749	.9753	.0511	.0511	.0511	.3521	.4947	.3023	.3121
OA4	.0263	.1316	.0526	.5000	.2895	.0103	.0680	.0034	.7875	.1308	.1514	.9221	.0231	.1101	.0231	.7152	.1285	.1213	.9192	.0511	.0511	.0511	.3521	.4947	.1192	.7871
II1	.0000	.3158	.0526	.0789	.5526	.0019	.3987	.0299	.0819	.4876	.0483	.9724	.0231	.3578	.0231	.1101	.4859	.0415	.9824	.0511	.3521	.0511	.0511	.4947	.0402	.9839
II2	.0263	.5000	.0526	.0526	.3684	.0019	.3987	.0299	.0819	.4876	.0727	.9338	.0231	.3578	.0231	.1101	.4859	.0874	.8973	.0511	.3521	.0511	.0511	.4947	.0877	.8965
II3	.0000	.2895	.0526	.0263	.6316	.0019	.3987	.0299	.0819	.4876	.0852	.9392	.0231	.3578	.0231	.1101	.4859	.0829	.9526	.0511	.3521	.0511	.0511	.4947	.0720	.9717
II4	.0000	.3947	.0000	.0263	.5789	.0019	.3987	.0299	.0819	.4876	.0497	.9906	.0231	.3578	.0231	.1101	.4859	.0602	.9913	.0511	.3521	.0511	.0511	.4947	.0543	.9992
IE1	.0263	.0263	.1316	.1316	.6842	.0078	.0365	.1251	.3430	.4876	.1295	.8574	.0231	.0231	.3578	.1101	.4859	.1349	.8407	.0149	.0149	.3664	.2813	.3226	.2043	.5635
IE2	.0000	.0000	.4474	.3684	.1842	.0078	.0365	.1251	.3430	.4876	.1989	.4218	.0231	.0231	.3578	.1101	.4859	.1826	.5196	.0149	.0149	.3664	.2813	.3226	.0821	.8975
IE3	.0000	.0263	.3684	.2105	.3947	.0078	.0365	.1251	.3430	.4876	.1308	.7282	.0231	.0231	.3578	.1101	.4859	.0617	.9473	.0149	.0149	.3664	.2813	.3226	.0460	.9610
IE4	.0000	.0000	.3158	.3421	.3421	.0078	.0365	.1251	.3430	.4876	.1085	.8142	.0231	.0231	.3578	.1101	.4859	.1244	.7589	.0149	.0149	.3664	.2813	.3226	.0376	.9738
EI1	.0000	.1053	.1316	.6842	.0789	.0078	.0365	.1251	.3430	.4876	.2401	.4092	.0231	.0231	.3578	.1101	.4859	.3328	-.1588	.0149	.0149	.3664	.2813	.3226	.2388	.3584
EI2	.0263	.0263	.1579	.5789	.2105	.0078	.0365	.1251	.3430	.4876	.1637	.6485	.0231	.0231	.3578	.1101	.4859	.2591	.1244	.0149	.0149	.3664	.2813	.3226	.1702	.5739
EI3	.0000	.1316	.1316	.5526	.1842	.0078	.0365	.1251	.3430	.4876	.1704	.5811	.0231	.0231	.3578	.1101	.4859	.2647	.0027	.0149	.0149	.3664	.2813	.3226	.1799	.4542
EI4	.0000	.0000	.2632	.2368	.5000	.0078	.0365	.1251	.3430	.4876	.0798	.9084	.0231	.0231	.3578	.1101	.4859	.0725	.9255	.0149	.0149	.3664	.2813	.3226	.0944	.8648
IO1	.0526	.0000	.0263	.3947	.5263	.0102	.0479	.0036	.4507	.4876	.0430	.9808	.0231	.1101	.0231	.3578	.4859	.0566	.9712	.0511	.0511	.0511	.3521	.4947	.0348	.9959
IO2	.0263	.0789	.0789	.4211	.3947	.0102	.0479	.0036	.4507	.4876	.0573	.9891	.0231	.1101	.0231	.3578	.4859	.0573	.9537	.0511	.0511	.0511	.3521	.4947	.0582	.9518
IO3	.0000	.1842	.0263	.3421	.4474	.0102	.0479	.0036	.4507	.4876	.0808	.9431	.0231	.1101	.0231	.3578	.4859	.0394	.9794	.0511	.0511	.0511	.3521	.4947	.0682	.9317
IO4	.0000	.0789	.0263	.4474	.4474	.0102	.0479	.0036	.4507	.4876	.0253	.9961	.0231	.1101	.0231	.3578	.4859	.0469	.9743	.0511	.0511	.0511	.3521	.4947	.0553	.9632
OI1	.0526	.0789	.0000	.2895	.5789	.0102	.0479	.0036	.4507	.4876	.0862	.9217	.0231	.1101	.0231	.3578	.4859	.0561	.9688	.0511	.0511	.0511	.3521	.4947	.0537	.9723
OI2	.0000	.1316	.0526	.4737	.3421	.0102	.0479	.0036	.4507	.4876	.0790	.9424	.0231	.1101	.0231	.3578	.4859	.0848	.8948	.0511	.0511	.0511	.3521	.4947	.0971	.8614
OI3	.0263	.1579	.0263	.3158	.4737	.0102	.0479	.0036	.4507	.4876	.0791	.9473	.0231	.1101	.0231	.3578	.4859	.0290	.9905	.0511	.0511	.0511	.3521	.4947	.0537	.9590
OI4	.0526	.0789	.0263	.2632	.5789	.0102	.0479	.0036	.4507	.4876	.0967	.8995	.0231	.1101	.0231	.3578	.4859	.0624	.9545	.0511	.0511	.0511	.3521	.4947	.0573	.9625
EE1	.0000	.0263	.3421	.0263	.6053	.0585	.0647	.0191	.1510	.7067	.1644	.7825	.0231	.0231	.1980	.1101	.6457	.0774	.9463	.0511	.0511	.3521	.0511	.4947	.0568	.9927
EE2	.0526	.0526	.1316	.0526	.7105	.0585	.0647	.0191	.1510	.7067	.0671	.9659	.0231	.0231	.1980	.1101	.6457	.0523	.9823	.0511	.0511	.3521	.0511	.4947	.1380	.8524
EE3	.0000	.0000	.1842	.0000	.8158	.0585	.0647	.0191	.1510	.7067	.1180	.9358	.0231	.0231	.1980	.1101	.6457	.0920	.9904	.0511	.0511	.3521	.0511	.4947	.1668	.9040
EE4	.0000	.0526	.2105	.0000	.7368	.0585	.0647	.0191	.1510	.7067	.1131	.9144	.0231	.0231	.1980	.1101	.6457	.0663	.9833	.0511	.0511	.3521	.0511	.4947	.1295	.9197
EO1	.0263	.0789	.0789	.2632	.5526	.0058	.0272	.0020	.2562	.7087	.0818	.9939	.0231	.1101	.0231	.1980	.6457	.0583	.9785	.0511	.0511	.3521	.0511	.4947	.1577	.6590
EO2	.0000	.1579	.0526	.1579	.6316	.0058	.0272	.0020	.2562	.7087	.0840	.9604	.0231	.1101	.0231	.1980	.6457	.0331	.9900	.0511	.0511	.3521	.0511	.4947	.1636	.6981
EO3	.0000	.0000	.0789	.4737	.4474	.0058	.0272	.0020	.2562	.7087	.1564	.8179	.0231	.1101	.0231	.1980	.6457	.1619	.7400	.0511	.0511	.3521	.0511	.4947	.2283	.3632
EO4	.0000	.0526	.0526	.0789	.8158	.0058	.0272	.0020	.2562	.7087	.0960	.9534	.0231	.1101	.0231	.1980	.6457	.0978	.9745	.0511	.0511	.3521	.0511	.4947	.1981	.7883
OE1	.0263	.0000	.1316	.0263	.8158	.0058	.0272	.0020	.2562	.7087	.1282	.9120	.0231	.1101	.0231	.1980	.6457	.1283	.9294	.0511	.0511	.3521	.0511	.4947	.1764	.8639
OE2	.0000	.1053	.0526	.2632	.5789	.0058	.0272	.0020	.2562	.7087	.0715	.9895	.0231	.1101	.0231	.1980	.6457	.0450	.9846	.0511	.0511	.3521	.0511	.4947	.1717	.6304
OE3	.0000	.0789	.1579	.2368	.5263	.0058	.0272	.0020	.2562	.7087	.1101	.9596	.0231	.1101	.0231	.1980	.6457	.0842	.9465	.0511	.0511	.3521	.0511	.4947	.1238	.7757
OE4	.0000	.2632	.1053	.0526	.5789	.0058	.0272	.0020	.2562	.7087	.1579	.8149	.0231	.1101	.0231	.1980	.6457	.1061	.8895	.0511	.0511	.3521	.0511	.4947	.1521	.7111
OO1	.0263	.1316	.0263	.2105	.6053	.0064	.0298	.0022	.2805	.6810	.0663	.9813	.0231	.1101	.0231	.2136	.6300	.0149	.9992	.0511	.0511	.0511	.3521	.4947	.0894	.9092
OO2	.0000	.2895	.1053	.1316	.4737	.0064	.0298	.0022	.2805	.6810	.1693	.7775	.0231	.1101	.0231	.2136	.6300	.1189	.8607	.0511	.0511	.0511	.3521	.4947	.1493	.6493
OO3	.0263	.1053	.0000	.2105	.6579	.0064	.0298	.0022	.2805	.6810	.0480	.9854	.0231	.1101	.0231	.2136	.6300	.0164	.9994	.0511	.0511	.0511	.3521	.4947	.1028	.9135
OO4	.0263	.0789	.0000	.2368	.6579	.0064	.0298	.0022	.2805	.6810	.0324	.9945	.0231	.1101	.0231	.2136	.6300	.0238	.9975	.0511	.0511	.0511	.3521	.4947	.0937	.9374
Mean											.0927	.9611						.0896	.9677						.1213	.9263

**Table 5:** Data from Johnson-Laird and Steedman׳s [4] Experiment 2, 1st Test and models’ predictions.

Type	Data (*N*=20)					PRT							PHM							pMM						
	A	I	E	O	N	A	I	E	O	N	RMSD	*r*	A	I	E	O	N	RMSD	*r*	A	I	E	O	N	RMSD	*r*
AA1	.75	.20	.00	.00	.05	.7498	.0000	.0000	.0000	.2502	.1265	.9038	.7002	.0291	.0031	.0031	.2647	.1247	.8999	.8755	.0311	.0311	.0311	.0311	.0965	.9663
AA2	.25	.25	.00	.00	.50	.5535	.1898	.0002	.0017	.2549	.1765	.5946	.7002	.0291	.0031	.0031	.2647	.2477	.4560	.2923	.0095	.0095	.0095	.6791	.1356	.8727
AA3	.50	.25	.00	.00	.25	.5348	.2128	.0000	.0006	.2518	.0228	.9944	.7002	.0291	.0031	.0031	.2647	.1335	.8891	.8755	.0311	.0311	.0311	.0311	.2185	.8018
AA4	.80	.15	.00	.00	.05	.3601	.3818	.0002	.0040	.2539	.2404	.6195	.7002	.0291	.0031	.0031	.2647	.1189	.9217	.8755	.0311	.0311	.0311	.0311	.0665	.9837
AI1	.00	.90	.00	.00	.10	.0025	.6491	.0135	.0725	.2624	.1377	.9587	.0031	.7002	.0031	.0291	.2647	.1165	.9650	.0311	.8755	.0311	.0311	.0311	.0406	.9939
AI2	.00	.45	.00	.00	.55	.0014	.5956	.0317	.1092	.2621	.1530	.7902	.0031	.7002	.0031	.0291	.2647	.1702	.7853	.0095	.2923	.0095	.0095	.6791	.0914	.9378
AI3	.00	.95	.00	.00	.05	.0025	.6491	.0135	.0725	.2624	.1680	.9414	.0031	.7002	.0031	.0291	.2647	.1479	.9481	.0311	.8755	.0311	.0311	.0311	.0420	.9987
AI4	.00	.65	.00	.00	.35	.0014	.5956	.0317	.1092	.2621	.0687	.9766	.0031	.7002	.0031	.0291	.2647	.0462	.9852	.0095	.2923	.0095	.0095	.6791	.2175	.6582
IA1	.00	.80	.00	.00	.20	.0035	.5956	.0317	.1019	.2674	.1074	.9770	.0031	.7002	.0031	.0291	.2647	.0548	.9921	.0095	.2923	.0095	.0095	.6791	.3122	.4159
IA2	.00	.30	.00	.00	.70	.0035	.5956	.0317	.1019	.2674	.2391	.5519	.0031	.7002	.0031	.0291	.2647	.2648	.5272	.0095	.2923	.0095	.0095	.6791	.0124	1.0000
IA3	.00	.90	.00	.00	.10	.0009	.6491	.0135	.0782	.2583	.1373	.9598	.0031	.7002	.0031	.0291	.2647	.1165	.9650	.0311	.8755	.0311	.0311	.0311	.0406	.9939
IA4	.00	.95	.00	.00	.05	.0009	.6491	.0135	.0782	.2583	.1675	.9430	.0031	.7002	.0031	.0291	.2647	.1479	.9481	.0311	.8755	.0311	.0311	.0311	.0420	.9987
AE1	.00	.00	.70	.10	.20	.0000	.0003	.6289	.1190	.2517	.0402	.9927	.0031	.0031	.7002	.0291	.2647	.0430	.9873	.0061	.0061	.3336	.2958	.3585	.1989	.6477
AE2	.00	.00	.85	.00	.15	.0000	.0000	.7498	.0000	.2502	.0634	.9869	.0031	.0031	.7002	.0291	.2647	.0854	.9802	.0311	.0311	.8755	.0311	.0311	.0595	.9844
AE3	.00	.00	.45	.20	.35	.0000	.0003	.6289	.1190	.2517	.0982	.9183	.0031	.0031	.7002	.0291	.2647	.1408	.8747	.0061	.0061	.3336	.2958	.3585	.0676	.9295
AE4	.00	.00	.75	.00	.25	.0000	.0000	.7498	.0000	.2502	.0001	1.0000	.0031	.0031	.7002	.0291	.2647	.0267	.9988	.0311	.0311	.8755	.0311	.0311	.1154	.9432
EA1	.00	.00	.75	.00	.25	.0000	.0000	.7498	.0000	.2502	.0001	1.0000	.0031	.0031	.7002	.0291	.2647	.0267	.9988	.0311	.0311	.8755	.0311	.0311	.1154	.9432
EA2	.00	.00	1.00	.00	.00	.0000	.0000	.7498	.0000	.2502	.1582	.9431	.0031	.0031	.7002	.0291	.2647	.1793	.9305	.0311	.0311	.8755	.0311	.0311	.0623	1.0000
EA3	.00	.00	.40	.25	.35	.0000	.0001	.6289	.1200	.2510	.1258	.8515	.0031	.0031	.7002	.0291	.2647	.1710	.7864	.0061	.0061	.3336	.2958	.3585	.0365	.9776
EA4	.00	.00	.70	.05	.25	.0000	.0001	.6289	.1200	.2510	.0446	.9927	.0031	.0031	.7002	.0291	.2647	.0116	.9991	.0061	.0061	.3336	.2958	.3585	.2032	.6486
AO1	.00	.00	.00	.55	.45	.0213	.0910	.0019	.6234	.2624	.0993	.9158	.0031	.0291	.0031	.7002	.2647	.1075	.9165	.0095	.0095	.0095	.2923	.6791	.1544	.8189
AO2	.00	.00	.00	.60	.40	.0089	.0577	.0031	.6683	.2621	.0736	.9576	.0031	.0291	.0031	.7002	.2647	.0764	.9589	.0095	.0095	.0095	.2923	.6791	.1859	.7415
AO3	.00	.00	.00	.70	.30	.0213	.0910	.0019	.6234	.2624	.0566	.9907	.0031	.0291	.0031	.7002	.2647	.0206	.9975	.0095	.0095	.0095	.2923	.6791	.2491	.5739
AO4	.00	.00	.00	.60	.40	.0089	.0577	.0031	.6683	.2621	.0736	.9576	.0031	.0291	.0031	.7002	.2647	.0764	.9589	.0095	.0095	.0095	.2923	.6791	.1859	.7415
OA1	.00	.00	.00	.75	.25	.0213	.0835	.0044	.6234	.2674	.0690	.9916	.0031	.0291	.0031	.7002	.2647	.0267	.9988	.0095	.0095	.0095	.2923	.6791	.2807	.4923
OA2	.00	.00	.00	.45	.55	.0213	.0835	.0044	.6234	.2674	.1532	.7967	.0031	.0291	.0031	.7002	.2647	.1702	.7853	.0095	.0095	.0095	.2923	.6791	.0914	.9378
OA3	.00	.15	.00	.60	.25	.0080	.0602	.0013	.6723	.2583	.0518	.9855	.0031	.0291	.0031	.7002	.2647	.0706	.9770	.0095	.0095	.0095	.2923	.6791	.2444	.5029
OA4	.00	.00	.00	.35	.65	.0080	.0602	.0013	.6723	.2583	.2285	.6092	.0031	.0291	.0031	.7002	.2647	.2332	.6150	.0095	.0095	.0095	.2923	.6791	.0298	.9936
II1	.00	.50	.00	.00	.50	.0010	.3345	.0114	.0488	.6042	.0903	.9303	.0031	.3144	.0031	.0291	.6504	.1076	.9076	.0095	.2923	.0095	.0095	.6791	.1228	.8857
II2	.00	.25	.00	.00	.75	.0010	.3345	.0114	.0488	.6042	.0787	.9772	.0031	.3144	.0031	.0291	.6504	.0547	.9893	.0095	.2923	.0095	.0095	.6791	.0377	.9959
II3	.00	.15	.00	.00	.85	.0010	.3345	.0114	.0488	.6042	.1393	.9320	.0031	.3144	.0031	.0291	.6504	.1164	.9537	.0095	.2923	.0095	.0095	.6791	.0997	.9684
II4	.00	.35	.00	.00	.65	.0010	.3345	.0114	.0488	.6042	.0312	.9977	.0031	.3144	.0031	.0291	.6504	.0206	.9974	.0095	.2923	.0095	.0095	.6791	.0298	.9936
IE1	.00	.00	.20	.20	.60	.0060	.0323	.0679	.2896	.6042	.0729	.9466	.0031	.0031	.3144	.0291	.6504	.0947	.9304	.0061	.0061	.3336	.2958	.3585	.1307	.8064
IE2	.00	.00	.45	.15	.40	.0060	.0323	.0679	.2896	.6042	.2041	.5329	.0031	.0031	.3144	.0291	.6504	.1384	.8431	.0061	.0061	.3336	.2958	.3585	.0856	.8983
IE3	.00	.00	.10	.45	.45	.0060	.0323	.0679	.2896	.6042	.1016	.8933	.0031	.0031	.3144	.0291	.6504	.2295	.5208	.0061	.0061	.3336	.2958	.3585	.1317	.7722
IE4	.00	.00	.10	.75	.15	.0060	.0323	.0679	.2896	.6042	.2899	.3614	.0031	.0031	.3144	.0291	.6504	.4040	-.1378	.0061	.0061	.3336	.2958	.3585	.2468	.4858
EI1	.00	.00	.15	.50	.35	.0060	.0323	.0679	.2896	.6042	.1528	.7470	.0031	.0031	.3144	.0291	.6504	.2604	.3561	.0061	.0061	.3336	.2958	.3585	.1229	.7831
EI2	.00	.00	.30	.35	.35	.0060	.0323	.0679	.2896	.6042	.1570	.7184	.0031	.0031	.3144	.0291	.6504	.1967	.6331	.0061	.0061	.3336	.2958	.3585	.0290	.9844
EI3	.00	.00	.20	.35	.45	.0060	.0323	.0679	.2896	.6042	.0959	.9115	.0031	.0031	.3144	.0291	.6504	.1768	.7182	.0061	.0061	.3336	.2958	.3585	.0764	.9076
EI4	.00	.15	.30	.30	.25	.0060	.0323	.0679	.2896	.6042	.1966	.4908	.0031	.0031	.3144	.0291	.6504	.2261	.4564	.0061	.0061	.3336	.2958	.3585	.0821	.8720
IO1	.00	.00	.00	.15	.85	.0073	.0389	.0013	.3483	.6042	.1424	.9234	.0031	.0291	.0031	.3144	.6504	.1164	.9537	.0095	.0095	.0095	.2923	.6791	.0997	.9684
IO2	.00	.00	.00	.25	.75	.0073	.0389	.0013	.3483	.6042	.0806	.9726	.0031	.0291	.0031	.3144	.6504	.0547	.9893	.0095	.0095	.0095	.2923	.6791	.0377	.9959
IO3	.00	.00	.00	.25	.75	.0073	.0389	.0013	.3483	.6042	.0806	.9726	.0031	.0291	.0031	.3144	.6504	.0547	.9893	.0095	.0095	.0095	.2923	.6791	.0377	.9959
IO4	.00	.00	.00	.50	.50	.0073	.0389	.0013	.3483	.6042	.0842	.9400	.0031	.0291	.0031	.3144	.6504	.1076	.9076	.0095	.0095	.0095	.2923	.6791	.1228	.8857
OI1	.00	.00	.00	.40	.60	.0073	.0389	.0013	.3483	.6042	.0292	.9944	.0031	.0291	.0031	.3144	.6504	.0463	.9833	.0095	.0095	.0095	.2923	.6791	.0602	.9736
OI2	.00	.00	.00	.15	.85	.0073	.0389	.0013	.3483	.6042	.1424	.9234	.0031	.0291	.0031	.3144	.6504	.1164	.9537	.0095	.0095	.0095	.2923	.6791	.0997	.9684
OI3	.00	.00	.00	.35	.65	.0073	.0389	.0013	.3483	.6042	.0271	.9983	.0031	.0291	.0031	.3144	.6504	.0206	.9974	.0095	.0095	.0095	.2923	.6791	.0298	.9936
OI4	.00	.40	.00	.00	.60	.0073	.0389	.0013	.3483	.6042	.2244	.5866	.0031	.0291	.0031	.3144	.6504	.2186	.6281	.0095	.0095	.0095	.2923	.6791	.2211	.6341
EE1	.00	.00	.35	.00	.65	.1078	.0424	.0044	.0607	.7846	.1759	.8063	.0031	.0031	.1916	.0291	.7732	.0907	.9537	.0095	.0095	.2923	.0095	.6791	.0298	.9936
EE2	.00	.00	.10	.00	.90	.1078	.0424	.0044	.0607	.7846	.0890	.9780	.0031	.0031	.1916	.0291	.7732	.0712	.9913	.0095	.0095	.2923	.0095	.6791	.1312	.9496
EE3	.00	.00	.15	.05	.80	.1078	.0424	.0044	.0607	.7846	.0836	.9617	.0031	.0031	.1916	.0291	.7732	.0241	.9973	.0095	.0095	.2923	.0095	.6791	.0857	.9651
EE4	.00	.00	.50	.00	.50	.1078	.0424	.0044	.0607	.7846	.2622	.5398	.0031	.0031	.1916	.0291	.7732	.1847	.7814	.0095	.0095	.2923	.0095	.6791	.1228	.8857
EO1	.00	.00	.00	.15	.85	.0039	.0209	.0007	.1872	.7873	.0340	.9983	.0031	.0291	.0031	.1916	.7732	.0412	.9976	.0095	.0095	.2923	.0095	.6791	.1641	.8708
EO2	.00	.00	.05	.15	.80	.0039	.0209	.0007	.1872	.7873	.0298	.9952	.0031	.0291	.0031	.1916	.7732	.0332	.9944	.0095	.0095	.2923	.0095	.6791	.1366	.8946
EO3	.00	.00	.00	.20	.80	.0039	.0209	.0007	.1872	.7873	.0125	.9995	.0031	.0291	.0031	.1916	.7732	.0182	.9994	.0095	.0095	.2923	.0095	.6791	.1653	.8459
EO4	.00	.00	.05	.40	.55	.0039	.0209	.0007	.1872	.7873	.1445	.8865	.0031	.0291	.0031	.1916	.7732	.1388	.8892	.0095	.0095	.2923	.0095	.6791	.2136	.6330
OE1	.00	.00	.05	.20	.75	.0039	.0209	.0007	.1872	.7873	.0298	.9965	.0031	.0291	.0031	.1916	.7732	.0271	.9963	.0095	.0095	.2923	.0095	.6791	.1416	.8693
OE2	.00	.00	.10	.15	.75	.0039	.0209	.0007	.1872	.7873	.0512	.9871	.0031	.0291	.0031	.1916	.7732	.0500	.9861	.0095	.0095	.2923	.0095	.6791	.1113	.9184
OE3	.00	.00	.05	.25	.70	.0039	.0209	.0007	.1872	.7873	.0537	.9898	.0031	.0291	.0031	.1916	.7732	.0486	.9902	.0095	.0095	.2923	.0095	.6791	.1531	.8331
OE4	.00	.00	.00	.20	.80	.0039	.0209	.0007	.1872	.7873	.0125	.9995	.0031	.0291	.0031	.1916	.7732	.0182	.9994	.0095	.0095	.2923	.0095	.6791	.1653	.8459
OO1	.00	.00	.00	.10	.90	.0019	.0103	.0004	.0926	.8947	.0062	.9999	.0031	.0291	.0031	.0835	.8813	.0172	.9992	.0095	.0095	.0095	.2923	.6791	.1312	.9496
OO2	.00	.00	.00	.10	.90	.0019	.0103	.0004	.0926	.8947	.0062	.9999	.0031	.0291	.0031	.0835	.8813	.0172	.9992	.0095	.0095	.0095	.2923	.6791	.1312	.9496
OO3	.00	.00	.00	.05	.95	.0019	.0103	.0004	.0926	.8947	.0316	.9989	.0031	.0291	.0031	.0835	.8813	.0366	.9992	.0095	.0095	.0095	.2923	.6791	.1627	.9297
OO4	.00	.00	.00	.20	.80	.0019	.0103	.0004	.0926	.8947	.0642	.9881	.0031	.0291	.0031	.0835	.8813	.0649	.9850	.0095	.0095	.0095	.2923	.6791	.0684	.9845
Mean											.1030	.9784						.1062	.9713						.1190	.9678

**Table 6:** Data from Johnson-Laird and Steedman׳s [4] Experiment 2, 2nd Test and models’ predictions.

Type	Data (*N*=20)					PRT							PHM							pMM						
	A	I	E	O	N	A	I	E	O	N	RMSD	*r*	A	I	E	O	N	RMSD	*r*	A	I	E	O	N	RMSD	*r*
AA1	.85	.10	.00	.00	.05	.7777	.0000	.0000	.0000	.2223	.0948	.9578	.7306	.0185	.0020	.0020	.2469	.1093	.9466	.8808	.0298	.0298	.0298	.0298	.0401	.9936
AA2	.35	.20	.00	.00	.45	.4303	.3150	.0030	.0132	.2386	.1136	.7917	.7306	.0185	.0020	.0020	.2469	.2093	.6678	.1945	.0066	.0066	.0066	.7858	.1868	.8134
AA3	.20	.65	.00	.00	.15	.4000	.3747	.0000	.0015	.2238	.1557	.7588	.7306	.0185	.0020	.0020	.2469	.3714	-.0145	.8808	.0298	.0298	.0298	.0298	.4158	.0000
AA4	.85	.10	.00	.00	.05	.1363	.6156	.0010	.0204	.2266	.4017	-.0307	.7306	.0185	.0020	.0020	.2469	.1093	.9466	.8808	.0298	.0298	.0298	.0298	.0401	.9936
AI1	.00	.90	.00	.00	.10	.0035	.6981	.0082	.0557	.2345	.1114	.9760	.0020	.7306	.0020	.0185	.2469	.1006	.9744	.0298	.8808	.0298	.0298	.0298	.0399	.9939
AI2	.00	.25	.00	.00	.75	.0011	.5735	.0536	.1380	.2338	.2804	.4018	.0020	.7306	.0020	.0185	.2469	.3113	.4095	.0066	.1945	.0066	.0066	.7858	.0300	.9955
AI3	.00	1.00	.00	.00	.00	.0035	.6981	.0082	.0557	.2345	.1728	.9474	.0020	.7306	.0020	.0185	.2469	.1636	.9438	.0298	.8808	.0298	.0298	.0298	.0596	1.0000
AI4	.00	.60	.00	.00	.40	.0011	.5735	.0536	.1380	.2338	.1002	.9267	.0020	.7306	.0020	.0185	.2469	.0904	.9481	.0066	.1945	.0066	.0066	.7858	.2504	.6051
IA1	.00	.70	.00	.00	.30	.0073	.5735	.0536	.1174	.2481	.0841	.9832	.0020	.7306	.0020	.0185	.2469	.0287	.9949	.0066	.1945	.0066	.0066	.7858	.3136	.4131
IA2	.00	.50	.00	.00	.50	.0073	.5735	.0536	.1174	.2481	.1308	.8456	.0020	.7306	.0020	.0185	.2469	.1534	.8387	.0066	.1945	.0066	.0066	.7858	.1872	.7849
IA3	.00	1.00	.00	.00	.00	.0004	.6981	.0082	.0662	.2272	.1716	.9503	.0020	.7306	.0020	.0185	.2469	.1636	.9438	.0298	.8808	.0298	.0298	.0298	.0596	1.0000
IA4	.00	.95	.00	.00	.05	.0004	.6981	.0082	.0662	.2272	.1409	.9645	.0020	.7306	.0020	.0185	.2469	.1321	.9596	.0298	.8808	.0298	.0298	.0298	.0397	.9987
AE1	.00	.00	.55	.15	.30	.0001	.0018	.5239	.2481	.2261	.0562	.9631	.0020	.0020	.7306	.0185	.2469	.1027	.9561	.0000	.0000	.2801	.4481	.2718	.1803	.5667
AE2	.00	.00	.75	.00	.25	.0000	.0000	.7777	.0000	.2223	.0176	.9988	.0020	.0020	.7306	.0185	.2469	.0121	.9998	.0298	.0298	.8808	.0298	.0298	.1168	.9432
AE3	.00	.00	.40	.35	.25	.0001	.0018	.5239	.2481	.2261	.0725	.9283	.0020	.0020	.7306	.0185	.2469	.2094	.6702	.0000	.0000	.2801	.4481	.2718	.0700	.9183
AE4	.00	.00	.90	.00	.10	.0000	.0000	.7777	.0000	.2223	.0773	.9840	.0020	.0020	.7306	.0185	.2469	.1006	.9744	.0298	.0298	.8808	.0298	.0298	.0399	.9939
EA1	.00	.00	.80	.00	.20	.0000	.0000	.7777	.0000	.2223	.0141	.9993	.0020	.0020	.7306	.0185	.2469	.0384	.9963	.0298	.0298	.8808	.0298	.0298	.0874	.9682
EA2	.00	.00	.75	.15	.10	.0000	.0000	.7777	.0000	.2223	.0874	.9573	.0020	.0020	.7306	.0185	.2469	.0886	.9503	.0298	.0298	.8808	.0298	.0298	.0875	.9784
EA3	.00	.00	.35	.35	.30	.0000	.0004	.5239	.2527	.2231	.0955	.8706	.0020	.0020	.7306	.0185	.2469	.2270	.5899	.0000	.0000	.2801	.4481	.2718	.0553	.9488
EA4	.00	.00	.65	.15	.20	.0000	.0004	.5239	.2527	.2231	.0735	.9634	.0020	.0020	.7306	.0185	.2469	.0721	.9746	.0000	.0000	.2801	.4481	.2718	.2149	.4961
AO1	.00	.00	.00	.55	.45	.0378	.1199	.0014	.6064	.2345	.1144	.8863	.0020	.0185	.0020	.7306	.2469	.1218	.9015	.0066	.0066	.0066	.1945	.7858	.2188	.6991
AO2	.00	.00	.00	.70	.30	.0055	.0440	.0041	.7126	.2338	.0361	.9915	.0020	.0185	.0020	.7306	.2469	.0287	.9949	.0066	.0066	.0066	.1945	.7858	.3136	.4131
AO3	.00	.00	.00	.90	.10	.0378	.1199	.0014	.6064	.2345	.1550	.9601	.0020	.0185	.0020	.7306	.2469	.1006	.9744	.0066	.0066	.0066	.1945	.7858	.4401	.1009
AO4	.00	.00	.00	.75	.25	.0055	.0440	.0041	.7126	.2338	.0270	.9983	.0020	.0185	.0020	.7306	.2469	.0121	.9998	.0066	.0066	.0066	.1945	.7858	.3452	.3234
OA1	.00	.00	.00	.75	.25	.0378	.0985	.0092	.6064	.2481	.0798	.9910	.0020	.0185	.0020	.7306	.2469	.0121	.9998	.0066	.0066	.0066	.1945	.7858	.3452	.3234
OA2	.00	.00	.00	.55	.45	.0378	.0985	.0092	.6064	.2481	.1050	.9052	.0020	.0185	.0020	.7306	.2469	.1218	.9015	.0066	.0066	.0066	.1945	.7858	.2188	.6991
OA3	.00	.00	.00	.75	.25	.0043	.0479	.0006	.7202	.2272	.0273	.9975	.0020	.0185	.0020	.7306	.2469	.0121	.9998	.0066	.0066	.0066	.1945	.7858	.3452	.3234
OA4	.00	.00	.00	.50	.50	.0043	.0479	.0006	.7202	.2272	.1583	.8186	.0020	.0185	.0020	.7306	.2469	.1534	.8387	.0066	.0066	.0066	.1945	.7858	.1872	.7849
II1	.00	.20	.00	.00	.80	.0006	.1825	.0064	.0270	.7835	.0164	.9991	.0020	.1689	.0020	.0185	.8086	.0167	.9986	.0066	.1945	.0066	.0066	.7858	.0085	1.0000
II2	.00	.15	.00	.00	.85	.0006	.1825	.0064	.0270	.7835	.0354	.9985	.0020	.1689	.0020	.0185	.8086	.0220	.9995	.0066	.1945	.0066	.0066	.7858	.0353	.9978
II3	.00	.10	.00	.00	.90	.0006	.1825	.0064	.0270	.7835	.0650	.9932	.0020	.1689	.0020	.0185	.8086	.0519	.9955	.0066	.1945	.0066	.0066	.7858	.0665	.9911
II4	.00	.15	.00	.00	.85	.0006	.1825	.0064	.0270	.7835	.0354	.9985	.0020	.1689	.0020	.0185	.8086	.0220	.9995	.0066	.1945	.0066	.0066	.7858	.0353	.9978
IE1	.00	.00	.15	.45	.40	.0033	.0176	.0376	.1580	.7835	.2215	.6660	.0020	.0020	.1689	.0185	.8086	.2659	.5256	.0000	.0000	.2801	.4481	.2718	.0817	.9054
IE2	.00	.00	.40	.35	.25	.0033	.0176	.0376	.1580	.7835	.3010	.2620	.0020	.0020	.1689	.0185	.8086	.3083	.2879	.0000	.0000	.2801	.4481	.2718	.0700	.9183
IE3	.00	.00	.20	.70	.10	.0033	.0176	.0376	.1580	.7835	.3969	-.0092	.0020	.0020	.1689	.0185	.8086	.4399	-.1788	.0000	.0000	.2801	.4481	.2718	.1410	.8628
IE4	.00	.00	.10	.85	.05	.0033	.0176	.0376	.1580	.7835	.4519	-.0469	.0020	.0020	.1689	.0185	.8086	.5043	-.2499	.0000	.0000	.2801	.4481	.2718	.2205	.7773
EI1	.00	.00	.10	.70	.20	.0033	.0176	.0376	.1580	.7835	.3573	.1836	.0020	.0020	.1689	.0185	.8086	.4098	-.0209	.0000	.0000	.2801	.4481	.2718	.1422	.8592
EI2	.00	.00	.10	.65	.25	.0033	.0176	.0376	.1580	.7835	.3259	.2838	.0020	.0020	.1689	.0185	.8086	.3783	.0822	.0000	.0000	.2801	.4481	.2718	.1214	.8809
EI3	.00	.00	.10	.65	.25	.0033	.0176	.0376	.1580	.7835	.3259	.2838	.0020	.0020	.1689	.0185	.8086	.3783	.0822	.0000	.0000	.2801	.4481	.2718	.1214	.8809
EI4	.00	.00	.25	.40	.35	.0033	.0176	.0376	.1580	.7835	.2416	.5809	.0020	.0020	.1689	.0185	.8086	.2692	.5013	.0000	.0000	.2801	.4481	.2718	.0432	.9691
IO1	.00	.00	.00	.10	.90	.0040	.0212	.0007	.1906	.7835	.0667	.9917	.0020	.0185	.0020	.1689	.8086	.0519	.9955	.0066	.0066	.0066	.1945	.7858	.0665	.9911
IO2	.00	.00	.00	.10	.90	.0040	.0212	.0007	.1906	.7835	.0667	.9917	.0020	.0185	.0020	.1689	.8086	.0519	.9955	.0066	.0066	.0066	.1945	.7858	.0665	.9911
IO3	.00	.00	.00	.05	.95	.0040	.0212	.0007	.1906	.7835	.0979	.9826	.0020	.0185	.0020	.1689	.8086	.0831	.9884	.0066	.0066	.0066	.1945	.7858	.0979	.9816
IO4	.00	.00	.00	.20	.80	.0040	.0212	.0007	.1906	.7835	.0129	.9996	.0020	.0185	.0020	.1689	.8086	.0167	.9986	.0066	.0066	.0066	.1945	.7858	.0085	1.0000
OI1	.00	.00	.00	.20	.80	.0040	.0212	.0007	.1906	.7835	.0129	.9996	.0020	.0185	.0020	.1689	.8086	.0167	.9986	.0066	.0066	.0066	.1945	.7858	.0085	1.0000
OI2	.00	.00	.00	.25	.75	.0040	.0212	.0007	.1906	.7835	.0320	.9946	.0020	.0185	.0020	.1689	.8086	.0455	.9906	.0066	.0066	.0066	.1945	.7858	.0300	.9955
OI3	.00	.00	.00	.05	.95	.0040	.0212	.0007	.1906	.7835	.0979	.9826	.0020	.0185	.0020	.1689	.8086	.0831	.9884	.0066	.0066	.0066	.1945	.7858	.0979	.9816
OI4	.00	.15	.00	.00	.85	.0040	.0212	.0007	.1906	.7835	.1071	.9467	.0020	.0185	.0020	.1689	.8086	.0975	.9555	.0066	.0066	.0066	.1945	.7858	.1119	.9412
EE1	.00	.00	.25	.00	.75	.1069	.0448	.0048	.0655	.7781	.1254	.9073	.0020	.0020	.2174	.0185	.7601	.0174	.9982	.0066	.0066	.1945	.0066	.7858	.0300	.9955
EE2	.00	.00	.10	.00	.90	.1069	.0448	.0048	.0655	.7781	.0913	.9776	.0020	.0020	.2174	.0185	.7601	.0821	.9850	.0066	.0066	.1945	.0066	.7858	.0665	.9911
EE3	.00	.00	.20	.15	.65	.1069	.0448	.0048	.0655	.7781	.1225	.9115	.0020	.0020	.2174	.0185	.7601	.0771	.9774	.0066	.0066	.1945	.0066	.7858	.0885	.9733
EE4	.00	.00	.35	.00	.65	.1069	.0448	.0048	.0655	.7781	.1751	.8046	.0020	.0020	.2174	.0185	.7601	.0775	.9663	.0066	.0066	.1945	.0066	.7858	.0925	.9557
EO1	.00	.00	.00	.25	.75	.0040	.0215	.0008	.1930	.7808	.0306	.9950	.0020	.0185	.0020	.2174	.7601	.0174	.9982	.0066	.0066	.1945	.0066	.7858	.1403	.8887
EO2	.00	.00	.05	.10	.85	.0040	.0215	.0008	.1930	.7808	.0572	.9873	.0020	.0185	.0020	.2174	.7601	.0700	.9809	.0066	.0066	.1945	.0066	.7858	.0822	.9690
EO3	.00	.00	.10	.20	.70	.0040	.0215	.0008	.1930	.7808	.0581	.9878	.0020	.0185	.0020	.2174	.7601	.0526	.9881	.0066	.0066	.1945	.0066	.7858	.1037	.9424
EO4	.00	.00	.00	.20	.80	.0040	.0215	.0008	.1930	.7808	.0134	.9997	.0020	.0185	.0020	.2174	.7601	.0212	.9993	.0066	.0066	.1945	.0066	.7858	.1229	.9196
OE1	.00	.00	.05	.10	.85	.0040	.0215	.0008	.1930	.7808	.0572	.9873	.0020	.0185	.0020	.2174	.7601	.0700	.9809	.0066	.0066	.1945	.0066	.7858	.0822	.9690
OE2	.00	.00	.10	.15	.75	.0040	.0215	.0008	.1930	.7808	.0512	.9863	.0020	.0185	.0020	.2174	.7601	.0540	.9829	.0066	.0066	.1945	.0066	.7858	.0786	.9662
OE3	.00	.00	.05	.20	.75	.0040	.0215	.0008	.1930	.7808	.0279	.9967	.0020	.0185	.0020	.2174	.7601	.0247	.9966	.0066	.0066	.1945	.0066	.7858	.1092	.9323
OE4	.00	.00	.00	.30	.70	.0040	.0215	.0008	.1930	.7808	.0608	.9809	.0020	.0185	.0020	.2174	.7601	.0464	.9882	.0066	.0066	.1945	.0066	.7858	.1621	.8462
OO1	.00	.00	.00	.20	.80	.0000	.0001	.0000	.0009	.9990	.1259	.9684	.0020	.0185	.0020	.0010	.9765	.1192	.9665	.0066	.0066	.0066	.1945	.7858	.0085	1.0000
OO2	.00	.00	.00	.00	1.00	.0000	.0001	.0000	.0009	.9990	.0006	1.0000	.0020	.0185	.0020	.0010	.9765	.0134	.9999	.0066	.0066	.0066	.1945	.7858	.1295	.9705
OO3	.00	.00	.00	.00	1.00	.0000	.0001	.0000	.0009	.9990	.0006	1.0000	.0020	.0185	.0020	.0010	.9765	.0134	.9999	.0066	.0066	.0066	.1945	.7858	.1295	.9705
OO4	.00	.00	.00	.00	1.00	.0000	.0001	.0000	.0009	.9990	.0006	1.0000	.0020	.0185	.0020	.0010	.9765	.0134	.9999	.0066	.0066	.0066	.1945	.7858	.1295	.9705
Mean											.1160	.9815						.1194	.9801						.1259	.9872

**Table 7:** Data from Guyote and Sternberg׳s [2] Experiment 1 and models’ predictions.

Type	Data					PRT							PHM							pMM						
	A	I	E	O	N	A	I	E	O	N	RMSD	*r*	A	I	E	O	N	RMSD	*r*	A	I	E	O	N	RMSD	*r*
AA1	.97	.02	.00	.00	.00	.8724	.0000	.0000	.0000	.1276	.0724	.9882	.7232	.0861	.0124	.0124	.1659	.1365	.9778	.8869	.0283	.0283	.0283	.0283	.0433	.9998
AA2	.47	.04	.00	.00	.47	.5326	.3230	.0008	.0062	.1373	.1974	.5779	.7232	.0861	.0124	.0124	.1659	.1783	.7509	.6214	.0220	.0220	.0220	.3126	.0990	.9106
AA3	.52	.33	.00	.02	.11	.5316	.3386	.0000	.0009	.1289	.0137	.9985	.7232	.0861	.0124	.0124	.1659	.1443	.8485	.8869	.0283	.0283	.0283	.0283	.2159	.8106
AA4	.42	.48	.02	.00	.06	.2512	.6071	.0003	.0098	.1316	.1003	.8946	.7232	.0861	.0124	.0124	.1659	.2274	.5690	.8869	.0283	.0283	.0283	.0283	.2912	.5355
AI1	.07	.85	.00	.04	.02	.0027	.7929	.0073	.0583	.1389	.0668	.9812	.0124	.7232	.0124	.0861	.1659	.0927	.9717	.0283	.8869	.0283	.0283	.0283	.0287	.9975
AI2	.00	.69	.00	.04	.26	.0011	.7025	.0331	.1244	.1388	.0679	.9665	.0124	.7232	.0124	.0861	.1659	.0498	.9826	.0220	.6214	.0220	.0220	.3126	.0418	.9913
AI3	.00	.88	.02	.02	.07	.0027	.7929	.0073	.0583	.1389	.0529	.9947	.0124	.7232	.0124	.0861	.1659	.0876	.9881	.0283	.8869	.0283	.0283	.0283	.0233	.9977
AI4	.04	.54	.04	.02	.33	.0011	.7025	.0331	.1244	.1388	.1228	.8813	.0124	.7232	.0124	.0861	.1659	.1152	.9132	.0220	.6214	.0220	.0220	.3126	.0390	.9948
IA1	.02	.80	.00	.00	.16	.0051	.7025	.0331	.1104	.1489	.0680	.9875	.0124	.7232	.0124	.0861	.1659	.0521	.9934	.0220	.6214	.0220	.0220	.3126	.1060	.9561
IA2	.02	.72	.00	.04	.20	.0051	.7025	.0331	.1104	.1489	.0428	.9885	.0124	.7232	.0124	.0861	.1659	.0265	.9954	.0220	.6214	.0220	.0220	.3126	.0681	.9725
IA3	.00	.97	.00	.00	.02	.0006	.7929	.0073	.0658	.1335	.0986	.9898	.0124	.7232	.0124	.0861	.1659	.1341	.9808	.0283	.8869	.0283	.0283	.0283	.0433	.9998
IA4	.02	.88	.00	.00	.09	.0006	.7929	.0073	.0658	.1335	.0534	.9949	.0124	.7232	.0124	.0861	.1659	.0871	.9892	.0283	.8869	.0283	.0283	.0283	.0332	.9953
AE1	.00	.02	.59	.09	.28	.0000	.0008	.6521	.2173	.1298	.0927	.9229	.0124	.0124	.7232	.0861	.1659	.0787	.9665	.0202	.0202	.2498	.3980	.3118	.2059	.4409
AE2	.00	.00	.88	.04	.07	.0000	.0000	.8724	.0000	.1276	.0315	.9958	.0124	.0124	.7232	.0861	.1659	.0851	.9906	.0283	.0283	.8869	.0283	.0283	.0265	.9970
AE3	.02	.07	.47	.09	.33	.0000	.0008	.6521	.2173	.1298	.1376	.8271	.0124	.0124	.7232	.0861	.1659	.1374	.8918	.0202	.0202	.2498	.3980	.3118	.1710	.4622
AE4	.02	.00	.90	.02	.04	.0000	.0000	.8724	.0000	.1276	.0430	.9930	.0124	.0124	.7232	.0861	.1659	.1017	.9832	.0283	.0283	.8869	.0283	.0283	.0158	.9994
EA1	.02	.00	.90	.04	.02	.0000	.0000	.8724	.0000	.1276	.0535	.9887	.0124	.0124	.7232	.0861	.1659	.1048	.9798	.0283	.0283	.8869	.0283	.0283	.0158	.9994
EA2	.00	.04	.85	.07	.02	.0000	.0000	.8724	.0000	.1276	.0610	.9840	.0124	.0124	.7232	.0861	.1659	.0878	.9768	.0283	.0283	.8869	.0283	.0283	.0287	.9975
EA3	.00	.02	.45	.28	.23	.0000	.0002	.6521	.2194	.1283	.1051	.9282	.0124	.0124	.7232	.0861	.1659	.1527	.8507	.0202	.0202	.2498	.3980	.3118	.1105	.7693
EA4	.02	.01	.52	.28	.16	.0000	.0002	.6521	.2194	.1283	.0673	.9796	.0124	.0124	.7232	.0861	.1659	.1257	.9050	.0202	.0202	.2498	.3980	.3118	.1484	.6436
AO1	.00	.07	.00	.62	.30	.0326	.1236	.0011	.7038	.1389	.0859	.9428	.0124	.0861	.0124	.7232	.1659	.0764	.9611	.0220	.0220	.0220	.6214	.3126	.0262	.9940
AO2	.00	.07	.00	.80	.11	.0071	.0578	.0027	.7935	.1388	.0147	.9990	.0124	.0861	.0124	.7232	.1659	.0438	.9969	.0220	.0220	.0220	.6214	.3126	.1234	.9255
AO3	.00	.07	.02	.57	.33	.0326	.1236	.0011	.7038	.1389	.1084	.9084	.0124	.0861	.0124	.7232	.1659	.1009	.9327	.0220	.0220	.0220	.6214	.3126	.0339	.9923
AO4	.00	.04	.00	.64	.30	.0071	.0578	.0027	.7935	.1388	.0999	.9515	.0124	.0861	.0124	.7232	.1659	.0740	.9618	.0220	.0220	.0220	.6214	.3126	.0189	.9979
OA1	.00	.02	.00	.76	.21	.0326	.1095	.0052	.7038	.1489	.0566	.9868	.0124	.0861	.0124	.7232	.1659	.0400	.9936	.0220	.0220	.0220	.6214	.3126	.0783	.9762
OA2	.00	.02	.02	.61	.33	.0326	.1095	.0052	.7038	.1489	.1009	.9201	.0124	.0861	.0124	.7232	.1659	.0942	.9370	.0220	.0220	.0220	.6214	.3126	.0136	.9986
OA3	.00	.07	.00	.83	.09	.0071	.0650	.0006	.7938	.1335	.0256	.9982	.0124	.0861	.0124	.7232	.1659	.0596	.9941	.0220	.0220	.0220	.6214	.3126	.1388	.9146
IE3	.00	.02	.21	.23	.52	.0111	.0624	.0971	.5405	.2888	.1814	.5494	.0124	.0124	.3776	.0861	.5115	.0991	.8775	.0202	.0202	.2498	.3980	.3118	.1213	.7649
IE4	.00	.02	.11	.38	.47	.0111	.0624	.0971	.5405	.2888	.1102	.8383	.0124	.0124	.3776	.0861	.5115	.1788	.5994	.0202	.0202	.2498	.3980	.3118	.0952	.8728
EI1	.02	.04	.07	.69	.16	.0111	.0624	.0971	.5405	.2888	.0897	.9513	.0124	.0124	.3776	.0861	.5115	.3417	-.1064	.0202	.0202	.2498	.3980	.3118	.1679	.7590
EI2	.00	.04	.07	.73	.14	.0111	.0624	.0971	.5405	.2888	.1090	.9429	.0124	.0124	.3776	.0861	.5115	.3600	-.1241	.0202	.0202	.2498	.3980	.3118	.1859	.7494
EI3	.00	.02	.09	.64	.23	.0111	.0624	.0971	.5405	.2888	.0554	.9858	.0124	.0124	.3776	.0861	.5115	.3062	.0452	.0202	.0202	.2498	.3980	.3118	.1351	.8421
EI4	.02	.02	.14	.47	.35	.0111	.0624	.0971	.5405	.2888	.0499	.9672	.0124	.0124	.3776	.0861	.5115	.2145	.3911	.0202	.0202	.2498	.3980	.3118	.0612	.9459
IO4	.00	.04	.00	.64	.30	.0128	.0722	.0015	.6247	.2888	.0177	.9988	.0124	.0861	.0124	.3776	.5115	.1524	.7913	.0220	.0220	.0220	.6214	.3126	.0189	.9979
EE3	.00	.00	.04	.00	.95	.1262	.0018	.0011	.0018	.8692	.0693	.9869	.0124	.0124	.1188	.0861	.7703	.0962	.9932	.0220	.0220	.6214	.0220	.3126	.3862	.2754
EO3	.00	.07	.00	.23	.69	.0023	.0131	.0003	.1134	.8709	.0996	.9784	.0124	.0861	.0124	.1188	.7703	.0622	.9795	.0220	.0220	.6214	.0220	.3126	.3390	.0781
EO4	.00	.02	.07	.19	.71	.0023	.0131	.0003	.1134	.8709	.0856	.9894	.0124	.0861	.0124	.1188	.7703	.0575	.9820	.0220	.0220	.6214	.0220	.3126	.3133	.2281
OE3	.00	.02	.02	.06	.90	.0023	.0131	.0003	.1134	.8709	.0288	.9972	.0124	.0861	.0124	.1188	.7703	.0705	.9946	.0220	.0220	.6214	.0220	.3126	.3765	.2281
Mean											.0773	.9788						.1219	.9521						.1155	.9693

**Table 8:** Data from Johnson-Laird and Bara׳s [6] Experiment 3 and models’ predictions.

Type	Data (*N*=20)					PRT							PHM							pMM						
	A	I	E	O	N	A	I	E	O	N	RMSD	*r*	A	I	E	O	N	RMSD	*r*	A	I	E	O	N	RMSD	*r*
AA1	.85	.00	.00	.00	.15	.7007	.0000	.0000	.0000	.2993	.0945	.9672	.6638	.0587	.0064	.0064	.2647	.1013	.9761	.8245	.0439	.0439	.0439	.0439	.0595	.9844
AA2	.55	.00	.00	.00	.45	.6983	.0023	.0000	.0000	.2994	.0945	.9398	.6638	.0587	.0064	.0064	.2647	.1008	.9181	.3764	.0335	.0335	.0335	.5230	.0881	.9387
AA3	.65	.00	.00	.00	.35	.6981	.0025	.0000	.0000	.2994	.0313	.9942	.6638	.0587	.0064	.0064	.2647	.0469	.9844	.8245	.0439	.0439	.0439	.0439	.1612	.8566
AA4	.95	.00	.00	.00	.05	.6958	.0048	.0000	.0000	.2994	.1593	.9272	.6638	.0587	.0064	.0064	.2647	.1622	.9430	.8245	.0439	.0439	.0439	.0439	.0657	.9987
AI1	.00	1.00	.00	.00	.00	.0017	.5864	.0186	.0828	.3105	.2344	.8684	.0064	.6638	.0064	.0587	.2647	.1932	.9250	.0439	.8245	.0439	.0439	.0439	.0877	1.0000
AI2	.00	.50	.00	.00	.50	.0017	.5859	.0188	.0831	.3105	.1006	.9118	.0064	.6638	.0064	.0587	.2647	.1309	.8607	.0335	.3764	.0335	.0335	.5230	.0619	.9751
AI3	.00	.65	.00	.00	.35	.0017	.5864	.0186	.0828	.3105	.0506	.9919	.0064	.6638	.0064	.0587	.2647	.0469	.9844	.0439	.8245	.0439	.0439	.0439	.1612	.8566
AI4	.00	.70	.00	.00	.30	.0017	.5859	.0188	.0831	.3105	.0639	.9900	.0064	.6638	.0064	.0587	.2647	.0349	.9957	.0335	.3764	.0335	.0335	.5230	.1777	.7647
IA1	.00	.95	.00	.00	.05	.0017	.5859	.0188	.0831	.3105	.2038	.8919	.0064	.6638	.0064	.0587	.2647	.1622	.9430	.0335	.3764	.0335	.0335	.5230	.3335	.4681
IA2	.00	.75	.00	.00	.25	.0017	.5859	.0188	.0831	.3105	.0870	.9787	.0064	.6638	.0064	.0587	.2647	.0473	.9961	.0335	.3764	.0335	.0335	.5230	.2085	.6990
IA3	.00	.60	.00	.00	.40	.0017	.5864	.0186	.0828	.3104	.0555	.9809	.0064	.6638	.0064	.0587	.2647	.0720	.9592	.0439	.8245	.0439	.0439	.0439	.1913	.7906
IA4	.00	.90	.00	.00	.10	.0017	.5864	.0186	.0828	.3104	.1731	.9161	.0064	.6638	.0064	.0587	.2647	.1315	.9604	.0439	.8245	.0439	.0439	.0439	.0541	.9939
AE1	.00	.00	.90	.00	.10	.0000	.0000	.6994	.0012	.2994	.1265	.9480	.0064	.0064	.6638	.0587	.2647	.1315	.9604	.0000	.0000	.5497	.0526	.3977	.2069	.8282
AE2	.00	.00	.70	.00	.30	.0000	.0000	.7007	.0000	.2993	.0004	1.0000	.0064	.0064	.6638	.0587	.2647	.0349	.9957	.0439	.0439	.8245	.0439	.0439	.1318	.9068
AE3	.00	.00	.60	.00	.40	.0000	.0000	.6994	.0012	.2994	.0633	.9748	.0064	.0064	.6638	.0587	.2647	.0720	.9592	.0000	.0000	.5497	.0526	.3977	.0325	.9957
AE4	.00	.00	.85	.00	.15	.0000	.0000	.7007	.0000	.2993	.0945	.9672	.0064	.0064	.6638	.0587	.2647	.1013	.9761	.0439	.0439	.8245	.0439	.0439	.0595	.9844
EA1	.00	.00	.70	.00	.30	.0000	.0000	.7007	.0000	.2993	.0004	1.0000	.0064	.0064	.6638	.0587	.2647	.0349	.9957	.0439	.0439	.8245	.0439	.0439	.1318	.9068
EA2	.00	.00	.70	.00	.30	.0000	.0000	.7007	.0000	.2993	.0004	1.0000	.0064	.0064	.6638	.0587	.2647	.0349	.9957	.0439	.0439	.8245	.0439	.0439	.1318	.9068
EA3	.00	.00	.60	.00	.40	.0000	.0000	.6994	.0012	.2994	.0633	.9748	.0064	.0064	.6638	.0587	.2647	.0720	.9592	.0000	.0000	.5497	.0526	.3977	.0325	.9957
EA4	.00	.00	.90	.00	.10	.0000	.0000	.6994	.0012	.2994	.1265	.9480	.0064	.0064	.6638	.0587	.2647	.1315	.9604	.0000	.0000	.5497	.0526	.3977	.2069	.8282
AO1	.00	.40	.00	.35	.25	.0127	.0682	.0022	.6065	.3105	.1896	.5933	.0064	.0587	.0064	.6638	.2647	.2075	.5715	.0335	.0335	.0335	.3764	.5230	.2058	.4264
AO2	.00	.20	.00	.20	.60	.0125	.0679	.0022	.6069	.3105	.2311	.4767	.0064	.0587	.0064	.6638	.2647	.2637	.3763	.0335	.0335	.0335	.3764	.5230	.1158	.8548
AO3	.00	.15	.00	.45	.40	.0127	.0682	.0022	.6065	.3105	.0887	.9294	.0064	.0587	.0064	.6638	.2647	.1204	.8851	.0335	.0335	.0335	.3764	.5230	.0853	.9131
AO4	.00	.20	.00	.75	.05	.0125	.0679	.0022	.6069	.3105	.1456	.8605	.0064	.0587	.0064	.6638	.2647	.1213	.9049	.0335	.0335	.0335	.3764	.5230	.2805	.3871
OA1	.00	.05	.00	.70	.25	.0127	.0682	.0022	.6065	.3105	.0508	.9887	.0064	.0587	.0064	.6638	.2647	.0183	.9993	.0335	.0335	.0335	.3764	.5230	.1907	.7033
OA2	.00	.40	.00	.35	.25	.0127	.0682	.0022	.6065	.3105	.1896	.5932	.0064	.0587	.0064	.6638	.2647	.2075	.5715	.0335	.0335	.0335	.3764	.5230	.2058	.4264
OA3	.00	.45	.00	.05	.50	.0125	.0680	.0022	.6069	.3104	.3138	.0615	.0064	.0587	.0064	.6638	.2647	.3421	-.0283	.0335	.0335	.0335	.3764	.5230	.2378	.4041
OA4	.00	.20	.00	.50	.30	.0125	.0680	.0022	.6069	.3104	.0763	.9543	.0064	.0587	.0064	.6638	.2647	.0981	.9379	.0335	.0335	.0335	.3764	.5230	.1378	.7654
II1	.00	.75	.00	.00	.25	.0010	.3540	.0113	.0501	.5835	.2327	.6258	.0064	.3818	.0064	.0587	.5468	.2132	.6866	.0335	.3764	.0335	.0335	.5230	.2085	.6990
II2	.00	.35	.00	.00	.65	.0010	.3540	.0113	.0501	.5835	.0376	.9963	.0064	.3818	.0064	.0587	.5468	.0551	.9876	.0335	.3764	.0335	.0335	.5230	.0635	.9894
II3	.00	.15	.00	.00	.85	.0010	.3540	.0113	.0501	.5835	.1518	.9127	.0064	.3818	.0064	.0587	.5468	.1727	.8755	.0335	.3764	.0335	.0335	.5230	.1798	.8721
II4	.00	.90	.00	.00	.10	.0010	.3540	.0113	.0501	.5835	.3270	.4337	.0064	.3818	.0064	.0587	.5468	.3071	.5054	.0335	.3764	.0335	.0335	.5230	.3021	.5190
IE1	.00	.00	.55	.05	.40	.0064	.0344	.0692	.3065	.5835	.2576	.3438	.0064	.0064	.3818	.0587	.5468	.1000	.9031	.0000	.0000	.5497	.0526	.3977	.0015	1.0000
IE2	.00	.00	.30	.00	.70	.0064	.0344	.0692	.3065	.5835	.1800	.7583	.0064	.0064	.3818	.0587	.5468	.0821	.9679	.0000	.0000	.5497	.0526	.3977	.1769	.7695
IE3	.00	.00	.25	.20	.55	.0064	.0344	.0692	.3065	.5835	.0963	.8987	.0064	.0064	.3818	.0587	.5468	.0865	.9216	.0000	.0000	.5497	.0526	.3977	.1642	.7177
IE4	.00	.00	.55	.20	.25	.0064	.0344	.0692	.3065	.5835	.2665	.2037	.0064	.0064	.3818	.0587	.5468	.1652	.7022	.0000	.0000	.5497	.0526	.3977	.0933	.9140
EI1	.00	.00	.25	.30	.45	.0064	.0344	.0692	.3065	.5835	.1018	.8901	.0064	.0064	.3818	.0587	.5468	.1304	.8111	.0000	.0000	.5497	.0526	.3977	.1754	.6545
EI2	.00	.00	.50	.10	.40	.0064	.0344	.0692	.3065	.5835	.2294	.4289	.0064	.0064	.3818	.0587	.5468	.0864	.9220	.0000	.0000	.5497	.0526	.3977	.0307	.9942
EI3	.00	.00	.20	.10	.70	.0064	.0344	.0692	.3065	.5835	.1221	.8847	.0064	.0064	.3818	.0587	.5468	.1080	.9120	.0000	.0000	.5497	.0526	.3977	.2078	.6473
EI4	.00	.00	.70	.05	.25	.0064	.0344	.0692	.3065	.5835	.3395	.0330	.0064	.0064	.3818	.0587	.5468	.1947	.6969	.0000	.0000	.5497	.0526	.3977	.0943	.9386
IO1	.00	.25	.00	.20	.55	.0076	.0411	.0013	.3664	.5835	.1204	.8589	.0064	.0587	.0064	.3818	.5468	.1181	.8500	.0335	.0335	.0335	.3764	.5230	.1273	.8093
IO2	.00	.10	.00	.30	.60	.0076	.0411	.0013	.3664	.5835	.0405	.9852	.0064	.0587	.0064	.3818	.5468	.0476	.9780	.0335	.0335	.0335	.3764	.5230	.0607	.9651
IO3	.00	.05	.00	.35	.60	.0076	.0411	.0013	.3664	.5835	.0117	.9989	.0064	.0587	.0064	.3818	.5468	.0283	.9949	.0335	.0335	.0335	.3764	.5230	.0428	.9905
IO4	.00	.10	.00	.65	.25	.0076	.0411	.0013	.3664	.5835	.1976	.6588	.0064	.0587	.0064	.3818	.5468	.1799	.7048	.0335	.0335	.0335	.3764	.5230	.1767	.7040
OI1	.00	.15	.00	.65	.20	.0076	.0411	.0013	.3664	.5835	.2188	.5737	.0064	.0587	.0064	.3818	.5468	.2003	.6253	.0335	.0335	.0335	.3764	.5230	.1975	.6182
OI2	.00	.15	.00	.20	.65	.0076	.0411	.0013	.3664	.5835	.0938	.9217	.0064	.0587	.0064	.3818	.5468	.1021	.9044	.0335	.0335	.0335	.3764	.5230	.1123	.8825
OI3	.00	.10	.00	.30	.60	.0076	.0411	.0013	.3664	.5835	.0405	.9852	.0064	.0587	.0064	.3818	.5468	.0476	.9780	.0335	.0335	.0335	.3764	.5230	.0607	.9651
OI4	.00	.30	.00	.40	.30	.0076	.0411	.0013	.3664	.5835	.1724	.6809	.0064	.0587	.0064	.3818	.5468	.1546	.7207	.0335	.0335	.0335	.3764	.5230	.1572	.6718
EE1	.00	.00	.50	.00	.50	.0982	.0330	.0033	.0451	.8204	.2691	.5548	.0064	.0064	.1563	.0587	.7723	.1979	.7407	.0335	.0335	.3764	.0335	.5230	.0619	.9751
EE2	.00	.00	.10	.00	.90	.0982	.0330	.0033	.0451	.8204	.0755	.9815	.0064	.0064	.1563	.0587	.7723	.0678	.9956	.0335	.0335	.3764	.0335	.5230	.2107	.8376
EE3	.00	.00	.10	.00	.90	.0982	.0330	.0033	.0451	.8204	.0755	.9815	.0064	.0064	.1563	.0587	.7723	.0678	.9956	.0335	.0335	.3764	.0335	.5230	.2107	.8376
EE4	.00	.00	.60	.00	.40	.0982	.0330	.0033	.0451	.8204	.3303	.3300	.0064	.0064	.1563	.0587	.7723	.2604	.5500	.0335	.0335	.3764	.0335	.5230	.1170	.8887
EO1	.00	.25	.10	.20	.45	.0032	.0175	.0006	.1561	.8226	.2023	.8572	.0064	.0587	.0064	.1563	.7723	.1739	.8786	.0335	.0335	.3764	.0335	.5230	.1775	.5555
EO2	.00	.30	.15	.00	.55	.0032	.0175	.0006	.1561	.8226	.2004	.7851	.0064	.0587	.0064	.1563	.7723	.1748	.8056	.0335	.0335	.3764	.0335	.5230	.1583	.7112
EO3	.00	.00	.20	.00	.80	.0032	.0175	.0006	.1561	.8226	.1140	.9340	.0064	.0587	.0064	.1563	.7723	.1150	.9286	.0335	.0335	.3764	.0335	.5230	.1491	.9066
EO4	.00	.20	.20	.00	.60	.0032	.0175	.0006	.1561	.8226	.1715	.8567	.0064	.0587	.0064	.1563	.7723	.1494	.8660	.0335	.0335	.3764	.0335	.5230	.1158	.8548
OE1	.00	.00	.30	.00	.70	.0032	.0175	.0006	.1561	.8226	.1609	.8614	.0064	.0587	.0064	.1563	.7723	.1545	.8529	.0335	.0335	.3764	.0335	.5230	.0900	.9681
OE2	.00	.05	.30	.00	.65	.0032	.0175	.0006	.1561	.8226	.1702	.8448	.0064	.0587	.0064	.1563	.7723	.1586	.8392	.0335	.0335	.3764	.0335	.5230	.0700	.9701
OE3	.00	.00	.05	.00	.95	.0032	.0175	.0006	.1561	.8226	.0931	.9781	.0064	.0587	.0064	.1563	.7723	.1108	.9763	.0335	.0335	.3764	.0335	.5230	.2418	.8041
OE4	.00	.45	.25	.20	.10	.0032	.0175	.0006	.1561	.8226	.3933	-.3269	.0064	.0587	.0064	.1563	.7723	.3651	-.2875	.0335	.0335	.3764	.0335	.5230	.2819	-.2006
OO1	.00	.10	.00	.35	.55	.0055	.0299	.0010	.2669	.6966	.0817	.9646	.0064	.0587	.0064	.2900	.6386	.0514	.9814	.0335	.0335	.0335	.3764	.5230	.0402	.9828
OO2	.00	.10	.00	.05	.85	.0055	.0299	.0010	.2669	.6966	.1229	.9340	.0064	.0587	.0064	.2900	.6386	.1443	.9135	.0335	.0335	.0335	.3764	.5230	.2098	.7799
OO3	.00	.00	.00	.20	.80	.0055	.0299	.0010	.2669	.6966	.0567	.9915	.0064	.0587	.0064	.2900	.6386	.0868	.9796	.0335	.0335	.0335	.3764	.5230	.1491	.9066
OO4	.00	.00	.00	.75	.25	.0055	.0299	.0010	.2669	.6966	.2945	.4473	.0064	.0587	.0064	.2900	.6386	.2706	.4999	.0335	.0335	.0335	.3764	.5230	.2085	.6990
Mean											.1431	.9445						.1305	.9340						.1433	.9436

**Table 9:** Data from Bara, Bucciarelli, and Johnson-Laird׳s [7] Experiment with Adults and models’ predictions.

Type	Data (*N*=20)					PRT							PHM							pMM						
	A	I	E	O	N	A	I	E	O	N	RMSD	*r*	A	I	E	O	N	RMSD	*r*	A	I	E	O	N	RMSD	*r*
AA1	.85	.10	.00	.00	.05	.8109	.0000	.0000	.0000	.1891	.0786	.9708	.7153	.0774	.0182	.0182	.1709	.0824	.9840	.8834	.0291	.0291	.0291	.0291	.0407	.9936
AA2	.40	.00	.00	.00	.60	.7781	.0324	.0000	.0000	.1895	.2500	.5979	.7153	.0774	.0182	.0182	.1709	.2409	.5657	.4190	.0271	.0271	.0271	.4997	.0503	.9912
AA3	.45	.15	.00	.00	.40	.7735	.0370	.0000	.0000	.1895	.1799	.8082	.7153	.0774	.0182	.0182	.1709	.1605	.7961	.8834	.0291	.0291	.0291	.0291	.2614	.6498
AA4	.95	.00	.00	.00	.05	.7411	.0689	.0000	.0001	.1899	.1166	.9793	.7153	.0774	.0182	.0182	.1709	.1236	.9861	.8834	.0291	.0291	.0291	.0291	.0385	.9987
AI1	.00	.90	.00	.00	.10	.0022	.6699	.0245	.1003	.2030	.1219	.9802	.0182	.7153	.0182	.0774	.1709	.0957	.9928	.0291	.8834	.0291	.0291	.0291	.0396	.9939
AI2	.00	.40	.00	.00	.60	.0021	.6621	.0273	.1055	.2029	.2183	.6110	.0182	.7153	.0182	.0774	.1709	.2409	.5657	.0271	.4190	.0271	.0271	.4997	.0503	.9912
AI3	.00	.95	.00	.00	.05	.0022	.6699	.0245	.1003	.2030	.1500	.9700	.0182	.7153	.0182	.0774	.1709	.1236	.9861	.0291	.8834	.0291	.0291	.0291	.0385	.9987
AI4	.05	.85	.00	.00	.10	.0021	.6621	.0273	.1055	.2029	.1096	.9704	.0182	.7153	.0182	.0774	.1709	.0781	.9880	.0271	.4190	.0271	.0271	.4997	.2636	.5948
IA1	.00	.95	.00	.00	.05	.0023	.6621	.0273	.1046	.2036	.1537	.9687	.0182	.7153	.0182	.0774	.1709	.1236	.9861	.0271	.4190	.0271	.0271	.4997	.3119	.5570
IA2	.00	.45	.00	.00	.55	.0023	.6621	.0273	.1046	.2036	.1880	.7040	.0182	.7153	.0182	.0774	.1709	.2101	.6631	.0271	.4190	.0271	.0271	.4997	.0337	1.0000
IA3	.00	.85	.00	.00	.15	.0020	.6699	.0245	.1013	.2023	.0960	.9877	.0182	.7153	.0182	.0774	.1709	.0710	.9964	.0291	.8834	.0291	.0291	.0291	.0605	.9844
IA4	.00	1.00	.00	.00	.00	.0020	.6699	.0245	.1013	.2023	.1793	.9583	.0182	.7153	.0182	.0774	.1709	.1529	.9773	.0291	.8834	.0291	.0291	.0291	.0583	1.0000
AE1	.00	.00	.85	.00	.15	.0000	.0000	.7930	.0177	.1893	.0319	.9981	.0182	.0182	.7153	.0774	.1709	.0710	.9964	.0332	.0332	.5362	.1011	.2962	.1626	.9386
AE2	.00	.00	.75	.00	.25	.0000	.0000	.8109	.0000	.1891	.0385	.9947	.0182	.0182	.7153	.0774	.1709	.0531	.9867	.0291	.0291	.8834	.0291	.0291	.1176	.9432
AE3	.05	.00	.75	.05	.15	.0000	.0000	.7930	.0177	.1893	.0373	.9957	.0182	.0182	.7153	.0774	.1709	.0273	.9966	.0332	.0332	.5362	.1011	.2962	.1192	.9362
AE4	.00	.00	.90	.00	.10	.0000	.0000	.8109	.0000	.1891	.0563	.9922	.0182	.0182	.7153	.0774	.1709	.0957	.9928	.0291	.0291	.8834	.0291	.0291	.0396	.9939
EA1	.05	.00	.85	.00	.10	.0000	.0000	.8109	.0000	.1891	.0489	.9892	.0182	.0182	.7153	.0774	.1709	.0781	.9880	.0291	.0291	.8834	.0291	.0291	.0407	.9936
EA2	.00	.00	.65	.00	.35	.0000	.0000	.8109	.0000	.1891	.1018	.9533	.0182	.0182	.7153	.0774	.1709	.0927	.9379	.0291	.0291	.8834	.0291	.0291	.1789	.8566
EA3	.00	.00	.70	.05	.25	.0000	.0000	.7930	.0177	.1893	.0518	.9927	.0182	.0182	.7153	.0774	.1709	.0398	.9888	.0332	.0332	.5362	.1011	.2962	.0822	.9858
EA4	.00	.00	.90	.00	.10	.0000	.0000	.7930	.0177	.1893	.0628	.9920	.0182	.0182	.7153	.0774	.1709	.0957	.9928	.0332	.0332	.5362	.1011	.2962	.1915	.9152
AO1	.00	.30	.05	.45	.20	.0156	.0801	.0029	.6983	.2030	.1500	.8412	.0182	.0774	.0182	.7153	.1709	.1563	.8320	.0271	.0271	.0271	.4190	.4997	.1825	.5591
AO2	.00	.20	.00	.45	.35	.0140	.0756	.0031	.7044	.2029	.1428	.8535	.0182	.0774	.0182	.7153	.1709	.1537	.8235	.0271	.0271	.0271	.4190	.4997	.1046	.8717
AO3	.00	.10	.00	.70	.20	.0156	.0801	.0029	.6983	.2030	.0115	.9990	.0182	.0774	.0182	.7153	.1709	.0212	.9968	.0271	.0271	.0271	.4190	.4997	.1874	.7047
AO4	.00	.25	.00	.70	.05	.0140	.0756	.0031	.7044	.2029	.1040	.9227	.0182	.0774	.0182	.7153	.1709	.0952	.9356	.0271	.0271	.0271	.4190	.4997	.2578	.4402
OA1	.00	.10	.10	.60	.20	.0156	.0792	.0033	.6983	.2036	.0628	.9861	.0182	.0774	.0182	.7153	.1709	.0658	.9870	.0271	.0271	.0271	.4190	.4997	.1637	.7008
OA2	.00	.25	.00	.25	.50	.0156	.0792	.0033	.6983	.2036	.2523	.3968	.0182	.0774	.0182	.7153	.1709	.2666	.3392	.0271	.0271	.0271	.4190	.4997	.1263	.8088
OA3	.05	.10	.00	.60	.25	.0138	.0759	.0028	.7052	.2023	.0552	.9915	.0182	.0774	.0182	.7153	.1709	.0654	.9817	.0271	.0271	.0271	.4190	.4997	.1426	.7803
OA4	.00	.35	.00	.35	.30	.0138	.0759	.0028	.7052	.2023	.2054	.6220	.0182	.0774	.0182	.7153	.1709	.2122	.5943	.0271	.0271	.0271	.4190	.4997	.1734	.6052
II1	.00	.95	.00	.00	.05	.0014	.4182	.0163	.0648	.4994	.3128	.5534	.0182	.4289	.0182	.0774	.4574	.2981	.6137	.0271	.4190	.0271	.0271	.4997	.3119	.5570
II2	.00	.00	.00	.00	1.00	.0014	.4182	.0163	.0648	.4994	.2932	.6999	.0182	.4289	.0182	.0774	.4574	.3115	.6438	.0271	.4190	.0271	.0271	.4997	.2926	.7025
II3	.00	.45	.00	.00	.55	.0014	.4182	.0163	.0648	.4994	.0401	.9952	.0182	.4289	.0182	.0774	.4574	.0560	.9907	.0271	.4190	.0271	.0271	.4997	.0337	1.0000
II4	.00	.85	.00	.00	.15	.0014	.4182	.0163	.0648	.4994	.2502	.6525	.0182	.4289	.0182	.0774	.4574	.2360	.7066	.0271	.4190	.0271	.0271	.4997	.2491	.6564
IE1	.00	.05	.50	.10	.35	.0076	.0401	.0912	.3618	.4994	.2272	.3121	.0182	.0182	.4289	.0774	.4574	.0607	.9528	.0332	.0332	.5362	.1011	.2962	.0334	.9850
IE2	.00	.15	.35	.15	.35	.0076	.0401	.0912	.3618	.4994	.1711	.5118	.0182	.0182	.4289	.0774	.4574	.0902	.9287	.0332	.0332	.5362	.1011	.2962	.1046	.8579
IE3	.00	.10	.25	.20	.45	.0076	.0401	.0912	.3618	.4994	.1072	.8375	.0182	.0182	.4289	.0774	.4574	.1040	.8599	.0332	.0332	.5362	.1011	.2962	.1555	.6187
IE4	.00	.25	.40	.25	.10	.0076	.0401	.0912	.3618	.4994	.2496	-.0977	.0182	.0182	.4289	.0774	.4574	.2061	.2990	.0332	.0332	.5362	.1011	.2962	.1596	.5818
EI1	.00	.20	.40	.15	.25	.0076	.0401	.0912	.3618	.4994	.2136	.1855	.0182	.0182	.4289	.0774	.4574	.1284	.7761	.0332	.0332	.5362	.1011	.2962	.1020	.8737
EI2	.00	.00	.45	.10	.45	.0076	.0401	.0912	.3618	.4994	.2007	.5040	.0182	.0182	.4289	.0774	.4574	.0183	.9966	.0332	.0332	.5362	.1011	.2962	.0816	.9194
EI3	.00	.05	.35	.10	.50	.0076	.0401	.0912	.3618	.4994	.1647	.6385	.0182	.0182	.4289	.0774	.4574	.0445	.9750	.0332	.0332	.5362	.1011	.2962	.1246	.7918
EI4	.00	.00	.90	.05	.05	.0076	.0401	.0912	.3618	.4994	.4370	-.2189	.0182	.0182	.4289	.0774	.4574	.2790	.6069	.0332	.0332	.5362	.1011	.2962	.1989	.8902
IO1	.00	.15	.00	.35	.50	.0093	.0488	.0019	.4407	.4994	.0609	.9644	.0182	.0774	.0182	.4289	.4574	.0528	.9647	.0271	.0271	.0271	.4190	.4997	.0653	.9523
IO2	.00	.05	.00	.10	.85	.0093	.0488	.0019	.4407	.4994	.2187	.7469	.0182	.0774	.0182	.4289	.4574	.2297	.7204	.0271	.0271	.0271	.4190	.4997	.2128	.7682
IO3	.00	.05	.00	.45	.50	.0093	.0488	.0019	.4407	.4994	.0060	.9998	.0182	.0774	.0182	.4289	.4574	.0271	.9993	.0271	.0271	.0271	.4190	.4997	.0243	.9955
IO4	.00	.10	.00	.75	.15	.0093	.0488	.0019	.4407	.4994	.2100	.6745	.0182	.0774	.0182	.4289	.4574	.1994	.7049	.0271	.0271	.0271	.4190	.4997	.2185	.6403
OI1	.00	.25	.10	.40	.25	.0093	.0488	.0019	.4407	.4994	.1510	.7424	.0182	.0774	.0182	.4289	.4574	.1270	.7772	.0271	.0271	.0271	.4190	.4997	.1539	.6940
OI2	.00	.10	.00	.25	.65	.0093	.0488	.0019	.4407	.4994	.1111	.8895	.0182	.0774	.0182	.4289	.4574	.1186	.8743	.0271	.0271	.0271	.4190	.4997	.1077	.8966
OI3	.00	.10	.00	.20	.70	.0093	.0488	.0019	.4407	.4994	.1420	.8387	.0182	.0774	.0182	.4289	.4574	.1499	.8199	.0271	.0271	.0271	.4190	.4997	.1378	.8497
OI4	.00	.40	.00	.25	.35	.0093	.0488	.0019	.4407	.4994	.1908	.5531	.0182	.0774	.0182	.4289	.4574	.1722	.5774	.0271	.0271	.0271	.4190	.4997	.1957	.4982
EE1	.00	.00	.60	.00	.40	.0988	.0502	.0059	.0783	.7667	.3181	.3058	.0182	.0182	.1857	.0774	.7005	.2318	.5881	.0271	.0271	.4190	.0271	.4997	.0948	.9314
EE2	.00	.00	.10	.00	.90	.0988	.0502	.0059	.0783	.7667	.0949	.9775	.0182	.0182	.1857	.0774	.7005	.1037	.9899	.0271	.0271	.4190	.0271	.4997	.2299	.7765
EE3	.00	.00	.20	.00	.80	.0988	.0502	.0059	.0783	.7667	.1069	.9387	.0182	.0182	.1857	.0774	.7005	.0579	.9961	.0271	.0271	.4190	.0271	.4997	.1676	.8581
EE4	.00	.00	.60	.00	.40	.0988	.0502	.0059	.0783	.7667	.3181	.3058	.0182	.0182	.1857	.0774	.7005	.2318	.5881	.0271	.0271	.4190	.0271	.4997	.0948	.9314
EO1	.00	.20	.05	.10	.65	.0043	.0225	.0009	.2029	.7695	.1085	.9409	.0182	.0774	.0182	.1857	.7005	.0725	.9609	.0271	.0271	.4190	.0271	.4997	.1973	.6152
EO2	.00	.10	.25	.00	.65	.0043	.0225	.0009	.2029	.7695	.1572	.8460	.0182	.0774	.0182	.1857	.7005	.1354	.8553	.0271	.0271	.4190	.0271	.4997	.1077	.8966
EO3	.00	.00	.10	.05	.85	.0043	.0225	.0009	.2029	.7695	.0897	.9638	.0182	.0774	.0182	.1857	.7005	.1037	.9648	.0271	.0271	.4190	.0271	.4997	.2128	.7682
EO4	.00	.00	.45	.00	.55	.0043	.0225	.0009	.2029	.7695	.2415	.6147	.0182	.0774	.0182	.1857	.7005	.2236	.6081	.0271	.0271	.4190	.0271	.4997	.0337	1.0000
OE1	.00	.10	.50	.00	.40	.0043	.0225	.0009	.2029	.7695	.2942	.3576	.0182	.0774	.0182	.1857	.7005	.2675	.3591	.0271	.0271	.4190	.0271	.4997	.0682	.9481
OE2	.00	.00	.35	.05	.60	.0043	.0225	.0009	.2029	.7695	.1868	.7740	.0182	.0774	.0182	.1857	.7005	.1703	.7672	.0271	.0271	.4190	.0271	.4997	.0580	.9733
OE3	.00	.10	.15	.20	.55	.0043	.0225	.0009	.2029	.7695	.1236	.9661	.0182	.0774	.0182	.1857	.7005	.0906	.9664	.0271	.0271	.4190	.0271	.4997	.1489	.7308
OE4	.00	.20	.25	.05	.50	.0043	.0225	.0009	.2029	.7695	.1947	.7697	.0182	.0774	.0182	.1857	.7005	.1598	.7917	.0271	.0271	.4190	.0271	.4997	.1093	.8594
OO1	.00	.15	.00	.55	.30	.0071	.0375	.0015	.3385	.6154	.1772	.7008	.0182	.0774	.0182	.4088	.4774	.1071	.8628	.0271	.0271	.0271	.4190	.4997	.1213	.8340
OO2	.00	.05	.00	.20	.75	.0071	.0375	.0015	.3385	.6154	.0866	.9584	.0182	.0774	.0182	.4088	.4774	.1545	.8527	.0271	.0271	.0271	.4190	.4997	.1501	.8564
OO3	.00	.00	.00	.25	.75	.0071	.0375	.0015	.3385	.6154	.0740	.9781	.0182	.0774	.0182	.4088	.4774	.1457	.8889	.0271	.0271	.0271	.4190	.4997	.1367	.8990
OO4	.00	.05	.00	.65	.30	.0071	.0375	.0015	.3385	.6154	.1984	.6778	.0182	.0774	.0182	.4088	.4774	.1350	.8443	.0271	.0271	.0271	.4190	.4997	.1380	.8354
Mean										.1529	.9138						.1343	.9384						.1336	.9609	

**Table 10:** Data from Roberts, Newstead, and Griggs’s (2001) [8] Experiment and models’ predictions.

	Data (N=56)					PRT							PHM							pMM						
Type	]	I	E	O	N	A	I	E	O	N	RMSD	r	A	I	E	O	N	RMSD	r	A	I	E	O	N	RMSD	r

AA1	.8036	.0179	.0357	.0000	.1429	.8411	.0000	.0000	.0000	.1589	.0255	.9989	.7330	.0828	.0264	.0264	.1315	.0450	.9958	.8305	.0424	.0424	.0424	.0424	.0515	.9867
AA2	.6786	.0536	.0179	.0000	.2500	.7854	.0548	.0000	.0001	.1597	.0631	.9861	.7330	.0828	.0264	.0264	.1315	.0610	.9743	.4936	.0590	.0590	.0590	.3294	.0956	.9619
AA3	.7500	.0536	.0357	.0000	.1607	.7774	.0629	.0000	.0001	.1596	.0206	.9987	.7330	.0828	.0264	.0264	.1315	.0236	.9971	.8305	.0424	.0424	.0424	.0424	.0670	.9815
AA4	.8571	.0179	.0179	.0000	.1071	.7230	.1164	.0000	.0002	.1603	.0786	.9867	.7330	.0828	.0264	.0264	.1315	.0648	.9975	.8305	.0424	.0424	.0424	.0424	.0398	.9936
AI1	.0179	.8929	.0357	.0000	.0536	.0024	.6957	.0251	.1032	.1735	.1134	.9750	.0264	.7330	.0264	.0828	.1315	.0879	.9908	.0424	.8305	.0424	.0424	.0424	.0359	.9987
AI2	.0357	.7143	.0357	.0357	.1786	.0022	.6822	.0300	.1122	.1734	.0402	.9893	.0264	.7330	.0264	.0828	.1315	.0315	.9933	.0590	.4936	.0590	.0590	.3294	.1209	.9181
AI3	.0179	.7857	.0179	.0536	.1250	.0024	.6957	.0251	.1032	.1735	.0514	.9933	.0264	.7330	.0264	.0828	.1315	.0276	.9995	.0424	.8305	.0424	.0424	.0424	.0451	.9912
AI4	.0000	.8571	.0179	.0357	.0893	.0022	.6822	.0300	.1122	.1734	.0935	.9871	.0264	.7330	.0264	.0828	.1315	.0635	.9979	.0590	.4936	.0590	.0590	.3294	.1978	.8595
IA1	.0357	.6964	.0179	.0536	.1964	.0026	.6822	.0300	.1106	.1746	.0322	.9923	.0264	.7330	.0264	.0828	.1315	.0362	.9918	.0590	.4936	.0590	.0590	.3294	.1105	.9300
IA2	.0000	.7679	.0179	.0714	.1429	.0026	.6822	.0300	.1106	.1746	.0448	.9970	.0264	.7330	.0264	.0828	.1315	.0212	.9994	.0590	.4936	.0590	.0590	.3294	.1519	.8902
IA3	.0536	.7857	.0357	.0000	.1250	.0020	.6957	.0251	.1049	.1722	.0694	.9789	.0264	.7330	.0264	.0828	.1315	.0458	.9912	.0424	.8305	.0424	.0424	.0424	.0465	.9905
IA4	.0179	.8929	.0000	.0357	.0536	.0020	.6957	.0251	.1049	.1722	.1083	.9814	.0264	.7330	.0264	.0828	.1315	.0832	.9951	.0424	.8305	.0424	.0424	.0424	.0359	.9987
AE1	.0000	.0179	.7679	.0179	.1964	.0000	.0000	.8103	.0304	.1593	.0270	.9977	.0264	.0264	.7330	.0828	.1315	.0456	.9903	.0175	.0175	.5648	.1633	.2368	.1134	.9621
AE2	.0179	.0357	.8393	.0000	.1071	.0000	.0000	.8411	.0000	.1589	.0292	.9960	.0264	.0264	.7330	.0828	.1315	.0615	.9940	.0424	.0424	.8305	.0424	.0424	.0366	.9936
AE3	.0000	.0000	.7321	.0714	.1964	.0000	.0000	.8103	.0304	.1593	.0428	.9965	.0264	.0264	.7330	.0828	.1315	.0339	.9925	.0175	.0175	.5648	.1633	.2368	.0880	.9801
AE4	.0000	.0179	.8571	.0179	.1071	.0000	.0000	.8411	.0000	.1589	.0267	.9968	.0264	.0264	.7330	.0828	.1315	.0648	.9975	.0424	.0424	.8305	.0424	.0424	.0398	.9936
EA1	.0000	.0536	.7679	.0357	.1429	.0000	.0000	.8411	.0000	.1589	.0442	.9975	.0264	.0264	.7330	.0828	.1315	.0316	.9958	.0424	.0424	.8305	.0424	.0424	.0565	.9865
EA2	.0000	.0357	.8214	.0357	.1071	.0000	.0000	.8411	.0000	.1589	.0335	.9954	.0264	.0264	.7330	.0828	.1315	.0478	.9976	.0424	.0424	.8305	.0424	.0424	.0351	.9938
EA3	.0179	.0357	.7857	.0536	.1071	.0000	.0000	.8103	.0305	.1592	.0330	.9955	.0264	.0264	.7330	.0828	.1315	.0296	.9984	.0175	.0175	.5648	.1633	.2368	.1249	.9416
EA4	.0179	.0179	.8214	.0357	.1071	.0000	.0000	.8103	.0305	.1592	.0265	.9964	.0264	.0264	.7330	.0828	.1315	.0464	.9982	.0175	.0175	.5648	.1633	.2368	.1407	.9410
AO1	.0357	.2321	.0179	.5714	.1429	.0169	.0847	.0031	.7218	.1735	.0958	.9560	.0264	.0828	.0264	.7330	.1315	.0987	.9530	.0590	.0590	.0590	.4936	.3294	.1209	.8047
AO2	.0179	.1786	.0536	.6071	.1429	.0141	.0768	.0034	.7324	.1734	.0768	.9814	.0264	.0828	.0264	.7330	.1315	.0720	.9836	.0590	.0590	.0590	.4936	.3294	.1129	.8461
AO3	.0179	.1786	.0357	.5536	.2143	.0169	.0847	.0031	.7218	.1735	.0893	.9773	.0264	.0828	.0264	.7330	.1315	.0984	.9633	.0590	.0590	.0590	.4936	.3294	.0817	.9062
AO4	.0000	.0714	.0357	.6964	.1964	.0141	.0768	.0034	.7324	.1734	.0249	.9974	.0264	.0828	.0264	.7330	.1315	.0360	.9918	.0590	.0590	.0590	.4936	.3294	.1122	.9273
OA1	.0000	.1250	.0357	.5893	.2500	.0169	.0830	.0037	.7218	.1746	.0726	.9803	.0264	.0828	.0264	.7330	.1315	.0863	.9628	.0590	.0590	.0590	.4936	.3294	.0690	.9518
OA2	.0000	.2143	.0357	.5357	.2143	.0169	.0830	.0037	.7218	.1746	.1046	.9522	.0264	.0828	.0264	.7330	.1315	.1130	.9370	.0590	.0590	.0590	.4936	.3294	.0929	.8752
OA3	.0000	.1250	.0714	.6607	.1429	.0137	.0774	.0028	.7339	.1722	.0517	.9904	.0264	.0828	.0264	.7330	.1315	.0444	.9934	.0590	.0590	.0590	.4936	.3294	.1189	.8697
OA4	.0179	.1786	.0000	.6607	.1429	.0137	.0774	.0028	.7339	.1722	.0574	.9833	.0264	.0828	.0264	.7330	.1315	.0553	.9828	.0590	.0590	.0590	.4936	.3294	.1282	.8522
II1	.0000	.6786	.0000	.0357	.2857	.0017	.5265	.0211	.0826	.3680	.0807	.9657	.0264	.5193	.0264	.0828	.3452	.0807	.9763	.0590	.4936	.0590	.0590	.3294	.0934	.9784
II2	.0357	.6429	.0179	.0536	.2500	.0017	.5265	.0211	.0826	.3680	.0768	.9480	.0264	.5193	.0264	.0828	.3452	.0712	.9616	.0590	.4936	.0590	.0590	.3294	.0785	.9650
II3	.0179	.2857	.1250	.0893	.4821	.0017	.5265	.0211	.0826	.3680	.1281	.7916	.0264	.5193	.0264	.0828	.3452	.1289	.7638	.0590	.4936	.0590	.0590	.3294	.1213	.7579
II4	.0000	.6786	.0179	.0000	.3036	.0017	.5265	.0211	.0826	.3680	.0826	.9669	.0264	.5193	.0264	.0828	.3452	.0834	.9769	.0590	.4936	.0590	.0590	.3294	.0933	.9852
IE1	.0000	.0179	.5714	.1429	.2679	.0096	.0503	.1166	.4554	.3680	.2513	.1670	.0264	.0264	.5193	.0828	.3452	.0512	.9699	.0175	.0175	.5648	.1633	.2368	.0186	.9967
IE2	.0000	.0714	.4643	.2857	.1786	.0096	.0503	.1166	.4554	.3680	.1929	.3667	.0264	.0264	.5193	.0828	.3452	.1222	.7888	.0175	.0175	.5648	.1633	.2368	.0796	.9250
IE3	.0179	.0179	.3571	.2857	.3214	.0096	.0503	.1166	.4554	.3680	.1341	.6793	.0264	.0264	.5193	.0828	.3452	.1168	.8104	.0175	.0175	.5648	.1633	.2368	.1142	.8269
IE4	.0000	.0893	.3214	.3929	.1964	.0096	.0503	.1166	.4554	.3680	.1240	.7237	.0264	.0264	.5193	.0828	.3452	.1800	.4856	.0175	.0175	.5648	.1633	.2368	.1543	.6458
EI1	.0000	.0714	.3036	.4107	.2143	.0096	.0503	.1166	.4554	.3680	.1106	.7864	.0264	.0264	.5193	.0828	.3452	.1865	.4545	.0175	.0175	.5648	.1633	.2368	.1632	.6017
EI2	.0000	.0179	.5000	.2857	.1964	.0096	.0503	.1166	.4554	.3680	.2032	.3741	.0264	.0264	.5193	.0828	.3452	.1136	.8268	.0175	.0175	.5648	.1633	.2368	.0650	.9468
EI3	.0000	.0893	.4643	.1250	.3214	.0096	.0503	.1166	.4554	.3680	.2163	.2249	.0264	.0264	.5193	.0828	.3452	.0448	.9833	.0175	.0175	.5648	.1633	.2368	.0695	.9442
EI4	.0000	.0179	.6429	.0357	.3036	.0096	.0503	.1166	.4554	.3680	.3028	.0174	.0264	.0264	.5193	.0828	.3452	.0632	.9840	.0175	.0175	.5648	.1633	.2368	.0737	.9674
IO1	.0000	.2679	.0536	.3036	.3750	.0117	.0615	.0025	.5563	.3680	.1478	.7554	.0264	.0828	.0264	.5193	.3452	.1289	.7608	.0590	.0590	.0590	.4936	.3294	.1306	.6987
IO2	.0357	.2679	.0357	.2679	.3929	.0117	.0615	.0025	.5563	.3680	.1601	.6996	.0264	.0828	.0264	.5193	.3452	.1414	.7022	.0590	.0590	.0590	.4936	.3294	.1412	.6390
IO3	.0000	.0893	.0714	.3393	.5000	.0117	.0615	.0025	.5563	.3680	.1185	.8473	.0264	.0828	.0264	.5193	.3452	.1087	.8435	.0590	.0590	.0590	.4936	.3294	.1072	.8322
IO4	.0000	.1250	.0179	.5179	.3393	.0117	.0615	.0025	.5563	.3680	.0366	.9911	.0264	.0828	.0264	.5193	.3452	.0228	.9935	.0590	.0590	.0590	.4936	.3294	.0452	.9768
OI1	.0179	.1250	.0000	.6071	.2500	.0117	.0615	.0025	.5563	.3680	.0642	.9585	.0264	.0828	.0264	.5193	.3452	.0622	.9626	.0590	.0590	.0590	.4936	.3294	.0758	.9503
OI2	.0000	.2500	.0179	.3214	.4107	.0117	.0615	.0025	.5563	.3680	.1363	.7937	.0264	.0828	.0264	.5193	.3452	.1201	.7967	.0590	.0590	.0590	.4936	.3294	.1248	.7412
OI3	.0000	.2500	.0714	.3214	.3571	.0117	.0615	.0025	.5563	.3680	.1384	.8036	.0264	.0828	.0264	.5193	.3452	.1183	.8096	.0590	.0590	.0590	.4936	.3294	.1188	.7529
OI4	.0000	.2857	.0357	.2857	.3929	.0117	.0615	.0025	.5563	.3680	.1583	.7046	.0264	.0828	.0264	.5193	.3452	.1406	.7094	.0590	.0590	.0590	.4936	.3294	.1433	.6430
EE1	.0357	.0000	.4821	.0000	.4821	.1658	.0883	.0108	.1404	.5947	.2363	.4151	.0264	.0264	.3067	.0828	.5577	.0939	.9136	.0590	.0590	.4936	.0590	.3294	.0787	.9561
EE2	.0179	.0357	.2321	.0000	.7143	.1658	.0883	.0108	.1404	.5947	.1467	.8447	.0264	.0264	.3067	.0828	.5577	.0861	.9701	.0590	.0590	.4936	.0590	.3294	.2108	.6275
EE3	.0179	.0357	.2857	.0000	.6607	.1658	.0883	.0108	.1404	.5947	.1577	.7823	.0264	.0264	.3067	.0828	.5577	.0601	.9860	.0590	.0590	.4936	.0590	.3294	.1782	.7097
EE4	.0000	.0179	.3036	.0000	.6786	.1658	.0883	.0108	.1404	.5947	.1702	.7677	.0264	.0264	.3067	.0828	.5577	.0667	.9913	.0590	.0590	.4936	.0590	.3294	.1826	.7281
EO1	.0000	.0536	.2679	.2857	.3929	.0074	.0390	.0016	.3526	.5994	.1538	.7812	.0264	.0828	.0264	.3067	.5577	.1323	.7698	.0590	.0590	.4936	.0590	.3294	.1482	.6088
EO2	.0000	.0893	.2857	.2143	.4107	.0074	.0390	.0016	.3526	.5994	.1662	.7290	.0264	.0828	.0264	.3067	.5577	.1401	.7358	.0590	.0590	.4936	.0590	.3294	.1252	.7239
EO3	.0000	.0357	.2321	.1786	.5536	.0074	.0390	.0016	.3526	.5994	.1309	.8366	.0264	.0828	.0264	.3067	.5577	.1111	.8494	.0590	.0590	.4936	.0590	.3294	.1655	.6177
EO4	.0357	.1071	.2857	.5536	.0179	.0074	.0390	.0016	.3526	.5994	.3049	.0451	.0264	.0828	.0264	.3067	.5577	.2899	-.0140	.0590	.0590	.4936	.0590	.3294	.2785	-.0663
OE1	.0000	.0179	.2679	.1071	.6071	.0074	.0390	.0016	.3526	.5994	.1623	.7563	.0264	.0828	.0264	.3067	.5577	.1453	.7761	.0590	.0590	.4936	.0590	.3294	.1647	.6893
OE2	.0000	.0000	.2857	.2143	.5000	.0074	.0390	.0016	.3526	.5994	.1492	.7808	.0264	.0828	.0264	.3067	.5577	.1317	.7816	.0590	.0590	.4936	.0590	.3294	.1438	.6966
OE3	.0000	.0179	.2679	.2857	.4286	.0074	.0390	.0016	.3526	.5994	.1450	.8020	.0264	.0828	.0264	.3067	.5577	.1268	.7897	.0590	.0590	.4936	.0590	.3294	.1532	.6108
OE4	.0000	.1071	.1786	.3393	.3750	.0074	.0390	.0016	.3526	.5994	.1316	.8855	.0264	.0828	.0264	.3067	.5577	.1085	.8719	.0590	.0590	.4936	.0590	.3294	.1927	.2994
OO1	.0179	.0893	.0714	.5179	.3036	.0109	.0574	.0023	.5196	.4097	.0585	.9717	.0264	.0828	.0264	.4853	.3791	.0422	.9760	.0590	.0590	.0590	.4936	.3294	.0283	.9886
OO2	.0000	.1429	.0179	.4107	.4286	.0109	.0574	.0023	.5196	.4097	.0630	.9652	.0264	.0828	.0264	.4853	.3791	.0498	.9664	.0590	.0590	.0590	.4936	.3294	.0760	.9143
OO3	.0179	.0357	.0893	.3214	.5357	.0109	.0574	.0023	.5196	.4097	.1124	.8605	.0264	.0828	.0264	.4853	.3791	.1074	.8517	.0590	.0590	.0590	.4936	.3294	.1228	.7963
OO4	.0000	.1071	.0179	.5536	.3214	.0109	.0574	.0023	.5196	.4097	.0486	.9754	.0264	.0828	.0264	.4853	.3791	.0432	.9805	.0590	.0590	.0590	.4936	.3294	.0472	.9826
Mean											.1059	.9520						.0840	.9707						.1059	.9296

**Table 11:** Data from Hattori’s (in press) [1] Experiment 1 and models’ predictions.

	Data (N=88)					PRT							PHM							pMM						
Type	A	I	E	O	N	A	I	E	O	N	RMSD	r	A	I	E	O	N	RMSD	r	A	I	E	O	N	RMSD	r
AA1	.9659	.0227	.0000	.0114	.0000	.8324	.0000	.0000	.0000	.1676	.0965	.9770	.6725	.1141	.0188	.0188	.1757	.1586	.9694	.8322	.0420	.0420	.0420	.0420	.0674	.9998
AA2	.1818	.2500	.0000	.0568	.5114	.4706	.3384	.0015	.0095	.1800	.2016	.3819	.6725	.1141	.0188	.0188	.1757	.2734	.1913	.5602	.0554	.0554	.0554	.2737	.2194	.3301
AA3	.3636	.3068	.0000	.0341	.2955	.4694	.3607	.0000	.0010	.1689	.0790	.9159	.6725	.1141	.0188	.0188	.1757	.1717	.7156	.8322	.0420	.0420	.0420	.0420	.2667	.5399
AA4	.2955	.4091	.0000	.0909	.2045	.1828	.6318	.0005	.0135	.1714	.1178	.8977	.6725	.1141	.0188	.0188	.1757	.2171	.4709	.8322	.0420	.0420	.0420	.0420	.3012	.3297
AI1	.0341	.8750	.0114	.0455	.0341	.0028	.7622	.0059	.0509	.1782	.0831	.9771	.0188	.6725	.0188	.1141	.1757	.1149	.9734	.0420	.8322	.0420	.0420	.0420	.0241	.9995
AI2	.0341	.5568	.0000	.1364	.2727	.0010	.6586	.0363	.1260	.1782	.0661	.9678	.0188	.6725	.0188	.1141	.1757	.0691	.9691	.0554	.5602	.0554	.0554	.2737	.0449	.9750
IA1	.0227	.7159	.0000	.0795	.1818	.0059	.6586	.0363	.1090	.1902	.0341	.9968	.0188	.6725	.0188	.1141	.1757	.0264	.9983	.0554	.5602	.0554	.0554	.2737	.0865	.9711
IA3	.0114	.7955	.0000	.1250	.0682	.0004	.7622	.0059	.0589	.1725	.0575	.9819	.0188	.6725	.0188	.1141	.1757	.0738	.9854	.0420	.8322	.0420	.0420	.0420	.0482	.9890
AE1	.0000	.1023	.4432	.1023	.3523	.0001	.0011	.5869	.2417	.1702	.1292	.8002	.0188	.0188	.6725	.1141	.1757	.1351	.8469	.0568	.0568	.3864	.1761	.3239	.0544	.9587
AE2	.0000	.0227	.8523	.0341	.0909	.0000	.0000	.8324	.0000	.1676	.0399	.9926	.0188	.0188	.6725	.1141	.1757	.0962	.9860	.0420	.0420	.8322	.0420	.0420	.0316	.9958
EA1	.0114	.0227	.8295	.0455	.0909	.0000	.0000	.8324	.0000	.1676	.0415	.9918	.0188	.0188	.6725	.1141	.1757	.0856	.9863	.0420	.0420	.8322	.0420	.0420	.0273	.9963
EA3	.0114	.1136	.3295	.2500	.2955	.0000	.0003	.5869	.2445	.1683	.1382	.8091	.0188	.0188	.6725	.1141	.1757	.1786	.7171	.0568	.0568	.3864	.1761	.3239	.0544	.9164
Mean											.0904	.9595						.1334	.9415						.1022	.9762

**Table 12:** Data from Hattori’s (in press) [1] Experiment 2 and models’ predictions.

	Data (N=50)					PRT							PHM							pMM						
Type	A	I	E	O	N	A	I	E	O	N	RMSD	r	A	I	E	O	N	RMSD	r	A	I	E	O	N	RMSD	r
AA1	1.00	.00	.00	.00	.00	.7878	.0000	.0000	.0000	.2122	.1342	.9630	.5600	.1829	.0213	.0213	.2146	.2341	.9138	.8513	.0372	.0372	.0372	.0372	.0743	1.0000
AA2	.16	.14	.00	.10	.60	.4279	.3286	.0027	.0130	.2277	.2252	.2987	.5600	.1829	.0213	.0213	.2146	.2518	.2257	.4741	.0896	.0896	.0896	.2572	.2130	.3284
AA3	.42	.26	.00	.04	.28	.4117	.3736	.0000	.0012	.2136	.0615	.9379	.5600	.1829	.0213	.0213	.2146	.0783	.9264	.8513	.0372	.0372	.0372	.0372	.2433	.6985
AA4	.30	.44	.00	.08	.18	.1374	.6270	.0008	.0186	.2162	.1153	.8844	.5600	.1829	.0213	.0213	.2146	.1666	.5762	.8513	.0372	.0372	.0372	.0372	.3130	.3196
AI1	.04	.88	.00	.04	.04	.0031	.7166	.0064	.0505	.2233	.1112	.9593	.0213	.5600	.0213	.1829	.2146	.1756	.9234	.0372	.8513	.0372	.0372	.0372	.0211	.9990
AI2	.02	.54	.02	.14	.28	.0009	.5972	.0461	.1328	.2230	.0390	.9853	.0213	.5600	.0213	.1829	.2146	.0361	.9831	.0896	.4741	.0896	.0896	.2572	.0585	.9742
AI3	.14	.70	.00	.08	.08	.0031	.7166	.0064	.0505	.2233	.0900	.9431	.0213	.5600	.0213	.1829	.2146	.1121	.9067	.0372	.8513	.0372	.0372	.0372	.0878	.9845
AI4	.04	.60	.00	.16	.20	.0009	.5972	.0461	.1328	.2230	.0314	.9891	.0213	.5600	.0213	.1829	.2146	.0251	.9956	.0896	.4741	.0896	.0896	.2572	.0832	.9516
IA1	.06	.60	.00	.12	.22	.0070	.5972	.0461	.1127	.2370	.0325	.9884	.0213	.5600	.0213	.1829	.2146	.0388	.9850	.0896	.4741	.0896	.0896	.2572	.0736	.9741
IA3	.04	.80	.00	.08	.08	.0004	.7166	.0064	.0598	.2168	.0744	.9721	.0213	.5600	.0213	.1829	.2146	.1320	.9452	.0372	.8513	.0372	.0372	.0372	.0392	.9951
IA4	.06	.66	.00	.10	.18	.0004	.7166	.0064	.0598	.2168	.0442	.9930	.0213	.5600	.0213	.1829	.2146	.0633	.9746	.0372	.8513	.0372	.0372	.0372	.1121	.9692
AE1	.00	.04	.48	.14	.34	.0001	.0016	.5296	.2531	.2156	.0803	.9121	.0213	.0213	.5600	.1829	.2146	.0704	.9341	.0411	.0411	.4027	.1816	.3335	.0434	.9874
AE2	.02	.00	.94	.00	.04	.0000	.0000	.7878	.0000	.2122	.1032	.9717	.0213	.0213	.5600	.1829	.2146	.2044	.9197	.0372	.0372	.8513	.0372	.0372	.0468	.9992
AE3	.04	.06	.34	.18	.38	.0001	.0016	.5296	.2531	.2156	.1211	.7883	.0213	.0213	.5600	.1829	.2146	.1246	.7776	.0411	.0411	.4027	.1816	.3335	.0359	.9706
AE4	.00	.04	.88	.00	.08	.0000	.0000	.7878	.0000	.2122	.0743	.9798	.0213	.0213	.5600	.1829	.2146	.1759	.9247	.0372	.0372	.8513	.0372	.0372	.0329	.9962
EA1	.00	.04	.94	.02	.00	.0000	.0000	.7878	.0000	.2122	.1185	.9566	.0213	.0213	.5600	.1829	.2146	.2087	.9073	.0372	.0372	.8513	.0372	.0372	.0468	.9992
EA2	.02	.02	.86	.00	.10	.0000	.0000	.7878	.0000	.2122	.0610	.9851	.0213	.0213	.5600	.1829	.2146	.1653	.9300	.0372	.0372	.8513	.0372	.0372	.0346	.9946
EA3	.02	.04	.34	.34	.26	.0000	.0003	.5296	.2571	.2129	.0970	.8833	.0213	.0213	.5600	.1829	.2146	.1229	.7841	.0411	.0411	.4027	.1816	.3335	.0835	.8351
EA4	.02	.08	.42	.16	.32	.0000	.0003	.5296	.2571	.2129	.0890	.9018	.0213	.0213	.5600	.1829	.2146	.0833	.9209	.0411	.0411	.4027	.1816	.3335	.0241	.9869
AO1	.00	.24	.04	.56	.16	.0380	.1229	.0011	.6146	.2233	.0688	.9516	.0213	.1829	.0213	.5600	.2146	.0376	.9821	.0896	.0896	.0896	.4741	.2572	.0999	.8721
AO2	.00	.28	.08	.48	.16	.0052	.0444	.0034	.7240	.2230	.1581	.8516	.0213	.1829	.0213	.5600	.2146	.0674	.9441	.0896	.0896	.0896	.4741	.2572	.1038	.7935
AO3	.02	.48	.02	.24	.24	.0380	.1229	.0011	.6146	.2233	.2319	.3220	.0213	.1829	.0213	.5600	.2146	.1956	.4422	.0896	.0896	.0896	.4741	.2572	.2084	.1702
AO4	.00	.34	.02	.38	.26	.0052	.0444	.0034	.7240	.2230	.2037	.6759	.0213	.1829	.0213	.5600	.2146	.1092	.8326	.0896	.0896	.0896	.4741	.2572	.1299	.6534
OA1	.00	.20	.00	.58	.22	.0380	.1024	.0079	.6146	.2370	.0500	.9744	.0213	.1829	.0213	.5600	.2146	.0180	.9988	.0896	.0896	.0896	.4741	.2572	.0904	.9297
OA2	.00	.36	.04	.22	.38	.0380	.1024	.0079	.6146	.2370	.2214	.3577	.0213	.1829	.0213	.5600	.2146	.1871	.4606	.0896	.0896	.0896	.4741	.2572	.1807	.3171
OA3	.00	.40	.00	.50	.10	.0045	.0502	.0005	.7281	.2168	.1939	.7121	.0213	.1829	.0213	.5600	.2146	.1138	.8451	.0896	.0896	.0896	.4741	.2572	.1660	.6199
OA4	.00	.42	.10	.22	.26	.0045	.0502	.0005	.7281	.2168	.2852	.1910	.0213	.1829	.0213	.5600	.2146	.1900	.4110	.0896	.0896	.0896	.4741	.2572	.1907	.1635
II3	.00	.42	.02	.12	.44	.0013	.4589	.0126	.0607	.4666	.0340	.9925	.0213	.3773	.0213	.1829	.3973	.0401	.9871	.0896	.4741	.0896	.0896	.2572	.1001	.9925
Mean											.1125	.9309						.1224	.9228						.1049	.9857

**Table 13:** Data from Chater and Oaksford’s (1999) [3] Experiment 1 and models’ predictions.

	Data (N=20)					PRT							PHM						
Type	A	M	F	O	N	A	M	F	O	N	RMSD	r	A	M	F	O	N	RMSD	r
AA1	.85	.00	.00	.10	.05	.8787	.0000	.0000	.0000	.1213	.0564	.9869	.6252	.0306	.0306	.0306	.2831	.1494	.9109
AA2	.60	.05	.10	.20	.20	.8150	.0535	.0102	.0000	.1213	.1417	.9534	.6252	.0306	.0306	.0306	.2831	.0910	.9363
AA3	.65	.00	.05	.15	.20	.7008	.1548	.0230	.0000	.1213	.1058	.9123	.6252	.0306	.0306	.0306	.2831	.0680	.9583
AA4	.55	.15	.10	.15	.25	.6391	.2046	.0349	.0000	.1213	.1041	.9389	.6252	.0306	.0306	.0306	.2831	.0896	.9829
AM1	.05	.85	.00	.40	.00	.0000	.7099	.1687	.0000	.1213	.2123	.7881	.0306	.6252	.0306	.2791	.0345	.1163	.9974
AM2	.05	.60	.05	.35	.20	.0000	.6925	.1862	.0000	.1213	.1779	.7486	.0306	.6252	.0306	.2791	.0345	.0822	.9629
AM3	.10	.60	.10	.40	.10	.0000	.7099	.1687	.0000	.1213	.1935	.7186	.0306	.6252	.0306	.2791	.0345	.0764	.9845
AM4	.05	.70	.10	.35	.05	.0000	.6925	.1862	.0000	.1213	.1659	.8059	.0306	.6252	.0306	.2791	.0345	.0567	.9966
MA1	.05	.65	.10	.40	.05	.0000	.6925	.1862	.0000	.1213	.1881	.7342	.0306	.6252	.0306	.2791	.0345	.0643	.9856
MA2	.10	.50	.10	.35	.15	.0000	.6925	.1862	.0000	.1213	.1886	.7000	.0306	.6252	.0306	.2791	.0345	.0935	.9773
MA3	.05	.65	.15	.50	.05	.0000	.7099	.1687	.0000	.1213	.2287	.6508	.0306	.6252	.0306	.2791	.0345	.1134	.9462
MA4	.20	.55	.15	.30	.05	.0000	.7099	.1687	.0000	.1213	.1795	.7659	.0306	.6252	.0306	.2791	.0345	.0993	.9571
AF1	.05	.00	.85	.40	.00	.0000	.0000	.8787	.0000	.1213	.1887	.8594	.0306	.0306	.6252	.2791	.0345	.1163	.9974
AF2	.05	.00	.85	.25	.10	.0000	.0000	.8787	.0000	.1213	.1151	.9541	.0306	.0306	.6252	.2791	.0345	.1068	.9809
AF3	.05	.00	.80	.35	.05	.0000	.0000	.8787	.0000	.1213	.1651	.8883	.0306	.0306	.6252	.2791	.0345	.0862	.9983
AF4	.05	.00	.75	.25	.15	.0000	.0000	.8787	.0000	.1213	.1284	.9486	.0306	.0306	.6252	.2791	.0345	.0788	.9735
FA1	.10	.00	.80	.30	.05	.0000	.0000	.8787	.0000	.1213	.1492	.9138	.0306	.0306	.6252	.2791	.0345	.0860	.9906
FA2	.05	.05	.70	.30	.15	.0000	.0000	.8787	.0000	.1213	.1598	.9212	.0306	.0306	.6252	.2791	.0345	.0634	.9874
FA3	.10	.05	.60	.45	.00	.0000	.0000	.8787	.0000	.1213	.2479	.6998	.0306	.0306	.6252	.2791	.0345	.0851	.9499
FA4	.05	.05	.65	.45	.05	.0000	.0000	.8787	.0000	.1213	.2302	.7551	.0306	.0306	.6252	.2791	.0345	.0785	.9723
AO1	.05	.25	.10	.90	.05	.0546	.0668	.0977	.6596	.1213	.1389	.9608	.0306	.1283	.1283	.6252	.0876	.1363	.9852
AO2	.05	.15	.10	.75	.10	.0451	.0545	.0994	.6797	.1213	.0539	.9874	.0306	.1283	.1283	.6252	.0876	.0590	.9962
AO3	.05	.10	.10	.80	.15	.0546	.0668	.0977	.6596	.1213	.0658	.9988	.0306	.1283	.1283	.6252	.0876	.0854	.9892
AO4	.05	.10	.10	.80	.15	.0451	.0545	.0994	.6797	.1213	.0590	.9981	.0306	.1283	.1283	.6252	.0876	.0854	.9892
OA1	.10	.20	.10	.85	.10	.0546	.0582	.1062	.6596	.1213	.1085	.9769	.0306	.1283	.1283	.6252	.0876	.1109	.9884
OA2	.05	.15	.20	.65	.10	.0546	.0582	.1062	.6596	.1213	.0596	.9758	.0306	.1283	.1283	.6252	.0876	.0368	.9952
OA3	.05	.10	.15	.85	.10	.0306	.0542	.0792	.7147	.1213	.0725	.9940	.0306	.1283	.1283	.6252	.0876	.1023	.9957
OA4	.10	.25	.30	.55	.10	.0306	.0542	.0792	.7147	.1213	.1546	.8654	.0306	.1283	.1283	.6252	.0876	.1048	.9317
MM1	.00	.65	.15	.35	.15	.0000	.6177	.2183	.0000	.1639	.1603	.7827	.0306	.6252	.0306	.2791	.0345	.0827	.9698
MM2	.00	.45	.20	.30	.25	.0000	.6177	.2183	.0000	.1639	.1587	.7407	.0306	.6252	.0306	.2791	.0345	.1464	.8198
MM3	.00	.50	.05	.50	.10	.0000	.6177	.2183	.0000	.1639	.2434	.4213	.0306	.6252	.0306	.2791	.0345	.1184	.8748
MM4	.00	.50	.15	.35	.15	.0000	.6177	.2183	.0000	.1639	.1681	.6880	.0306	.6252	.0306	.2791	.0345	.0992	.9319
MF1	.00	.00	.75	.25	.10	.0000	.0000	.8361	.0000	.1639	.1217	.9314	.0306	.0306	.6252	.2791	.0345	.0672	.9855
MF2	.00	.10	.60	.35	.15	.0000	.0000	.8361	.0000	.1639	.1941	.8284	.0306	.0306	.6252	.2791	.0345	.0704	.9704
MF3	.00	.10	.55	.50	.10	.0000	.0000	.8361	.0000	.1639	.2630	.6118	.0306	.0306	.6252	.2791	.0345	.1136	.9025
MF4	.00	.05	.70	.30	.10	.0000	.0000	.8361	.0000	.1639	.1517	.8948	.0306	.0306	.6252	.2791	.0345	.0482	.9925
FM1	.00	.10	.60	.50	.05	.0000	.0000	.8361	.0000	.1639	.2564	.6435	.0306	.0306	.6252	.2791	.0345	.1053	.9283
FM2	.00	.20	.50	.40	.10	.0000	.0000	.8361	.0000	.1639	.2518	.6465	.0306	.0306	.6252	.2791	.0345	.1133	.8963
FM3	.00	.10	.70	.30	.10	.0000	.0000	.8361	.0000	.1639	.1566	.8928	.0306	.0306	.6252	.2791	.0345	.0567	.9885
FM4	.00	.15	.65	.30	.10	.0000	.0000	.8361	.0000	.1639	.1739	.8700	.0306	.0306	.6252	.2791	.0345	.0641	.9767
MO1	.00	.10	.25	.80	.10	.0450	.0548	.0942	.6420	.1639	.1071	.9596	.0306	.1283	.1283	.6252	.0876	.0972	.9843
MO2	.00	.10	.20	.75	.15	.0450	.0548	.0942	.6420	.1639	.0736	.9803	.0306	.1283	.1283	.6252	.0876	.0726	.9878
MO3	.00	.30	.15	.65	.15	.0450	.0548	.0942	.6420	.1639	.1145	.8936	.0306	.1283	.1283	.6252	.0876	.0841	.9524
MO4	.00	.15	.20	.75	.15	.0450	.0548	.0942	.6420	.1639	.0826	.9727	.0306	.1283	.1283	.6252	.0876	.0721	.9925
OM1	.00	.20	.20	.60	.20	.0450	.0548	.0942	.6420	.1639	.0865	.9429	.0306	.1283	.1283	.6252	.0876	.0700	.9656
OM2	.00	.20	.25	.70	.15	.0450	.0548	.0942	.6420	.1639	.1009	.9388	.0306	.1283	.1283	.6252	.0876	.0779	.9795
OM3	.00	.25	.30	.75	.10	.0450	.0548	.0942	.6420	.1639	.1402	.8954	.0306	.1283	.1283	.6252	.0876	.1104	.9642
OM4	.00	.25	.15	.70	.15	.0450	.0548	.0942	.6420	.1639	.0968	.9379	.0306	.1283	.1283	.6252	.0876	.0717	.9796
FF1	.00	.05	.55	.35	.20	.0000	.0000	.7676	.0000	.2324	.1862	.7956	.0306	.0306	.6252	.2791	.0345	.0887	.9365
FF2	.00	.10	.55	.30	.20	.0000	.0000	.7676	.0000	.2324	.1723	.8497	.0306	.0306	.6252	.2791	.0345	.0886	.9436
FF3	.00	.10	.50	.35	.20	.0000	.0000	.7676	.0000	.2324	.2026	.7568	.0306	.0306	.6252	.2791	.0345	.1038	.9180
FF4	.00	.05	.60	.30	.20	.0000	.0000	.7676	.0000	.2324	.1560	.8715	.0306	.0306	.6252	.2791	.0345	.0772	.9531
FO1	.00	.05	.40	.60	.20	.0413	.0503	.0865	.5895	.2324	.1422	.8112	.0306	.1283	.0306	.6252	.1853	.1699	.7328
FO2	.00	.00	.55	.65	.10	.0413	.0503	.0865	.5895	.2324	.2192	.6667	.0306	.1283	.0306	.6252	.1853	.2429	.5844
FO3	.00	.05	.60	.60	.10	.0413	.0503	.0865	.5895	.2324	.2379	.5760	.0306	.1283	.0306	.6252	.1853	.2605	.4957
FO4	.00	.00	.55	.60	.10	.0413	.0503	.0865	.5895	.2324	.2176	.6299	.0306	.1283	.0306	.6252	.1853	.2430	.5419
OF1	.00	.05	.45	.40	.15	.0413	.0503	.0865	.5895	.2324	.1879	.5410	.0306	.1283	.0306	.6252	.1853	.2168	.4366
OF2	.00	.00	.45	.55	.15	.0413	.0503	.0865	.5895	.2324	.1701	.7095	.0306	.1283	.0306	.6252	.1853	.2001	.6141
OF3	.00	.00	.50	.55	.15	.0413	.0503	.0865	.5895	.2324	.1916	.6562	.0306	.1283	.0306	.6252	.1853	.2212	.5558
OF4	.00	.05	.65	.40	.15	.0413	.0503	.0865	.5895	.2324	.2690	.3166	.0306	.1283	.0306	.6252	.1853	.2976	.2021
OO1	.00	.10	.10	.80	.10	.0466	.0567	.0975	.6644	.1348	.0688	.9932	.0306	.1283	.0306	.6252	.1853	.0942	.9743
OO2	.00	.10	.20	.70	.15	.0466	.0567	.0975	.6644	.1348	.0567	.9807	.0306	.1283	.0306	.6252	.1853	.0864	.9442
OO3	.00	.15	.15	.60	.25	.0466	.0567	.0975	.6644	.1348	.0788	.9552	.0306	.1283	.0306	.6252	.1853	.0640	.9682
OO4	.00	.05	.20	.70	.20	.0466	.0567	.0975	.6644	.1348	.0604	.9780	.0306	.1283	.0306	.6252	.1853	.0912	.9392
Mean											.1525	.8994						.1064	.9653

**Table 14:** Data from Chater and Oaksford’s (1999) [3] Experiment 2 and models’ predictions.

	Data (N=20)					PRT							PHM						
Type	M	F	I	E	N	M	F	I	E	N	RMSD	r	M	F	I	E	N	RMSD	r
MM1	.45	.10	.20	.15	.20	.3982	.2798	.0000	.0000	.3220	.1499	.5091	.3525	.0996	.1706	.0996	.2778	.0616	.8773
MM2	.50	.15	.20	.05	.25	.3982	.2798	.0000	.0000	.3220	.1224	.7266	.3525	.0996	.1706	.0996	.2778	.0754	.9244
MM3	.35	.10	.45	.05	.15	.3982	.2798	.0000	.0000	.3220	.2321	-.0338	.3525	.0996	.1706	.0996	.2778	.1392	.4768
MM4	.50	.05	.30	.05	.20	.3982	.2798	.0000	.0000	.3220	.1847	.4055	.3525	.0996	.1706	.0996	.2778	.0995	.8597
MF1	.15	.45	.10	.10	.30	.0000	.6780	.0000	.0000	.3220	.1378	.9884	.0996	.3525	.1706	.0996	.2778	.0592	.9339
MF2	.20	.35	.20	.10	.25	.0000	.6780	.0000	.0000	.3220	.2014	.8931	.0996	.3525	.1706	.0996	.2778	.0484	.9030
MF3	.15	.30	.20	.10	.30	.0000	.6780	.0000	.0000	.3220	.2078	.8346	.0996	.3525	.1706	.0996	.2778	.0365	.9491
MF4	.15	.45	.15	.05	.35	.0000	.6780	.0000	.0000	.3220	.1416	.9480	.0996	.3525	.1706	.0996	.2778	.0635	.9676
FM1	.00	.70	.10	.10	.20	.0000	.6780	.0000	.0000	.3220	.0841	.9535	.0996	.3525	.1706	.0996	.2778	.1684	.8778
FM2	.00	.75	.05	.15	.20	.0000	.6780	.0000	.0000	.3220	.0949	.9442	.0996	.3525	.1706	.0996	.2778	.1955	.8387
FM3	.00	.45	.20	.15	.30	.0000	.6780	.0000	.0000	.3220	.1516	.8967	.0996	.3525	.1706	.0996	.2778	.0683	.9415
FM4	.00	.65	.05	.15	.25	.0000	.6780	.0000	.0000	.3220	.0787	.9652	.0996	.3525	.1706	.0996	.2778	.1525	.8783
MI1	.10	.00	.50	.15	.30	.0903	.0783	.3631	.1463	.3220	.0714	.9527	.1225	.1225	.3525	.0996	.3031	.0892	.9142
MI2	.20	.15	.40	.05	.30	.0903	.0783	.3631	.1463	.3220	.0752	.8178	.1225	.1225	.3525	.0996	.3031	.0479	.9346
MI3	.10	.10	.45	.10	.30	.0903	.0783	.3631	.1463	.3220	.0464	.9561	.1225	.1225	.3525	.0996	.3031	.0459	.9790
MI4	.15	.10	.45	.10	.30	.0903	.0783	.3631	.1463	.3220	.0533	.9339	.1225	.1225	.3525	.0996	.3031	.0464	.9715
IM1	.10	.25	.30	.10	.30	.0903	.0783	.3631	.1463	.3220	.0850	.7089	.1225	.1225	.3525	.0996	.3031	.0625	.8141
IM2	.00	.20	.45	.05	.30	.0903	.0783	.3631	.1463	.3220	.0897	.8462	.1225	.1225	.3525	.0996	.3031	.0812	.9087
IM3	.05	.15	.50	.05	.35	.0903	.0783	.3631	.1463	.3220	.0843	.9223	.1225	.1225	.3525	.0996	.3031	.0805	.9758
IM4	.00	.25	.45	.10	.30	.0903	.0783	.3631	.1463	.3220	.0978	.7928	.1225	.1225	.3525	.0996	.3031	.0903	.8448
ME1	.15	.10	.10	.35	.40	.1238	.1659	.0000	.3883	.3220	.0672	.8887	.0996	.0996	.0996	.3525	.3489	.0321	.9811
ME2	.05	.35	.10	.25	.35	.1238	.1659	.0000	.3883	.3220	.1177	.6202	.0996	.0996	.0996	.3525	.3489	.1230	.5206
ME3	.10	.20	.15	.30	.35	.1238	.1659	.0000	.3883	.3220	.0810	.8464	.0996	.0996	.0996	.3525	.3489	.0555	.9229
ME4	.15	.15	.05	.35	.40	.1238	.1659	.0000	.3883	.3220	.0469	.9527	.0996	.0996	.0996	.3525	.3489	.0451	.9528
EM1	.05	.35	.20	.35	.15	.1238	.1659	.0000	.3883	.3220	.1486	.3498	.0996	.0996	.0996	.3525	.3489	.1515	.2150
EM2	.05	.50	.05	.30	.20	.1238	.1659	.0000	.3883	.3220	.1687	.4242	.0996	.0996	.0996	.3525	.3489	.1950	.1466
EM3	.05	.20	.15	.35	.35	.1238	.1659	.0000	.3883	.3220	.0792	.8356	.0996	.0996	.0996	.3525	.3489	.0549	.9101
EM4	.05	.35	.10	.25	.35	.1238	.1659	.0000	.3883	.3220	.1177	.6202	.0996	.0996	.0996	.3525	.3489	.1230	.5206
FF1	.05	.45	.10	.10	.30	.0000	.5975	.0000	.0000	.4025	.1047	.9900	.0996	.3525	.1706	.0996	.2778	.0591	.9719
FF2	.00	.35	.20	.25	.35	.0000	.5975	.0000	.0000	.4025	.1825	.7375	.0996	.3525	.1706	.0996	.2778	.0879	.7674
FF3	.10	.35	.10	.20	.35	.0000	.5975	.0000	.0000	.4025	.1575	.9170	.0996	.3525	.1706	.0996	.2778	.0637	.8438
FF4	.00	.55	.10	.15	.30	.0000	.5975	.0000	.0000	.4025	.0951	.9493	.0996	.3525	.1706	.0996	.2778	.1067	.9293
FI1	.00	.30	.35	.15	.30	.0796	.0690	.3200	.1289	.4025	.1196	.6037	.1225	.1225	.3525	.0996	.3031	.0991	.6741
FI2	.05	.40	.15	.20	.35	.0796	.0690	.3200	.1289	.4025	.1715	.1858	.1225	.1225	.3525	.0996	.3031	.1647	.0581
FI3	.05	.35	.25	.20	.30	.0796	.0690	.3200	.1289	.4025	.1416	.3530	.1225	.1225	.3525	.0996	.3031	.1246	.3292
FI4	.00	.55	.15	.05	.35	.0796	.0690	.3200	.1289	.4025	.2347	.0960	.1225	.1225	.3525	.0996	.3031	.2207	.1045
IF1	.15	.35	.25	.10	.30	.0796	.0690	.3200	.1289	.4025	.1415	.3136	.1225	.1225	.3525	.0996	.3031	.1123	.4121
IF2	.05	.40	.25	.20	.30	.0796	.0690	.3200	.1289	.4025	.1618	.2306	.1225	.1225	.3525	.0996	.3031	.1434	.2294
IF3	.00	.40	.30	.05	.35	.0796	.0690	.3200	.1289	.4025	.1583	.4601	.1225	.1225	.3525	.0996	.3031	.1410	.5305
IF4	.00	.25	.45	.05	.30	.0796	.0690	.3200	.1289	.4025	.1206	.6983	.1225	.1225	.3525	.0996	.3031	.0930	.8578
FE1	.00	.20	.15	.30	.45	.1715	.1486	.0000	.2774	.4025	.1071	.7327	.0996	.0996	.0996	.3525	.3489	.0843	.8389
FE2	.05	.15	.10	.40	.40	.1715	.1486	.0000	.2774	.4025	.0892	.8193	.0996	.0996	.0996	.3525	.3489	.0444	.9776
FE3	.05	.10	.15	.30	.45	.1715	.1486	.0000	.2774	.4025	.0921	.7907	.0996	.0996	.0996	.3525	.3489	.0600	.9179
FE4	.05	.10	.15	.25	.50	.1715	.1486	.0000	.2774	.4025	.0999	.7841	.0996	.0996	.0996	.3525	.3489	.0876	.8407
EF1	.15	.05	.15	.25	.45	.1715	.1486	.0000	.2774	.4025	.0845	.8071	.0996	.0996	.0996	.3525	.3489	.0752	.8384
EF2	.10	.15	.25	.25	.35	.1715	.1486	.0000	.2774	.4025	.1193	.5061	.0996	.0996	.0996	.3525	.3489	.0845	.7459
EF3	.05	.10	.20	.35	.35	.1715	.1486	.0000	.2774	.4025	.1141	.6160	.0996	.0996	.0996	.3525	.3489	.0501	.9211
EF4	.10	.15	.15	.30	.35	.1715	.1486	.0000	.2774	.4025	.0786	.8212	.0996	.0996	.0996	.3525	.3489	.0396	.9669
II1	.00	.10	.50	.05	.35	.0867	.0752	.3484	.1404	.3494	.0886	.9343	.1225	.1225	.3525	.0996	.3031	.0916	.9780
II2	.05	.05	.55	.05	.30	.0867	.0752	.3484	.1404	.3494	.1032	.9028	.1225	.1225	.3525	.0996	.3031	.1020	.9639
II3	.05	.15	.45	.10	.35	.0867	.0752	.3484	.1404	.3494	.0615	.9348	.1225	.1225	.3525	.0996	.3031	.0595	.9736
II4	.00	.05	.60	.05	.30	.0867	.0752	.3484	.1404	.3494	.1281	.8934	.1225	.1225	.3525	.0996	.3031	.1296	.9499
IE1	.10	.00	.20	.35	.40	.1866	.1618	.0000	.3022	.3494	.1253	.5936	.0996	.0996	.0996	.3525	.3489	.0673	.8991
IE2	.05	.15	.25	.25	.45	.1866	.1618	.0000	.3022	.3494	.1372	.4497	.0996	.0996	.0996	.3525	.3489	.0983	.7341
IE3	.00	.10	.35	.20	.40	.1866	.1618	.0000	.3022	.3494	.1866	.0700	.0996	.0996	.0996	.3525	.3489	.1404	.4871
IE4	.05	.20	.20	.30	.35	.1866	.1618	.0000	.3022	.3494	.1096	.5517	.0996	.0996	.0996	.3525	.3489	.0713	.8312
EI1	.00	.20	.30	.25	.30	.1866	.1618	.0000	.3022	.3494	.1621	.0403	.0996	.0996	.0996	.3525	.3489	.1209	.4753
EI2	.05	.05	.35	.30	.35	.1866	.1618	.0000	.3022	.3494	.1753	.1215	.0996	.0996	.0996	.3525	.3489	.1186	.6113
EI3	.00	.10	.30	.25	.40	.1866	.1618	.0000	.3022	.3494	.1637	.2468	.0996	.0996	.0996	.3525	.3489	.1124	.6543
EI4	.05	.30	.20	.30	.25	.1866	.1618	.0000	.3022	.3494	.1324	.2809	.0996	.0996	.0996	.3525	.3489	.1142	.4858
EE1	.15	.05	.20	.30	.35	.2670	.1214	.0000	.1766	.4349	.1275	.5308	.0996	.0996	.0996	.3525	.3489	.0597	.8780
EE2	.15	.10	.20	.30	.40	.2670	.1214	.0000	.1766	.4349	.1188	.6257	.0996	.0996	.0996	.3525	.3489	.0600	.9070
EE3	.10	.00	.20	.30	.45	.2670	.1214	.0000	.1766	.4349	.1401	.5741	.0996	.0996	.0996	.3525	.3489	.0812	.8596
EE4	.15	.00	.20	.35	.40	.2670	.1214	.0000	.1766	.4349	.1412	.5334	.0996	.0996	.0996	.3525	.3489	.0709	.8807
Mean											.1233	.7675						.0927	.8591

## Footnotes

Appendix A Supplementary data associated with this article can be found in the online version at doi:10.1016/j.dib.2016.09.053.
