# Supplementary material for: Model fitting data from syllogistic reasoning experiments
Source: Data Brief. 2016 Oct 14;9:850–75. doi: 10.1016/j.dib.2016.09.053 (PMC5109289; doi:10.1016/j.dib.2016.09.053)
Supplement: Supplementary file 1 — Supplementary material [file mmc1.docx]

I wish to confirm that there are no known conflicts of interest associated with this publication and there has been no significant financial support for this work that could have influenced its outcome.

I confirm that I have given due consideration to the protection of intellectual property associated with this work and that there are no impediments to publication, including the timing of publication, with respect to intellectual property. In so doing I confirm that I have followed the regulations of our institutions concerning intellectual property.

I further confirm that any aspect of the work covered in this manuscript that has involved either experimental animals or human patients has been conducted with the ethical approval of all relevant bodies and that such approvals are acknowledged within the manuscript.

I understand that the Corresponding Author is the sole contact for the Editorial process (including Editorial Manager and direct communications with the office). He is responsible for submissions of revisions and final approval of proofs. I confirm that I have provided a current, correct email address which is accessible by the Corresponding Author and which has been configured to accept email from hat@lt.ritsumei.ac.jp.

Signed by all authors as follows:


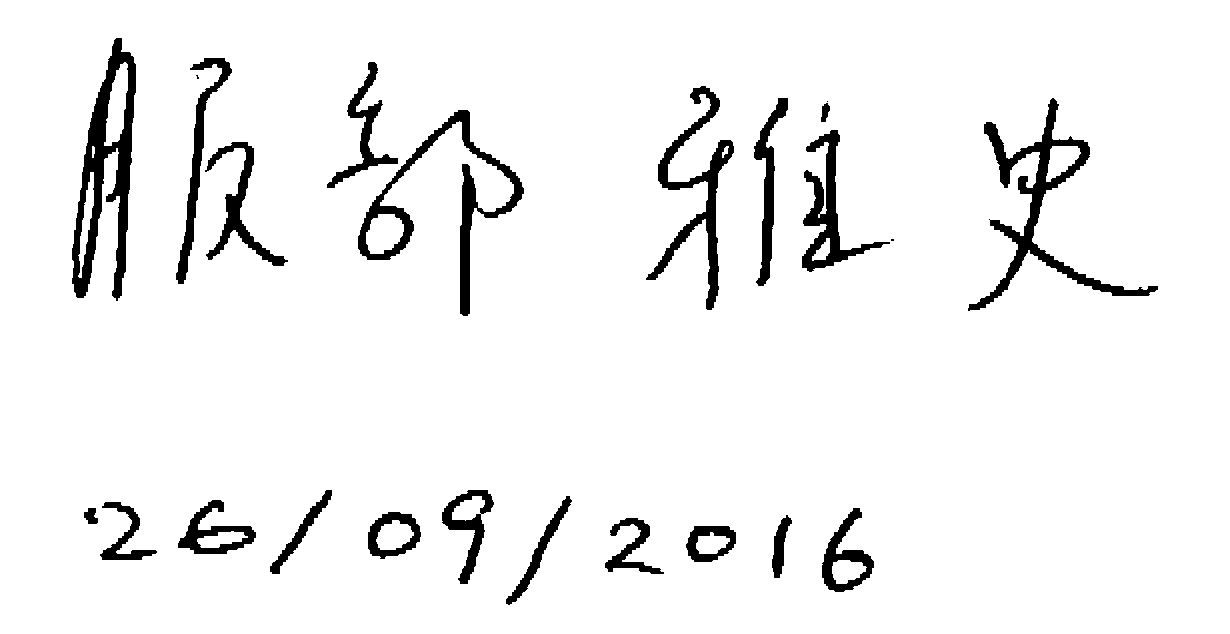


Masasi Hattori
