# Supplementary material for: Model fitting data from syllogistic reasoning experiments
Source: Data Brief. 2016 Oct 14;9:850–75. doi: 10.1016/j.dib.2016.09.053 (PMC5109289; doi:10.1016/j.dib.2016.09.053)
Supplement: Supplementary file 3 — Supplementary material [file mmc3.pdf]

Table 10. Data from Roberts, Newstead, and Griggs's (2001) [8] Experiment and models' predictions.

	Data (N=56)					PRT							PHM							pMM						
Type	]	I	E	O	N	A	I	E	O	N	RMSD	r	A	I	E	O	N	RMSD	r	A	I	E	O	N	RMSD	r
AA1	.8036	.0179	.0357	.0000	.1429	.8411	.0000	.0000	.0000	.1589	.0255	.9989	.7330	.0828	.0264	.0264	.1315	.0450	.9958	.8305	.0424	.0424	.0424	.0424	.0515	.9867
AA2	.6786	.0536	.0179	.0000	.2500	.7854	.0548	.0000	.0001	.1597	.0631	.9861	.7330	.0828	.0264	.0264	.1315	.0610	.9743	.4936	.0590	.0590	.0590	.3294	.0956	.9619
AA3	.7500	.0536	.0357	.0000	.1607	.7774	.0629	.0000	.0001	.1596	.0206	.9987	.7330	.0828	.0264	.0264	.1315	.0236	.9971	.8305	.0424	.0424	.0424	.0424	.0670	.9815
AA4	.8571	.0179	.0179	.0000	.1071	.7230	.1164	.0000	.0002	.1603	.0786	.9867	.7330	.0828	.0264	.0264	.1315	.0648	.9975	.8305	.0424	.0424	.0424	.0424	.0398	.9936
AI1	.0179	.8929	.0357	.0000	.0536	.0024	.6957	.0251	.1032	.1735	.1134	.9750	.0264	.7330	.0264	.0828	.1315	.0879	.9908	.0424	.8305	.0424	.0424	.0424	.0359	.9987
AI2	.0357	.7143	.0357	.0357	.1786	.0022	.6822	.0300	.1122	.1734	.0402	.9893	.0264	.7330	.0264	.0828	.1315	.0315	.9933	.0590	.4936	.0590	.0590	.3294	.1209	.9181
AI3	.0179	.7857	.0179	.0536	.1250	.0024	.6957	.0251	.1032	.1735	.0514	.9933	.0264	.7330	.0264	.0828	.1315	.0276	.9995	.0424	.8305	.0424	.0424	.0424	.0451	.9912
AI4	.0000	.8571	.0179	.0357	.0893	.0022	.6822	.0300	.1122	.1734	.0935	.9871	.0264	.7330	.0264	.0828	.1315	.0635	.9979	.0590	.4936	.0590	.0590	.3294	.1978	.8595
IA1	.0357	.6964	.0179	.0536	.1964	.0026	.6822	.0300	.1106	.1746	.0322	.9923	.0264	.7330	.0264	.0828	.1315	.0362	.9918	.0590	.4936	.0590	.0590	.3294	.1105	.9300
IA2	.0000	.7679	.0179	.0714	.1429	.0026	.6822	.0300	.1106	.1746	.0448	.9970	.0264	.7330	.0264	.0828	.1315	.0212	.9994	.0590	.4936	.0590	.0590	.3294	.1519	.8902
IA3	.0536	.7857	.0357	.0000	.1250	.0020	.6957	.0251	.1049	.1722	.0694	.9789	.0264	.7330	.0264	.0828	.1315	.0458	.9912	.0424	.8305	.0424	.0424	.0424	.0465	.9905
IA4	.0179	.8929	.0000	.0357	.0536	.0020	.6957	.0251	.1049	.1722	.1083	.9814	.0264	.7330	.0264	.0828	.1315	.0832	.9951	.0424	.8305	.0424	.0424	.0424	.0359	.9987
AE1	.0000	.0179	.7679	.0179	.1964	.0000	.0000	.8103	.0304	.1593	.0270	.9977	.0264	.0264	.7330	.0828	.1315	.0456	.9903	.0175	.0175	.5648	.1633	.2368	.1134	.9621
AE2	.0179	.0357	.8393	.0000	.1071	.0000	.0000	.8411	.0000	.1589	.0292	.9960	.0264	.0264	.7330	.0828	.1315	.0615	.9940	.0424	.0424	.8305	.0424	.0424	.0366	.9936
AE3	.0000	.0000	.7321	.0714	.1964	.0000	.0000	.8103	.0304	.1593	.0428	.9965	.0264	.0264	.7330	.0828	.1315	.0339	.9925	.0175	.0175	.5648	.1633	.2368	.0880	.9801
AE4	.0000	.0179	.8571	.0179	.1071	.0000	.0000	.8411	.0000	.1589	.0267	.9968	.0264	.0264	.7330	.0828	.1315	.0648	.9975	.0424	.0424	.8305	.0424	.0424	.0398	.9936
EA1	.0000	.0536	.7679	.0357	.1429	.0000	.0000	.8411	.0000	.1589	.0442	.9975	.0264	.0264	.7330	.0828	.1315	.0316	.9958	.0424	.0424	.8305	.0424	.0424	.0565	.9865
EA2	.0000	.0357	.8214	.0357	.1071	.0000	.0000	.8411	.0000	.1589	.0335	.9954	.0264	.0264	.7330	.0828	.1315	.0478	.9976	.0424	.0424	.8305	.0424	.0424	.0351	.9938
EA3	.0179	.0357	.7857	.0536	.1071	.0000	.0000	.8103	.0305	.1592	.0330	.9955	.0264	.0264	.7330	.0828	.1315	.0296	.9984	.0175	.0175	.5648	.1633	.2368	.1249	.9416
EA4	.0179	.0179	.8214	.0357	.1071	.0000	.0000	.8103	.0305	.1592	.0265	.9964	.0264	.0264	.7330	.0828	.1315	.0464	.9982	.0175	.0175	.5648	.1633	.2368	.1407	.9410
AO1	.0357	.2321	.0179	.5714	.1429	.0169	.0847	.0031	.7218	.1735	.0958	.9560	.0264	.0828	.0264	.7330	.1315	.0987	.9530	.0590	.0590	.0590	.4936	.3294	.1209	.8047
AO2	.0179	.1786	.0536	.6071	.1429	.0141	.0768	.0034	.7324	.1734	.0768	.9814	.0264	.0828	.0264	.7330	.1315	.0720	.9836	.0590	.0590	.0590	.4936	.3294	.1129	.8461
AO3	.0179	.1786	.0357	.5536	.2143	.0169	.0847	.0031	.7218	.1735	.0893	.9773	.0264	.0828	.0264	.7330	.1315	.0984	.9633	.0590	.0590	.0590	.4936	.3294	.0817	.9062
AO4	.0000	.0714	.0357	.6964	.1964	.0141	.0768	.0034	.7324	.1734	.0249	.9974	.0264	.0828	.0264	.7330	.1315	.0360	.9918	.0590	.0590	.0590	.4936	.3294	.1122	.9273
OA1	.0000	.1250	.0357	.5893	.2500	.0169	.0830	.0037	.7218	.1746	.0726	.9803	.0264	.0828	.0264	.7330	.1315	.0863	.9628	.0590	.0590	.0590	.4936	.3294	.0690	.9518
OA2	.0000	.2143	.0357	.5357	.2143	.0169	.0830	.0037	.7218	.1746	.1046	.9522	.0264	.0828	.0264	.7330	.1315	.1130	.9370	.0590	.0590	.0590	.4936	.3294	.0929	.8752
OA3	.0000	.1250	.0714	.6607	.1429	.0137	.0774	.0028	.7339	.1722	.0517	.9904	.0264	.0828	.0264	.7330	.1315	.0444	.9934	.0590	.0590	.0590	.4936	.3294	.1189	.8697
OA4	.0179	.1786	.0000	.6607	.1429	.0137	.0774	.0028	.7339	.1722	.0574	.9833	.0264	.0828	.0264	.7330	.1315	.0553	.9828	.0590	.0590	.0590	.4936	.3294	.1282	.8522
II1	.0000	.6786	.0000	.0357	.2857	.0017	.5265	.0211	.0826	.3680	.0807	.9657	.0264	.5193	.0264	.0828	.3452	.0807	.9763	.0590	.4936	.0590	.0590	.3294	.0934	.9784
II2	.0357	.6429	.0179	.0536	.2500	.0017	.5265	.0211	.0826	.3680	.0768	.9480	.0264	.5193	.0264	.0828	.3452	.0712	.9616	.0590	.4936	.0590	.0590	.3294	.0785	.9650
II3	.0179	.2857	.1250	.0893	.4821	.0017	.5265	.0211	.0826	.3680	.1281	.7916	.0264	.5193	.0264	.0828	.3452	.1289	.7638	.0590	.4936	.0590	.0590	.3294	.1213	.7579
II4	.0000	.6786	.0179	.0000	.3036	.0017	.5265	.0211	.0826	.3680	.0826	.9669	.0264	.5193	.0264	.0828	.3452	.0834	.9769	.0590	.4936	.0590	.0590	.3294	.0933	.9852
IE1	.0000	.0179	.5714	.1429	.2679	.0096	.0503	.1166	.4554	.3680	.2513	.1670	.0264	.0264	.5193	.0828	.3452	.0512	.9699	.0175	.0175	.5648	.1633	.2368	.0186	.9967
IE2	.0000	.0714	.4643	.2857	.1786	.0096	.0503	.1166	.4554	.3680	.1929	.3667	.0264	.0264	.5193	.0828	.3452	.1222	.7888	.0175	.0175	.5648	.1633	.2368	.0796	.9250
IE3	.0179	.0179	.3571	.2857	.3214	.0096	.0503	.1166	.4554	.3680	.1341	.6793	.0264	.0264	.5193	.0828	.3452	.1168	.8104	.0175	.0175	.5648	.1633	.2368	.1142	.8269
IE4	.0000	.0893	.3214	.3929	.1964	.0096	.0503	.1166	.4554	.3680	.1240	.7237	.0264	.0264	.5193	.0828	.3452	.1800	.4856	.0175	.0175	.5648	.1633	.2368	.1543	.6458
EI1	.0000	.0714	.3036	.4107	.2143	.0096	.0503	.1166	.4554	.3680	.1106	.7864	.0264	.0264	.5193	.0828	.3452	.1865	.4545	.0175	.0175	.5648	.1633	.2368	.1632	.6017
EI2	.0000	.0179	.5000	.2857	.1964	.0096	.0503	.1166	.4554	.3680	.2032	.3741	.0264	.0264	.5193	.0828	.3452	.1136	.8268	.0175	.0175	.5648	.1633	.2368	.0650	.9468
EI3	.0000	.0893	.4643	.1250	.3214	.0096	.0503	.1166	.4554	.3680	.2163	.2249	.0264	.0264	.5193	.0828	.3452	.0448	.9833	.0175	.0175	.5648	.1633	.2368	.0695	.9442
EI4	.0000	.0179	.6429	.0357	.3036	.0096	.0503	.1166	.4554	.3680	.3028	.0174	.0264	.0264	.5193	.0828	.3452	.0632	.9840	.0175	.0175	.5648	.1633	.2368	.0737	.9674
IO1	.0000	.2679	.0536	.3036	.3750	.0117	.0615	.0025	.5563	.3680	.1478	.7554	.0264	.0828	.0264	.5193	.3452	.1289	.7608	.0590	.0590	.0590	.4936	.3294	.1306	.6987
IO2	.0357	.2679	.0357	.2679	.3929	.0117	.0615	.0025	.5563	.3680	.1601	.6996	.0264	.0828	.0264	.5193	.3452	.1414	.7022	.0590	.0590	.0590	.4936	.3294	.1412	.6390
IO3	.0000	.0893	.0714	.3393	.5000	.0117	.0615	.0025	.5563	.3680	.1185	.8473	.0264	.0828	.0264	.5193	.3452	.1087	.8435	.0590	.0590	.0590	.4936	.3294	.1072	.8322
IO4	.0000	.1250	.0179	.5179	.3393	.0117	.0615	.0025	.5563	.3680	.0366	.9911	.0264	.0828	.0264	.5193	.3452	.0228	.9935	.0590	.0590	.0590	.4936	.3294	.0452	.9768
OI1	.0179	.1250	.0000	.6071	.2500	.0117	.0615	.0025	.5563	.3680	.0642	.9585	.0264	.0828	.0264	.5193	.3452	.0622	.9626	.0590	.0590	.0590	.4936	.3294	.0758	.9503
OI2	.0000	.2500	.0179	.3214	.4107	.0117	.0615	.0025	.5563	.3680	.1363	.7937	.0264	.0828	.0264	.5193	.3452	.1201	.7967	.0590	.0590	.0590	.4936	.3294	.1248	.7412
OI3	.0000	.2500	.0714	.3214	.3571	.0117	.0615	.0025	.5563	.3680	.1384	.8036	.0264	.0828	.0264	.5193	.3452	.1183	.8096	.0590	.0590	.0590	.4936	.3294	.1188	.7529
OI4	.0000	.2857	.0357	.2857	.3929	.0117	.0615	.0025	.5563	.3680	.1583	.7046	.0264	.0828	.0264	.5193	.3452	.1406	.7094	.0590	.0590	.0590	.4936	.3294	.1433	.6430
EE1	.0357	.0000	.4821	.0000	.4821	.1658	.0883	.0108	.1404	.5947	.2363	.4151	.0264	.0264	.3067	.0828	.5577	.0939	.9136	.0590	.0590	.4936	.0590	.3294	.0787	.9561
EE2	.0179	.0357	.2321	.0000	.7143	.1658	.0883	.0108	.1404	.5947	.1467	.8447	.0264	.0264	.3067	.0828	.5577	.0861	.9701	.0590	.0590	.4936	.0590	.3294	.2108	.6275
EE3	.0179	.0357	.2857	.0000	.6607	.1658	.0883	.0108	.1404	.5947	.1577	.7823	.0264	.0264	.3067	.0828	.5577	.0601	.9860	.0590	.0590	.4936	.0590	.3294	.1782	.7097
EE4	.0000	.0179	.3036	.0000	.6786	.1658	.0883	.0108	.1404	.5947	.1702	.7677	.0264	.0264	.3067	.0828	.5577	.0667	.9913	.0590	.0590	.4936	.0590	.3294	.1826	.7281
EO1	.0000	.0536	.2679	.2857	.3929	.0074	.0390	.0016	.3526	.5994	.1538	.7812	.0264	.0828	.0264	.3067	.5577	.1323	.7698	.0590	.0590	.4936	.0590	.3294	.1482	.6088
EO2	.0000	.0893	.2857	.2143	.4107	.0074	.0390	.0016	.3526	.5994	.1662	.7290	.0264	.0828	.0264	.3067	.5577	.1401	.7358	.0590	.0590	.4936	.0590	.3294	.1252	.7239
EO3	.0000	.0357	.2321	.1786	.5536	.0074	.0390	.0016	.3526	.5994	.1309	.8366	.0264	.0828	.0264	.3067	.5577	.1111	.8494	.0590	.0590	.4936	.0590	.3294	.1655	.6177
EO4	.0357	.1071	.2857	.5536	.0179	.0074	.0390	.0016	.3526	.5994	.3049	.0451	.0264	.0828	.0264	.3067	.5577	.2899	-.0140	.0590	.0590	.4936	.0590	.3294	.2785	-.0663
OE1	.0000	.0179	.2679	.1071	.6071	.0074	.0390	.0016	.3526	.5994	.1623	.7563	.0264	.0828	.0264	.3067	.5577	.1453	.7761	.0590	.0590	.4936	.0590	.3294	.1647	.6893
OE2	.0000	.0000	.2857	.2143	.5000	.0074	.0390	.0016	.3526	.5994	.1492	.7808	.0264	.0828	.0264	.3067	.5577	.1317	.7816	.0590	.0590	.4936	.0590	.3294	.1438	.6966
OE3	.0000	.0179	.2679	.2857	.4286	.0074	.0390	.0016	.3526	.5994	.1450	.8020	.0264	.0828	.0264	.3067	.5577	.1268	.7897	.0590	.0590	.4936	.0590	.3294	.1532	.6108
OE4	.0000	.1071	.1786	.3393	.3750	.0074	.0390	.0016	.3526	.5994	.1316	.8855	.0264	.0828	.0264	.3067	.5577	.1085	.8719	.0590	.0590	.4936	.0590	.3294	.1927	.2994
OO1	.0179	.0893	.0714	.5179	.3036	.0109	.0574	.0023	.5196	.4097	.0585	.9717	.0264	.0828	.0264	.4853	.3791	.0422	.9760	.0590	.0590	.0590	.4936	.3294	.0283	.9886
OO2	.0000	.1429	.0179	.4107	.4286	.0109	.0574	.0023	.5196	.4097	.0630	.9652	.0264	.0828	.0264	.4853	.3791	.0498	.9664	.0590	.0590	.0590	.4936	.3294	.0760	.9143
OO3	.0179	.0357	.0893	.3214	.5357	.0109	.0574	.0023	.5196	.4097	.1124	.8605	.0264	.0828	.0264	.4853	.3791	.1074	.8517	.0590	.0590	.0590	.4936	.3294	.1228	.7963
OO4	.0000	.1071	.0179	.5536	.3214	.0109	.0574	.0023	.5196	.4097	.0486	.9754	.0264	.0828	.0264	.4853	.3791	.0432	.9805	.0590	.0590	.0590	.4936	.3294	.0472	.9826
Mean											.1059	.9520						.0840	.9707						.1059	.9296

Table 11. Data from Hattori's (in press) [1] Experiment 1 and models' predictions

	Data (N=88)					PRT							PHM							pMM						
Type	A	I	E	O	N	A	I	E	O	N	RMSD	r	A	I	E	O	N	RMSD	r	A	I	E	O	N	RMSD	r
AA1	.9659	.0227	.0000	.0114	.0000	.8324	.0000	.0000	.0000	.1676	.0965	.9770	.6725	.1141	.0188	.0188	.1757	.1586	.9694	.8322	.0420	.0420	.0420	.0420	.0674	.9998
AA2	.1818	.2500	.0000	.0568	.5114	.4706	.3384	.0015	.0095	.1800	.2016	.3819	.6725	.1141	.0188	.0188	.1757	.2734	.1913	.5602	.0554	.0554	.0554	.2737	.2194	.3301
AA3	.3636	.3068	.0000	.0341	.2955	.4694	.3607	.0000	.0010	.1689	.0790	.9159	.6725	.1141	.0188	.0188	.1757	.1717	.7156	.8322	.0420	.0420	.0420	.0420	.2667	.5399
AA4	.2955	.4091	.0000	.0909	.2045	.1828	.6318	.0005	.0135	.1714	.1178	.8977	.6725	.1141	.0188	.0188	.1757	.2171	.4709	.8322	.0420	.0420	.0420	.0420	.3012	.3297
AI1	.0341	.8750	.0114	.0455	.0341	.0028	.7622	.0059	.0509	.1782	.0831	.9771	.0188	.6725	.0188	.1141	.1757	.1149	.9734	.0420	.8322	.0420	.0420	.0420	.0241	.9995
AI2	.0341	.5568	.0000	.1364	.2727	.0010	.6586	.0363	.1260	.1782	.0661	.9678	.0188	.6725	.0188	.1141	.1757	.0691	.9691	.0554	.5602	.0554	.0554	.2737	.0449	.9750
IA1	.0227	.7159	.0000	.0795	.1818	.0059	.6586	.0363	.1090	.1902	.0341	.9968	.0188	.6725	.0188	.1141	.1757	.0264	.9983	.0554	.5602	.0554	.0554	.2737	.0865	.9711
IA3	.0114	.7955	.0000	.1250	.0682	.0004	.7622	.0059	.0589	.1725	.0575	.9819	.0188	.6725	.0188	.1141	.1757	.0738	.9854	.0420	.8322	.0420	.0420	.0420	.0482	.9890
AE1	.0000	.1023	.4432	.1023	.3523	.0001	.0011	.5869	.2417	.1702	.1292	.8002	.0188	.0188	.6725	.1141	.1757	.1351	.8469	.0568	.0568	.3864	.1761	.3239	.0544	.9587
AE2	.0000	.0227	.8523	.0341	.0909	.0000	.0000	.8324	.0000	.1676	.0399	.9926	.0188	.0188	.6725	.1141	.1757	.0962	.9860	.0420	.0420	.8322	.0420	.0420	.0316	.9958
EA1	.0114	.0227	.8295	.0455	.0909	.0000	.0000	.8324	.0000	.1676	.0415	.9918	.0188	.0188	.6725	.1141	.1757	.0856	.9863	.0420	.0420	.8322	.0420	.0420	.0273	.9963
EA3	.0114	.1136	.3295	.2500	.2955	.0000	.0003	.5869	.2445	.1683	.1382	.8091	.0188	.0188	.6725	.1141	.1757	.1786	.7171	.0568	.0568	.3864	.1761	.3239	.0544	.9164
Mean											.0904	.9595						.1334	.9415						.1022	.9762

Table 12. Data from Hattori's (in press) [1] Experiment 2 and models' predictions

	Data (N=50)					PRT							PHM							pMM						
Type	A	I	E	O	N	A	I	E	O	N	RMSD	r	A	I	E	O	N	RMSD	r	A	I	E	O	N	RMSD	r
AA1	1.00	.00	.00	.00	.00	.7878	.0000	.0000	.0000	.2122	.1342	.9630	.5600	.1829	.0213	.0213	.2146	.2341	.9138	.8513	.0372	.0372	.0372	.0372	.0743	#####
AA2	.16	.14	.00	.10	.60	.4279	.3286	.0027	.0130	.2277	.2252	.2987	.5600	.1829	.0213	.0213	.2146	.2518	.2257	.4741	.0896	.0896	.0896	.2572	.2130	.3284
AA3	.42	.26	.00	.04	.28	.4117	.3736	.0000	.0012	.2136	.0615	.9379	.5600	.1829	.0213	.0213	.2146	.0783	.9264	.8513	.0372	.0372	.0372	.0372	.2433	.6985
AA4	.30	.44	.00	.08	.18	.1374	.6270	.0008	.0186	.2162	.1153	.8844	.5600	.1829	.0213	.0213	.2146	.1666	.5762	.8513	.0372	.0372	.0372	.0372	.3130	.3196
AI1	.04	.88	.00	.04	.04	.0031	.7166	.0064	.0505	.2233	.1112	.9593	.0213	.5600	.0213	.1829	.2146	.1756	.9234	.0372	.8513	.0372	.0372	.0372	.0211	.9990
AI2	.02	.54	.02	.14	.28	.0009	.5972	.0461	.1328	.2230	.0390	.9853	.0213	.5600	.0213	.1829	.2146	.0361	.9831	.0896	.4741	.0896	.0896	.2572	.0585	.9742
AI3	.14	.70	.00	.08	.08	.0031	.7166	.0064	.0505	.2233	.0900	.9431	.0213	.5600	.0213	.1829	.2146	.1121	.9067	.0372	.8513	.0372	.0372	.0372	.0878	.9845
AI4	.04	.60	.00	.16	.20	.0009	.5972	.0461	.1328	.2230	.0314	.9891	.0213	.5600	.0213	.1829	.2146	.0251	.9956	.0896	.4741	.0896	.0896	.2572	.0832	.9516
IA1	.06	.60	.00	.12	.22	.0070	.5972	.0461	.1127	.2370	.0325	.9884	.0213	.5600	.0213	.1829	.2146	.0388	.9850	.0896	.4741	.0896	.0896	.2572	.0736	.9741
IA3	.04	.80	.00	.08	.08	.0004	.7166	.0064	.0598	.2168	.0744	.9721	.0213	.5600	.0213	.1829	.2146	.1320	.9452	.0372	.8513	.0372	.0372	.0372	.0392	.9951
IA4	.06	.66	.00	.10	.18	.0004	.7166	.0064	.0598	.2168	.0442	.9930	.0213	.5600	.0213	.1829	.2146	.0633	.9746	.0372	.8513	.0372	.0372	.0372	.1121	.9692
AE1	.00	.04	.48	.14	.34	.0001	.0016	.5296	.2531	.2156	.0803	.9121	.0213	.0213	.5600	.1829	.2146	.0704	.9341	.0411	.0411	.4027	.1816	.3335	.0434	.9874
AE2	.02	.00	.94	.00	.04	.0000	.0000	.7878	.0000	.2122	.1032	.9717	.0213	.0213	.5600	.1829	.2146	.2044	.9197	.0372	.0372	.8513	.0372	.0372	.0468	.9992
AE3	.04	.06	.34	.18	.38	.0001	.0016	.5296	.2531	.2156	.1211	.7883	.0213	.0213	.5600	.1829	.2146	.1246	.7776	.0411	.0411	.4027	.1816	.3335	.0359	.9706
AE4	.00	.04	.88	.00	.08	.0000	.0000	.7878	.0000	.2122	.0743	.9798	.0213	.0213	.5600	.1829	.2146	.1759	.9247	.0372	.0372	.8513	.0372	.0372	.0329	.9962
EA1	.00	.04	.94	.02	.00	.0000	.0000	.7878	.0000	.2122	.1185	.9566	.0213	.0213	.5600	.1829	.2146	.2087	.9073	.0372	.0372	.8513	.0372	.0372	.0468	.9992
EA2	.02	.02	.86	.00	.10	.0000	.0000	.7878	.0000	.2122	.0610	.9851	.0213	.0213	.5600	.1829	.2146	.1653	.9300	.0372	.0372	.8513	.0372	.0372	.0346	.9946
EA3	.02	.04	.34	.34	.26	.0000	.0003	.5296	.2571	.2129	.0970	.8833	.0213	.0213	.5600	.1829	.2146	.1229	.7841	.0411	.0411	.4027	.1816	.3335	.0835	.8351
EA4	.02	.08	.42	.16	.32	.0000	.0003	.5296	.2571	.2129	.0890	.9018	.0213	.0213	.5600	.1829	.2146	.0833	.9209	.0411	.0411	.4027	.1816	.3335	.0241	.9869
AO1	.00	.24	.04	.56	.16	.0380	.1229	.0011	.6146	.2233	.0688	.9516	.0213	.1829	.0213	.5600	.2146	.0376	.9821	.0896	.0896	.0896	.4741	.2572	.0999	.8721
AO2	.00	.28	.08	.48	.16	.0052	.0444	.0034	.7240	.2230	.1581	.8516	.0213	.1829	.0213	.5600	.2146	.0674	.9441	.0896	.0896	.0896	.4741	.2572	.1038	.7935
AO3	.02	.48	.02	.24	.24	.0380	.1229	.0011	.6146	.2233	.2319	.3220	.0213	.1829	.0213	.5600	.2146	.1956	.4422	.0896	.0896	.0896	.4741	.2572	.2084	.1702
AO4	.00	.34	.02	.38	.26	.0052	.0444	.0034	.7240	.2230	.2037	.6759	.0213	.1829	.0213	.5600	.2146	.1092	.8326	.0896	.0896	.0896	.4741	.2572	.1299	.6534
OA1	.00	.20	.00	.58	.22	.0380	.1024	.0079	.6146	.2370	.0500	.9744	.0213	.1829	.0213	.5600	.2146	.0180	.9988	.0896	.0896	.0896	.4741	.2572	.0904	.9297
OA2	.00	.36	.04	.22	.38	.0380	.1024	.0079	.6146	.2370	.2214	.3577	.0213	.1829	.0213	.5600	.2146	.1871	.4606	.0896	.0896	.0896	.4741	.2572	.1807	.3171
OA3	.00	.40	.00	.50	.10	.0045	.0502	.0005	.7281	.2168	.1939	.7121	.0213	.1829	.0213	.5600	.2146	.1138	.8451	.0896	.0896	.0896	.4741	.2572	.1660	.6199
OA4	.00	.42	.10	.22	.26	.0045	.0502	.0005	.7281	.2168	.2852	.1910	.0213	.1829	.0213	.5600	.2146	.1900	.4110	.0896	.0896	.0896	.4741	.2572	.1907	.1635
II3	.00	.42	.02	.12	.44	.0013	.4589	.0126	.0607	.4666	.0340	.9925	.0213	.3773	.0213	.1829	.3973	.0401	.9871	.0896	.4741	.0896	.0896	.2572	.1001	.9925
Mean											.1125	.9309						.1224	.9228						.1049	.9857

Table 13. Data from Chater and Oaksford's (1999) [3] Experiment 1 and models' predictions.

	Data (N=20)					PRT							PHM						
Type	A	M	F	O	N	A	M	F	O	N	RMSD	r	A	M	F	O	N	RMSD	r
AA1	.85	.00	.00	.10	.05	.8787	.0000	.0000	.0000	.1213	.0564	.9869	.6252	.0306	.0306	.0306	.2831	.1494	.9109
AA2	.60	.05	.10	.20	.20	.8150	.0535	.0102	.0000	.1213	.1417	.9534	.6252	.0306	.0306	.0306	.2831	.0910	.9363
AA3	.65	.00	.05	.15	.20	.7008	.1548	.0230	.0000	.1213	.1058	.9123	.6252	.0306	.0306	.0306	.2831	.0680	.9583
AA4	.55	.15	.10	.15	.25	.6391	.2046	.0349	.0000	.1213	.1041	.9389	.6252	.0306	.0306	.0306	.2831	.0896	.9829
AM1	.05	.85	.00	.40	.00	.0000	.7099	.1687	.0000	.1213	.2123	.7881	.0306	.6252	.0306	.2791	.0345	.1163	.9974
AM2	.05	.60	.05	.35	.20	.0000	.6925	.1862	.0000	.1213	.1779	.7486	.0306	.6252	.0306	.2791	.0345	.0822	.9629
AM3	.10	.60	.10	.40	.10	.0000	.7099	.1687	.0000	.1213	.1935	.7186	.0306	.6252	.0306	.2791	.0345	.0764	.9845
AM4	.05	.70	.10	.35	.05	.0000	.6925	.1862	.0000	.1213	.1659	.8059	.0306	.6252	.0306	.2791	.0345	.0567	.9966
MA1	.05	.65	.10	.40	.05	.0000	.6925	.1862	.0000	.1213	.1881	.7342	.0306	.6252	.0306	.2791	.0345	.0643	.9856
MA2	.10	.50	.10	.35	.15	.0000	.6925	.1862	.0000	.1213	.1886	.7000	.0306	.6252	.0306	.2791	.0345	.0935	.9773
MA3	.05	.65	.15	.50	.05	.0000	.7099	.1687	.0000	.1213	.2287	.6508	.0306	.6252	.0306	.2791	.0345	.1134	.9462
MA4	.20	.55	.15	.30	.05	.0000	.7099	.1687	.0000	.1213	.1795	.7659	.0306	.6252	.0306	.2791	.0345	.0993	.9571
AF1	.05	.00	.85	.40	.00	.0000	.0000	.8787	.0000	.1213	.1887	.8594	.0306	.0306	.6252	.2791	.0345	.1163	.9974
AF2	.05	.00	.85	.25	.10	.0000	.0000	.8787	.0000	.1213	.1151	.9541	.0306	.0306	.6252	.2791	.0345	.1068	.9809
AF3	.05	.00	.80	.35	.05	.0000	.0000	.8787	.0000	.1213	.1651	.8883	.0306	.0306	.6252	.2791	.0345	.0862	.9983
AF4	.05	.00	.75	.25	.15	.0000	.0000	.8787	.0000	.1213	.1284	.9486	.0306	.0306	.6252	.2791	.0345	.0788	.9735
FA1	.10	.00	.80	.30	.05	.0000	.0000	.8787	.0000	.1213	.1492	.9138	.0306	.0306	.6252	.2791	.0345	.0860	.9906
FA2	.05	.05	.70	.30	.15	.0000	.0000	.8787	.0000	.1213	.1598	.9212	.0306	.0306	.6252	.2791	.0345	.0634	.9874
FA3	.10	.05	.60	.45	.00	.0000	.0000	.8787	.0000	.1213	.2479	.6998	.0306	.0306	.6252	.2791	.0345	.0851	.9499
FA4	.05	.05	.65	.45	.05	.0000	.0000	.8787	.0000	.1213	.2302	.7551	.0306	.0306	.6252	.2791	.0345	.0785	.9723
AO1	.05	.25	.10	.90	.05	.0546	.0668	.0977	.6596	.1213	.1389	.9608	.0306	.1283	.1283	.6252	.0876	.1363	.9852
AO2	.05	.15	.10	.75	.10	.0451	.0545	.0994	.6797	.1213	.0539	.9874	.0306	.1283	.1283	.6252	.0876	.0590	.9962
AO3	.05	.10	.10	.80	.15	.0546	.0668	.0977	.6596	.1213	.0658	.9988	.0306	.1283	.1283	.6252	.0876	.0854	.9892
AO4	.05	.10	.10	.80	.15	.0451	.0545	.0994	.6797	.1213	.0590	.9981	.0306	.1283	.1283	.6252	.0876	.0854	.9892
OA1	.10	.20	.10	.85	.10	.0546	.0582	.1062	.6596	.1213	.1085	.9769	.0306	.1283	.1283	.6252	.0876	.1109	.9884
OA2	.05	.15	.20	.65	.10	.0546	.0582	.1062	.6596	.1213	.0596	.9758	.0306	.1283	.1283	.6252	.0876	.0368	.9952
OA3	.05	.10	.15	.85	.10	.0306	.0542	.0792	.7147	.1213	.0725	.9940	.0306	.1283	.1283	.6252	.0876	.1023	.9957
OA4	.10	.25	.30	.55	.10	.0306	.0542	.0792	.7147	.1213	.1546	.8654	.0306	.1283	.1283	.6252	.0876	.1048	.9317
MM1	.00	.65	.15	.35	.15	.0000	.6177	.2183	.0000	.1639	.1603	.7827	.0306	.6252	.0306	.2791	.0345	.0827	.9698
MM2	.00	.45	.20	.30	.25	.0000	.6177	.2183	.0000	.1639	.1587	.7407	.0306	.6252	.0306	.2791	.0345	.1464	.8198
MM3	.00	.50	.05	.50	.10	.0000	.6177	.2183	.0000	.1639	.2434	.4213	.0306	.6252	.0306	.2791	.0345	.1184	.8748
MM4	.00	.50	.15	.35	.15	.0000	.6177	.2183	.0000	.1639	.1681	.6880	.0306	.6252	.0306	.2791	.0345	.0992	.9319
MF1	.00	.00	.75	.25	.10	.0000	.0000	.8361	.0000	.1639	.1217	.9314	.0306	.0306	.6252	.2791	.0345	.0672	.9855
MF2	.00	.10	.60	.35	.15	.0000	.0000	.8361	.0000	.1639	.1941	.8284	.0306	.0306	.6252	.2791	.0345	.0704	.9704
MF3	.00	.10	.55	.50	.10	.0000	.0000	.8361	.0000	.1639	.2630	.6118	.0306	.0306	.6252	.2791	.0345	.1136	.9025
MF4	.00	.05	.70	.30	.10	.0000	.0000	.8361	.0000	.1639	.1517	.8948	.0306	.0306	.6252	.2791	.0345	.0482	.9925
FM1	.00	.10	.60	.50	.05	.0000	.0000	.8361	.0000	.1639	.2564	.6435	.0306	.0306	.6252	.2791	.0345	.1053	.9283
FM2	.00	.20	.50	.40	.10	.0000	.0000	.8361	.0000	.1639	.2518	.6465	.0306	.0306	.6252	.2791	.0345	.1133	.8963
FM3	.00	.10	.70	.30	.10	.0000	.0000	.8361	.0000	.1639	.1566	.8928	.0306	.0306	.6252	.2791	.0345	.0567	.9885
FM4	.00	.15	.65	.30	.10	.0000	.0000	.8361	.0000	.1639	.1739	.8700	.0306	.0306	.6252	.2791	.0345	.0641	.9767
MO1	.00	.10	.25	.80	.10	.0450	.0548	.0942	.6420	.1639	.1071	.9596	.0306	.1283	.1283	.6252	.0876	.0972	.9843
MO2	.00	.10	.20	.75	.15	.0450	.0548	.0942	.6420	.1639	.0736	.9803	.0306	.1283	.1283	.6252	.0876	.0726	.9878
MO3	.00	.30	.15	.65	.15	.0450	.0548	.0942	.6420	.1639	.1145	.8936	.0306	.1283	.1283	.6252	.0876	.0841	.9524
MO4	.00	.15	.20	.75	.15	.0450	.0548	.0942	.6420	.1639	.0826	.9727	.0306	.1283	.1283	.6252	.0876	.0721	.9925
OM1	.00	.20	.20	.60	.20	.0450	.0548	.0942	.6420	.1639	.0865	.9429	.0306	.1283	.1283	.6252	.0876	.0700	.9656
OM2	.00	.20	.25	.70	.15	.0450	.0548	.0942	.6420	.1639	.1009	.9388	.0306	.1283	.1283	.6252	.0876	.0779	.9795
OM3	.00	.25	.30	.75	.10	.0450	.0548	.0942	.6420	.1639	.1402	.8954	.0306	.1283	.1283	.6252	.0876	.1104	.9642
OM4	.00	.25	.15	.70	.15	.0450	.0548	.0942	.6420	.1639	.0968	.9379	.0306	.1283	.1283	.6252	.0876	.0717	.9796
FF1	.00	.05	.55	.35	.20	.0000	.0000	.7676	.0000	.2324	.1862	.7956	.0306	.0306	.6252	.2791	.0345	.0887	.9365
FF2	.00	.10	.55	.30	.20	.0000	.0000	.7676	.0000	.2324	.1723	.8497	.0306	.0306	.6252	.2791	.0345	.0886	.9436
FF3	.00	.10	.50	.35	.20	.0000	.0000	.7676	.0000	.2324	.2026	.7568	.0306	.0306	.6252	.2791	.0345	.1038	.9180
FF4	.00	.05	.60	.30	.20	.0000	.0000	.7676	.0000	.2324	.1560	.8715	.0306	.0306	.6252	.2791	.0345	.0772	.9531
FO1	.00	.05	.40	.60	.20	.0413	.0503	.0865	.5895	.2324	.1422	.8112	.0306	.1283	.0306	.6252	.1853	.1699	.7328
FO2	.00	.00	.55	.65	.10	.0413	.0503	.0865	.5895	.2324	.2192	.6667	.0306	.1283	.0306	.6252	.1853	.2429	.5844
FO3	.00	.05	.60	.60	.10	.0413	.0503	.0865	.5895	.2324	.2379	.5760	.0306	.1283	.0306	.6252	.1853	.2605	.4957
FO4	.00	.00	.55	.60	.10	.0413	.0503	.0865	.5895	.2324	.2176	.6299	.0306	.1283	.0306	.6252	.1853	.2430	.5419
OF1	.00	.05	.45	.40	.15	.0413	.0503	.0865	.5895	.2324	.1879	.5410	.0306	.1283	.0306	.6252	.1853	.2168	.4366
OF2	.00	.00	.45	.55	.15	.0413	.0503	.0865	.5895	.2324	.1701	.7095	.0306	.1283	.0306	.6252	.1853	.2001	.6141
OF3	.00	.00	.50	.55	.15	.0413	.0503	.0865	.5895	.2324	.1916	.6562	.0306	.1283	.0306	.6252	.1853	.2212	.5558
OF4	.00	.05	.65	.40	.15	.0413	.0503	.0865	.5895	.2324	.2690	.3166	.0306	.1283	.0306	.6252	.1853	.2976	.2021
OO1	.00	.10	.10	.80	.10	.0466	.0567	.0975	.6644	.1348	.0688	.9932	.0306	.1283	.0306	.6252	.1853	.0942	.9743
OO2	.00	.10	.20	.70	.15	.0466	.0567	.0975	.6644	.1348	.0567	.9807	.0306	.1283	.0306	.6252	.1853	.0864	.9442
OO3	.00	.15	.15	.60	.25	.0466	.0567	.0975	.6644	.1348	.0788	.9552	.0306	.1283	.0306	.6252	.1853	.0640	.9682
OO4	.00	.05	.20	.70	.20	.0466	.0567	.0975	.6644	.1348	.0604	.9780	.0306	.1283	.0306	.6252	.1853	.0912	.9392
Mean											.1525	.8994						.1064	.9653

Table 14. Data from Chater and Oaksford's (1999) [3] Experiment 2 and models' predictions.

	Data (N=20)					PRT							PHM						
Type	M	F	I	E	N	M	F	I	E	N	RMSD	r	M	F	I	E	N	RMSD	r
MM1	.45	.10	.20	.15	.20	.3982	.2798	.0000	.0000	.3220	.1499	.5091	.3525	.0996	.1706	.0996	.2778	.0616	.8773
MM2	.50	.15	.20	.05	.25	.3982	.2798	.0000	.0000	.3220	.1224	.7266	.3525	.0996	.1706	.0996	.2778	.0754	.9244
MM3	.35	.10	.45	.05	.15	.3982	.2798	.0000	.0000	.3220	.2321	-.0338	.3525	.0996	.1706	.0996	.2778	.1392	.4768
MM4	.50	.05	.30	.05	.20	.3982	.2798	.0000	.0000	.3220	.1847	.4055	.3525	.0996	.1706	.0996	.2778	.0995	.8597
MF1	.15	.45	.10	.10	.30	.0000	.6780	.0000	.0000	.3220	.1378	.9884	.0996	.3525	.1706	.0996	.2778	.0592	.9339
MF2	.20	.35	.20	.10	.25	.0000	.6780	.0000	.0000	.3220	.2014	.8931	.0996	.3525	.1706	.0996	.2778	.0484	.9030
MF3	.15	.30	.20	.10	.30	.0000	.6780	.0000	.0000	.3220	.2078	.8346	.0996	.3525	.1706	.0996	.2778	.0365	.9491
MF4	.15	.45	.15	.05	.35	.0000	.6780	.0000	.0000	.3220	.1416	.9480	.0996	.3525	.1706	.0996	.2778	.0635	.9676
FM1	.00	.70	.10	.10	.20	.0000	.6780	.0000	.0000	.3220	.0841	.9535	.0996	.3525	.1706	.0996	.2778	.1684	.8778
FM2	.00	.75	.05	.15	.20	.0000	.6780	.0000	.0000	.3220	.0949	.9442	.0996	.3525	.1706	.0996	.2778	.1955	.8387
FM3	.00	.45	.20	.15	.30	.0000	.6780	.0000	.0000	.3220	.1516	.8967	.0996	.3525	.1706	.0996	.2778	.0683	.9415
FM4	.00	.65	.05	.15	.25	.0000	.6780	.0000	.0000	.3220	.0787	.9652	.0996	.3525	.1706	.0996	.2778	.1525	.8783
MI1	.10	.00	.50	.15	.30	.0903	.0783	.3631	.1463	.3220	.0714	.9527	.1225	.1225	.3525	.0996	.3031	.0892	.9142
MI2	.20	.15	.40	.05	.30	.0903	.0783	.3631	.1463	.3220	.0752	.8178	.1225	.1225	.3525	.0996	.3031	.0479	.9346
MI3	.10	.10	.45	.10	.30	.0903	.0783	.3631	.1463	.3220	.0464	.9561	.1225	.1225	.3525	.0996	.3031	.0459	.9790
MI4	.15	.10	.45	.10	.30	.0903	.0783	.3631	.1463	.3220	.0533	.9339	.1225	.1225	.3525	.0996	.3031	.0464	.9715
IM1	.10	.25	.30	.10	.30	.0903	.0783	.3631	.1463	.3220	.0850	.7089	.1225	.1225	.3525	.0996	.3031	.0625	.8141
IM2	.00	.20	.45	.05	.30	.0903	.0783	.3631	.1463	.3220	.0897	.8462	.1225	.1225	.3525	.0996	.3031	.0812	.9087
IM3	.05	.15	.50	.05	.35	.0903	.0783	.3631	.1463	.3220	.0843	.9223	.1225	.1225	.3525	.0996	.3031	.0805	.9758
IM4	.00	.25	.45	.10	.30	.0903	.0783	.3631	.1463	.3220	.0978	.7928	.1225	.1225	.3525	.0996	.3031	.0903	.8448
ME1	.15	.10	.10	.35	.40	.1238	.1659	.0000	.3883	.3220	.0672	.8887	.0996	.0996	.0996	.3525	.3489	.0321	.9811
ME2	.05	.35	.10	.25	.35	.1238	.1659	.0000	.3883	.3220	.1177	.6202	.0996	.0996	.0996	.3525	.3489	.1230	.5206
ME3	.10	.20	.15	.30	.35	.1238	.1659	.0000	.3883	.3220	.0810	.8464	.0996	.0996	.0996	.3525	.3489	.0555	.9229
ME4	.15	.15	.05	.35	.40	.1238	.1659	.0000	.3883	.3220	.0469	.9527	.0996	.0996	.0996	.3525	.3489	.0451	.9528
EM1	.05	.35	.20	.35	.15	.1238	.1659	.0000	.3883	.3220	.1486	.3498	.0996	.0996	.0996	.3525	.3489	.1515	.2150
EM2	.05	.50	.05	.30	.20	.1238	.1659	.0000	.3883	.3220	.1687	.4242	.0996	.0996	.0996	.3525	.3489	.1950	.1466
EM3	.05	.20	.15	.35	.35	.1238	.1659	.0000	.3883	.3220	.0792	.8356	.0996	.0996	.0996	.3525	.3489	.0549	.9101
EM4	.05	.35	.10	.25	.35	.1238	.1659	.0000	.3883	.3220	.1177	.6202	.0996	.0996	.0996	.3525	.3489	.1230	.5206
FF1	.05	.45	.10	.10	.30	.0000	.5975	.0000	.0000	.4025	.1047	.9900	.0996	.3525	.1706	.0996	.2778	.0591	.9719
FF2	.00	.35	.20	.25	.35	.0000	.5975	.0000	.0000	.4025	.1825	.7375	.0996	.3525	.1706	.0996	.2778	.0879	.7674
FF3	.10	.35	.10	.20	.35	.0000	.5975	.0000	.0000	.4025	.1575	.9170	.0996	.3525	.1706	.0996	.2778	.0637	.8438
FF4	.00	.55	.10	.15	.30	.0000	.5975	.0000	.0000	.4025	.0951	.9493	.0996	.3525	.1706	.0996	.2778	.1067	.9293
FI1	.00	.30	.35	.15	.30	.0796	.0690	.3200	.1289	.4025	.1196	.6037	.1225	.1225	.3525	.0996	.3031	.0991	.6741
FI2	.05	.40	.15	.20	.35	.0796	.0690	.3200	.1289	.4025	.1715	.1858	.1225	.1225	.3525	.0996	.3031	.1647	.0581
FI3	.05	.35	.25	.20	.30	.0796	.0690	.3200	.1289	.4025	.1416	.3530	.1225	.1225	.3525	.0996	.3031	.1246	.3292
FI4	.00	.55	.15	.05	.35	.0796	.0690	.3200	.1289	.4025	.2347	.0960	.1225	.1225	.3525	.0996	.3031	.2207	.1045
IF1	.15	.35	.25	.10	.30	.0796	.0690	.3200	.1289	.4025	.1415	.3136	.1225	.1225	.3525	.0996	.3031	.1123	.4121
IF2	.05	.40	.25	.20	.30	.0796	.0690	.3200	.1289	.4025	.1618	.2306	.1225	.1225	.3525	.0996	.3031	.1434	.2294
IF3	.00	.40	.30	.05	.35	.0796	.0690	.3200	.1289	.4025	.1583	.4601	.1225	.1225	.3525	.0996	.3031	.1410	.5305
IF4	.00	.25	.45	.05	.30	.0796	.0690	.3200	.1289	.4025	.1206	.6983	.1225	.1225	.3525	.0996	.3031	.0930	.8578
FE1	.00	.20	.15	.30	.45	.1715	.1486	.0000	.2774	.4025	.1071	.7327	.0996	.0996	.0996	.3525	.3489	.0843	.8389
FE2	.05	.15	.10	.40	.40	.1715	.1486	.0000	.2774	.4025	.0892	.8193	.0996	.0996	.0996	.3525	.3489	.0444	.9776
FE3	.05	.10	.15	.30	.45	.1715	.1486	.0000	.2774	.4025	.0921	.7907	.0996	.0996	.0996	.3525	.3489	.0600	.9179
FE4	.05	.10	.15	.25	.50	.1715	.1486	.0000	.2774	.4025	.0999	.7841	.0996	.0996	.0996	.3525	.3489	.0876	.8407
EF1	.15	.05	.15	.25	.45	.1715	.1486	.0000	.2774	.4025	.0845	.8071	.0996	.0996	.0996	.3525	.3489	.0752	.8384
EF2	.10	.15	.25	.25	.35	.1715	.1486	.0000	.2774	.4025	.1193	.5061	.0996	.0996	.0996	.3525	.3489	.0845	.7459
EF3	.05	.10	.20	.35	.35	.1715	.1486	.0000	.2774	.4025	.1141	.6160	.0996	.0996	.0996	.3525	.3489	.0501	.9211
EF4	.10	.15	.15	.30	.35	.1715	.1486	.0000	.2774	.4025	.0786	.8212	.0996	.0996	.0996	.3525	.3489	.0396	.9669
II1	.00	.10	.50	.05	.35	.0867	.0752	.3484	.1404	.3494	.0886	.9343	.1225	.1225	.3525	.0996	.3031	.0916	.9780
II2	.05	.05	.55	.05	.30	.0867	.0752	.3484	.1404	.3494	.1032	.9028	.1225	.1225	.3525	.0996	.3031	.1020	.9639
II3	.05	.15	.45	.10	.35	.0867	.0752	.3484	.1404	.3494	.0615	.9348	.1225	.1225	.3525	.0996	.3031	.0595	.9736
II4	.00	.05	.60	.05	.30	.0867	.0752	.3484	.1404	.3494	.1281	.8934	.1225	.1225	.3525	.0996	.3031	.1296	.9499
IE1	.10	.00	.20	.35	.40	.1866	.1618	.0000	.3022	.3494	.1253	.5936	.0996	.0996	.0996	.3525	.3489	.0673	.8991
IE2	.05	.15	.25	.25	.45	.1866	.1618	.0000	.3022	.3494	.1372	.4497	.0996	.0996	.0996	.3525	.3489	.0983	.7341
IE3	.00	.10	.35	.20	.40	.1866	.1618	.0000	.3022	.3494	.1866	.0700	.0996	.0996	.0996	.3525	.3489	.1404	.4871
IE4	.05	.20	.20	.30	.35	.1866	.1618	.0000	.3022	.3494	.1096	.5517	.0996	.0996	.0996	.3525	.3489	.0713	.8312
EI1	.00	.20	.30	.25	.30	.1866	.1618	.0000	.3022	.3494	.1621	.0403	.0996	.0996	.0996	.3525	.3489	.1209	.4753
EI2	.05	.05	.35	.30	.35	.1866	.1618	.0000	.3022	.3494	.1753	.1215	.0996	.0996	.0996	.3525	.3489	.1186	.6113
EI3	.00	.10	.30	.25	.40	.1866	.1618	.0000	.3022	.3494	.1637	.2468	.0996	.0996	.0996	.3525	.3489	.1124	.6543
EI4	.05	.30	.20	.30	.25	.1866	.1618	.0000	.3022	.3494	.1324	.2809	.0996	.0996	.0996	.3525	.3489	.1142	.4858
EE1	.15	.05	.20	.30	.35	.2670	.1214	.0000	.1766	.4349	.1275	.5308	.0996	.0996	.0996	.3525	.3489	.0597	.8780
EE2	.15	.10	.20	.30	.40	.2670	.1214	.0000	.1766	.4349	.1188	.6257	.0996	.0996	.0996	.3525	.3489	.0600	.9070
EE3	.10	.00	.20	.30	.45	.2670	.1214	.0000	.1766	.4349	.1401	.5741	.0996	.0996	.0996	.3525	.3489	.0812	.8596
EE4	.15	.00	.20	.35	.40	.2670	.1214	.0000	.1766	.4349	.1412	.5334	.0996	.0996	.0996	.3525	.3489	.0709	.8807
Mean											.1233	.7675						.0927	.8591
